# Discovery of novel theophylline derivatives bearing tetrazole scaffold for the treatment of Alzheimer's disease†

**DOI:** 10.1039/d5ra00488h

**Published:** 2025-03-04

**Authors:** Nguyen Viet Hung, Le Quoc Tien, Vu Ngoc Hai Linh, Hoang Tran, Tiep K. Nguyen, Duc-Vinh Pham, Van-Hai Hoang, Tran Thi Thu Hien, Thanh Xuan Nguyen, Quynh Mai Thai, Trung Hai Nguyen, Son Tung Ngo, Phuong-Thao Tran

**Affiliations:** a Hanoi University of Pharmacy 13-15 Le Thanh Tong Hanoi 11021 Vietnam thaotp119@gmail.com/thaotp@hup.edu.vn; b Hanoi University of Mining and Geology 18 Vien, Bac Tu Liem Hanoi 11910 Vietnam; c Faculty of Pharmacy, PHENIKAA University Hanoi 12116 Vietnam; d Vietnam University of Traditional Medicine 2 Tran Phu, Ha Dong Hanoi 100000 Vietnam; e Department of Surgical Oncology, Viet-Duc University Hospital Hanoi 100000 Vietnam; f Laboratory of Biophysics, Institute of Advanced Study in Technology, Ton Duc Thang University Ho Chi Minh City 72915 Vietnam; g Faculty of Pharmacy, Ton Duc Thang University Ho Chi Minh City 72915 Vietnam

## Abstract

Alzheimer's disease (AD) is associated with AChE and BACE1 enzymes. Designing inhibitors for preventing these enzymes can be benefit for AD treatment. In this context, theophylline derivatives were generated to prevent the biological activity of AChE and BACE1. In particular, the potential inhibitory of these compounds was rapidly and accurately estimated *via* knowledge-methods. The *in vitro* tests were then performed to validate the artificial intelligent approach. Among these, compound 12 exhibited the most potent AChE inhibition with an IC_50_ of 15.68 μM, while showing limited activity against BACE1. In addition, six compounds were indicated that are able to inhibit AChE, however, the theophylline derivatives play poor performance over the BACE1 target. Atomistic simulations were finally applied to clarify the ligand-binding mechanism to the biological target. The outcomes disclose that theophylline derivatives rigidly form van der Waals interactions to AChE *via* π-stacking and SC contacts. Overall, the theophylline derivatives may offer a potential scaffold for novel anti-AD agents.

## Abstract 2

A combined *in silico* and *in vitro* approach to the discovery of novel theophylline derivatives bearing a tetrazole scaffold for the treatment of Alzheimer's disease.

## Graphical abstract

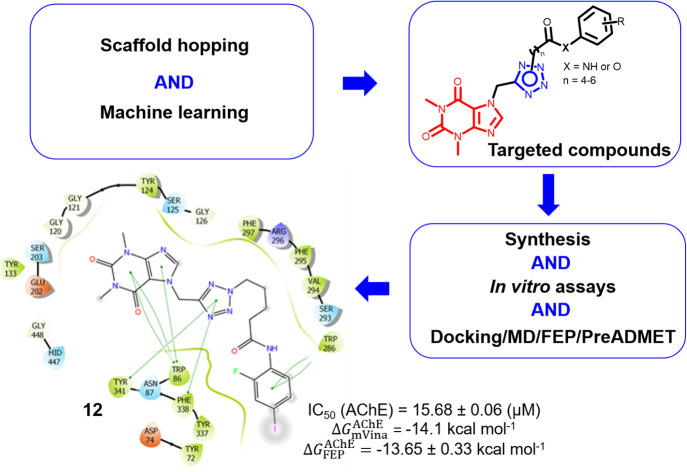

## Introduction

Alzheimer's disease (AD) is a multifaceted neurodegenerative disorder, commonly associated with progressive cognitive decline (impairment), memory loss and sudden behavioral shifts. Research shows that individuals diagnosed with Alzheimer's dementia at age 65 or older typically live for an average of four to eight years, although some may survive up to 20 years.^1^ As of today, an estimated 6.9 million Americans aged 65 and older are living with Alzheimer's dementia, a figure that is projected to rise significantly to 13.8 million by 2060, barring the medical advancements to prevent or cure the disease. In 2020 and 2021, amid the COVID-19 pandemic, Alzheimer's disease remained a leading cause of death, ranking seventh in the United States.^1^ Despite extensive research into AD-type dementia, current treatments offer only temporary and limited effects.^2^

Several hypotheses have been proposed for the underlying cause of Alzheimer's disease, focusing on different pathological mechanisms. Some key hypotheses including amyloid-beta, cholinergic, and metal ion hypotheses.^3,4^ Despite ongoing research, only a limited number of drugs for Alzheimer's have been approved by the FDA and EMA. These includes: galantamine, rivastigmine, donezepil, lecanemab, donanemab, aducanumab.^5,6^ Most available treatments focus on the cholinergic hypothesis, which highlights the role of acetylcholine (ACh) in cognitive function. Thus, targeting of AChE emerges as a highly promising therapeutics strategy for the treatment of AD. The binding site of AChE is divided into three distinct regions, including the catalytic active site (CAS), the binding gorge, and the peripheral anionic site (PAS). An effective AChE inhibitor must either bind to the CAS or PAS region, or possess a linker of suitable length and structure that bridges these two sites, allowing interaction with both, especially have two aromatic residues.^7^ In addition, it is well-established that AD is associated with the self-assembly of Aβ peptides, a cornerstone of the amyloid cascade hypothesis. In the amyloidogenic pathway, the amyloid precursor protein (APP), a transmembrane protein, undergoes proteolytic cleavage by β- and γ-secretases, resulting in the generation of Aβ peptides.^8^ Therefore, targeting β-secretase (BACE-1) inhibition presents a promising therapeutic strategy for preventing AD.

**Fig. 1:**
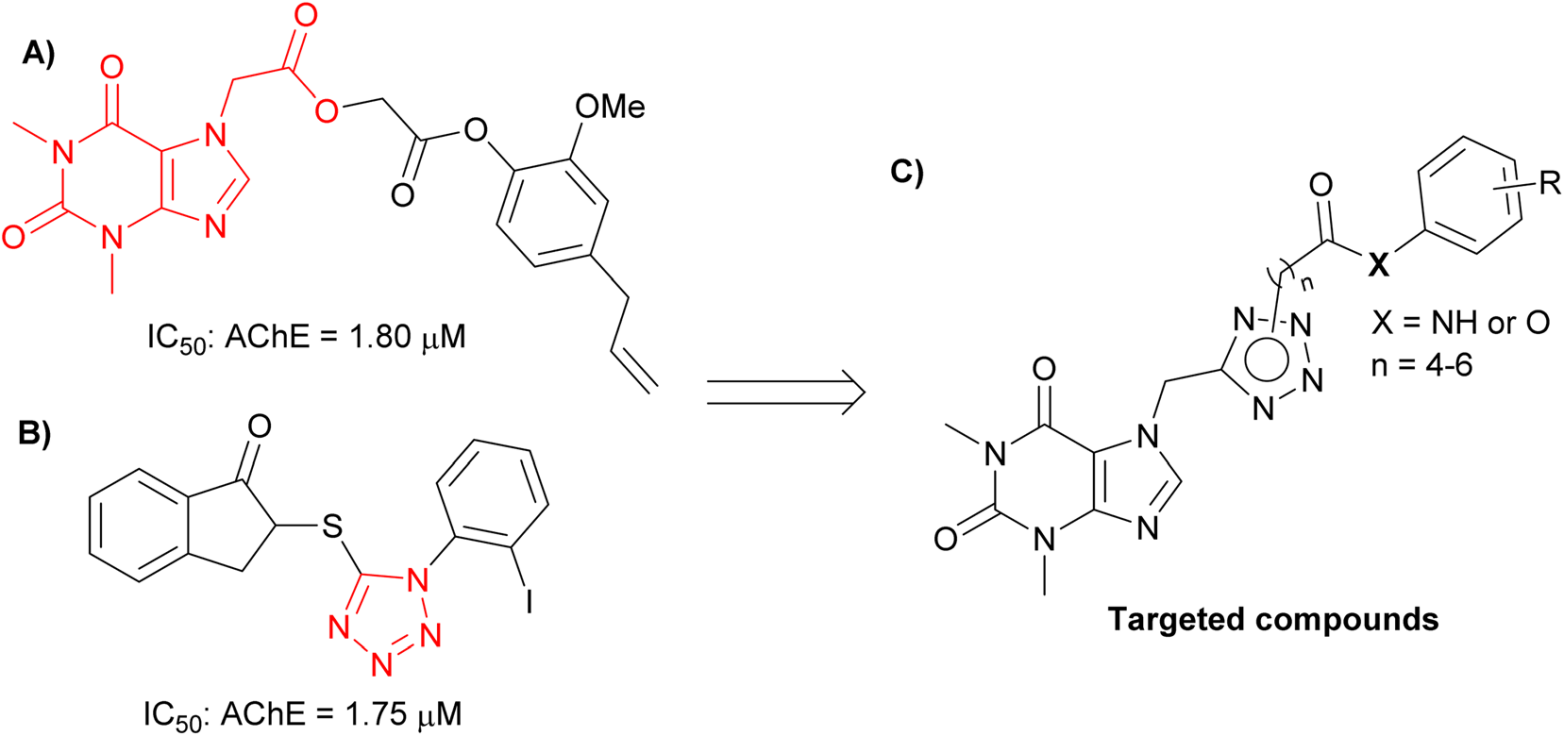
(A) Structure of theophylline compound as an AChE inhibitor; (B) structure of tetrazol compound as an AChE inhibitor; (C) design targeted compounds.

Theophylline, a methylxanthine drug, has been associated with a reduced risk of neurodegenerative diseases, including AD.^9^ The ability of this compound to cross the blood–brain barrier positions them as attractive scaffolds for the development of neuroprotective agents. Theophylline has been widely studied to develop AChE inhibitory compounds because its derivatives are capable of inhibiting AChE to the micromolar range (Fig. 1A),^9^ and also reduce Aβ aggregation.^10^ In addition, tetrazole is heterocyclic contain four nitrogen atoms, and tetrazole-bearing compounds have shown potential in inhibiting AChE with IC_50_ values in the micromolar range (Fig. 1B).^11^

In the present study, we employed a theophylline-tetrazole scaffold as a core aromatic residue, linked with various phenyl derivatives by alkylamide or alkylester linkages, to design, synthesize, and evaluate novel compounds (Fig. 1C) for their inhibitory activity against AChE and BACE-1. The objective was to expand the repertoire of potential AChE and BACE-1 inhibitors. The ligand-binding affinity of these compounds was subsequently characterized through both computational and experimental approaches. Furthermore, their drug-likeness properties were assessed to identify privileged scaffolds for the development of innovative anti-AD agents.

## Materials & methods

### Chemistry

Reagents sourced from domestic and international suppliers (Merck, Sigma-Aldrich) were employed without further purification. Solvents were procured from Merck, Sigma-Aldrich, Fisher, and suppliers in South Korea and China. The progression of reactions was monitored *via* thin layer chromatography (TLC) using Merck Kieselgel 60F_254_ plates, visualized under UV light at 254 nm.

#### Procedure for synthesis of compound 2

A solution of theophylline (1) (5 mmol, 900.8 mg) in acetone (10 ml) was added K_2_CO_3_ (827.7 mg, 6 mmol) and KI (16.6 mg, 0.1 mmol). After stirring for further 15 min, 0.36 ml of chloroacetonitrile (6 mmol) was dropped slowly into the mixture. The reaction mixtures were again stirred at 60 °C for 4 h. After completion of the reaction, the resulting mixtures were evaporated under reduced pressure to give the residues, which were extracted with dichloromethane (DCM) (5 × 30 ml) and 30 ml of water. The organic layer was combined, dried over Na_2_SO_4_ and purified by chromatography on a silica gel column (EA/*n*-hexane = 1/1, v/v) to give an off-white solid 2.

#### General procedure for synthesis of compounds 3–6

A mixture of compound 2 (1 mmol) in dimethylsulfoxide (DMSO) (5 ml) was added NaN_3_ (97.5 mg, 1.5 mmol) and ZnCl_2_ (13.6 mg, 0.1 mmol). The resulting mixtures were stirred at 120 °C for 2 h, and checked by thin layer chromatography (TLC) with mobile phase as ethyl acetate (EA)/*n*-hexan (3/1, v/v), then halogenide ester derivatives (1.2 mmol) was added into the mixture. The reaction mixtures were again stirred at 90 °C for 12 h. After completion of the reaction, the resulting mixtures were cooled, and 40 ml solution of saturated Na_2_CO_3_ was added slowly, after which the solution was filtered through Celite and extracted with EA (30 ml), washed with water (3 × 50 ml), The organic layers were combined, and dried over Na_2_SO_4_, and purified by chromatography on a silica gel column (EA/*n*-hexane = 3/1, v/v) to give 3–6 as an off-white solid.

#### General procedure for synthesis of compounds 7–10

A solution of compounds 3–6 (1 equiv.) in methanol (10 ml) was added NaOH (2 equiv.), which was dissolved in 1 ml of water. The reaction mixtures were stirred at room temperature for 2 h and checked by TLC with mobile phase as EA/*n*-hexan (1/1, v/v). After completion of the reaction, the resulting mixtures were adjusted to pH = 2–3 by concentrated hydrochloric acid solution, evaporated under reduced pressure, and dried to give 7–10 as off-white solid.

#### General procedure for synthesis of compounds 11–27

A mixture of carboxylic acids 7–10 (1 equiv.) dissolved in DMF (0.5 ml), EDC.HCl (1.2 equiv.), HOBt (1.2 equiv.), DMAP (1.2 equiv.) and phenol/aniline derivatives (1.2 equiv.) in DCM (10 ml) was stirred at room temperature for 12 h. The reaction mixture was evaporated under reduced pressure and extracted with EA (30 ml), washed with water (3 × 50 ml), and dried over Na_2_SO_4_, evaporated under reduced pressure, and purified by chromatography on a silica gel column (EA/*n*-hexane = 3 : 1) to give 11–27 as off-white solid.

### Acetylcholinesterases inhibition assay

A SPL 96-well plate (Korea), 1000 μL and 200 μL tips (Germany), a 1000 μL and 200 μL micropipette (Nichiryo, Japan), AChE enzyme (Sigma-Aldrich, USA), acetylthiocholine iodide (ATCI, Sigma-Aldrich, USA), 5,5-dithiobis(2-nitrobenzoic acid) (DTNB, TCI, Japan), and galantamine hydrobromide (TCI, Japan) were employed to evaluate the AChE inhibitory activity of the synthesized compounds using the modified Ellman's method.^12,13^ AChE inhibition was assessed on a Varioskan Lux 96-well plate reader. Each compound was tested at five concentrations (256 μM to 16 μM) in triplicate. The percentage inhibition was calculated using the standard formula.% inhibition = [1 − (test sample − blank)/(control − blank)] × 100%In which: blank: ATCI, DNTB in pH 8.0 buffer solution control: ATCI, DTNB and enzyme in pH 8.0 buffer test sample: test substance (or galantamine hydrobromide control), ATCI, DTNB and enzyme in pH 8.0 buffer solution. The IC_50_ values were determined using TableCurve 2Dv4 software.

### Beta secretasae 1 inhibition activity

BACE1 inhibitory activity of synthesized compounds (11–27) was examined using the BACE1 Inhibitor Screening Kit (Beyotime Biotech, Shanghai, China) according to the manufacturer's instructions. Briefly, BACE1 was incubated with various concentrations of compounds for 5 min at 37 °C in a black 96-well plate, followed by adding a specific fluorescence resonance energy transfer (FRET) substrate into the reaction mixture. The cleavage of FRET substrate was monitored through measurement of fluorescent intensity at 325/393 nm every 3 min for 30 min using a multimode microplate reader (VarioskanLux, ThermoScientific). The inhibitory percentage of BACE1 activity was calculated as following: *I*(%) = (ΔFI_control_ − ΔFI_compound_) × 100/ΔFI_control_, in which ΔFI denotes the change of fluorescent intensity per min. The inhibitory activity of compounds was initially screened at a fixed concentration of 200 μM.

### Machine learning calculation

Machine learning (ML) models were employed to fast and accurate predict the ligand-binding free energy to AChE and BACE1 targets, which were developed previously.^14,15^ Two models use the XGBoost algorithm that adopted high Pearson correlation coefficient, *R* = 0.8 ± 0.03 and *R* = 0.77 ± 0.02, with the respective experiments, respectively.

### Molecular docking simulation

AutoDock Vina^16^ (Vina) using altered empirical parameters^17^ (mVina) were used to rapidly evaluate the ligand-binding affinity and pose to AChE enzyme. In particular, the AChE structure was downloaded from the Protein Data Bank with identify of 4EY7.^18^ The Open Babel package^19^ was employed to generate the structure of theophylline derivatives. The AutoDockTools^20^ was first applied to parameterize the protein and ligand. The docking grid size was selected as 24 × 24 × 24 Å, which center of grid was chosen as the center of mass of the native inhibitors. The docking exhaustiveness was chosen as 8 due to the previous test.^17^ The ligand-binding pose was selected according to the lowest binding energy.

### MD simulation

The interaction of the AChE + inhibitors and free inhibitors with solution can be evaluated by using atomistic simulation *via* GROMACS version 2019.^21^ Due to previous benchmarks,^22,23^ the Amber99SB-iLDN force field^24^ was used to model the AChE enzyme and the neutralized ions. In combination with the Amber99SB-iLDN force field, the TIP3P water model^25^ was utilized to topologize the water molecule. Besides, the general Amber force field (GAFF)^26^ was utilized to parameterize the ligands. In particular, the chemical information of ligands was obtained by ACPYPE^27^ and AmberTools18 (ref. 28) approaches from the outcome of quantum calculations using B3LYP at 6-31G(d,p) level of theory.

The AChE + ligand system was put into a dodecahedron periodic boundary conditions (dPBC) box with a size of *ca.* 940 nm^3^ that comprised of *ca.* 92 000 atoms totally. The free inhibitor was put into the dPBC box with a size *ca.* 60 nm^3^ consisting of 6000 atoms totally. Initially, the steepest descent method was energetically minimized. The minimized conformation was then relaxed during NVT and NPT simulations. It should be noted that all atoms of the complex was positionally restrained during NVT simulations *via* a small harmonic force. The non-bonded interaction would be counted within a range of 9.0 Å. In particular, the van der Waals and electrostatics interactions were treated *via* the cut-off scheme and particle mesh Ewald method.

### Free energy perturbation simulation

The free energy perturbation method^29,30^ was executed to obtain the binding free energy between inhibitors and AChE. In particular, the inhibitor was turned from full-interaction to non-interaction two times, in solvated complex and in free ligand in solution, *via* the coupling parameter *λ*. During which, each simulation is length of 3.0 ns. More details was described in the previous work.^31^

### Analysis tools

A webapp server of ChemAxon, chemicalize,^32^ was carried out to predict the inhibitor protonation states. The principal component analysis (PCA)^33^ was performed to calculate first and second principal components, which were used to construct the free energy landscape (FEL) of non-hydrogen atoms of the AChE active site and ligands. The representative conformation of the AChE + ligand system was found *via* the clustering method,^34,35^ which was integrated in a tools of GROMACS “gmx cluster”. The interaction diagrams of AChE and BACE1 to ligand were prepared by using the Maestro free version.^36^

### Physicochemical properties and drug-likeness predictions

Some physicochemical properties and drug-likeness predictions of synthesized compounds were predicted by using the SwissADME platform.^37^ A potential oral drug should observe the criteria of Lipinski's rule of five^38^ and Veber's rule.^39^ The PreADMET web-server^40^ was employed to assess the metrics corresponding to the capability of the most potential compound to be able across blood–brain barrier (BBB) and human intestinal absorption (HIA) as well as the toxicity of this compound.

## Results and discussion

### ML calculations

Developing possible inhibitors to obstruct the biological action of AChE remains a significant focus,^41–43^ particularly as this enzyme is the most effective target for treating Alzheimer's disease to far.^44–48^ Recently, ML models are usually generated to accurately and rapidly evaluate the ligand-binding affinity.^49^ As mentioned above, theophylline derivatives (see Fig. 1C) were generated in order to prevent the activity of AChE and BACE1 enzymes.^15,50^

The designed compounds based on theophylline molecule were fast and accurately assessed the ligand-binding affinity, Δ*G*_ML_, using the trained ML models of AChE and BACE1 targets. The outcomes were shown in Table S1 of the ESI.† The predicted ligand-binding affinity to AChE and BACE1 range from −10.56 to −8.80 and −10.07 to −9.05 kcal mol^−1^, respectively. Moreover, for AChE target, ML model using the XGBoost method adopted a value of RMSE = 1.36 ± 0.10 kcal mol^−1^ and correlation coefficient of *R* = 0.81 ± 0.03.^50^ Furthermore, the corresponding metrics of the BACE1 target are of RMSE = 1.02 + 0.05 kcal mol^−1^ and *R* = 0.77 ± 0.02. The model was also based on the XGBoost method.^15^ It may thus be argued that all of designed compounds are able to inhibit the biological activity of AChE and BACE1 enzymes.

### Chemistry

**Scheme 1:**
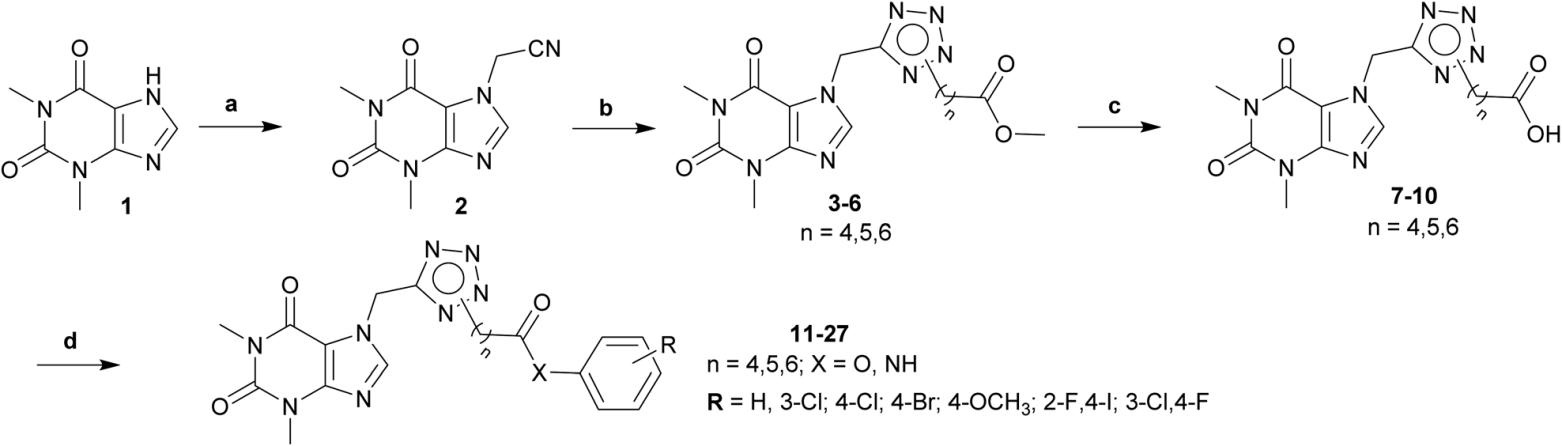
General procedure for synthesis final compounds 11–27. Reagents and conditions: (a) ClCH_2_CN, K_2_CO_3_, KI, acetone, 60 °C, 4 h; (b) (i) NaN_3_, ZnCl_2_, DMSO, 120 °C, 2 h; (ii) halogenide-ester derivatives, DMSO, 90 °C, 12 h; (c) (i) NaOH, MeOH, rt, 2 h; (ii) HCl to pH = 2–3; (d) aniline/phenol derivatives, EDC·HCl, DMAP, HOBt, DCM, DMF, rt to 50 °C, 12 h.

The synthesis of theophylline bearing tetrazol scaffold derivatives was illustrated in Scheme 1. Briefly, theophylline was alkylated by chloroacetonitrile using potassium carbonate and potassium iodide (catalyst) in acetone to form 2. Ester 3–6 prepared through click reaction using sodium azide and ZnCl_2_ in *N*,*N*-dimethylformamide (DMF), then alkylation with halogenide-ester derivatives. The structure of isomers were confirmed by 2D-NMR before carrying out hydrolysis by sodium hydroxide in methanol to form acid 7–10. Next, the amide or ester derivatives 11–27 were obtained through EDC/HOBt coupling with aniline or phenol derivatives.

### AChE and BACE1 inhibition activity

Based on the ML results (Table S1†), seventeen theophylline derivatives were selected, synthesized, and evaluated AChE and BACE-1 inhibition activities (Table 1). Most of the synthesized compounds showed moderate to low activity on AChE with the IC_50_ value below 200 μM compared with a IC_50_ value of 2.50 μM of positive control, galatamine hydrobromide. In particular, compound 12 with 4-carbon linker and substituent groups 2-F–4-I on the phenyl ring was the most potent with a IC_50_ value of 15.68 μM. The substitution at 4 positions on the phenyl ring was seen better for the activity on all cases of the linker. Specially, compounds bearing a 4-bromo substituent (15, 22, 25) demonstrated better AChE inhibitory activity relative to those substituted with 3-Cl, 4-Cl, or 4-OCH_3_ groups. These findings suggest that the 4-bromo substituent may be particularly well-suited for interactions with the AChE active site. For the 4-carbon linker, compound 11 with non-substituted group on the phenyl ring displayed an IC_50_ value of 110.31 μM. And the IC_50_ value decreased about seven folds when 2-F–4-I groups were on the phenyl ring (compound 12, IC_50_ = 15.68 μM). In the case of 5-carbon liker, the activity of compound 14 was improved from 153.43 μM to 84.07–114.98 μM (compounds 15–17) when the 3-Cl was replaced by other substituents at 4 positions. Similarly, with the 6-carbon linker the values changed from 220.62 μM (compound 20) to 63.51–126.08 μM (compound 21–23), respectively. Subsequently, when *N*2 isomers were changed to *N*1 isomers (compound 25–27) their activities did not improve so much (IC_50_ = 55.35–143.49 μM). Similarly, changing amide to ester (compounds 13, 18, 19, 24) did not lead to the more potent compounds. For beta-secretase 1 (BACE1) inhibition activity, all synthesized compounds did not show significant inhibition of the enzyme at the concentration of 200 μM. None of them could inhibit up to 50% of the enzyme activity at the testing concentration. The results suggested that these compounds were highly selective for AChE (compared to BACE1).

**Table 1:** *In vitro* AChE and BACE1 inhibition activites of synthesized compounds (11–27)

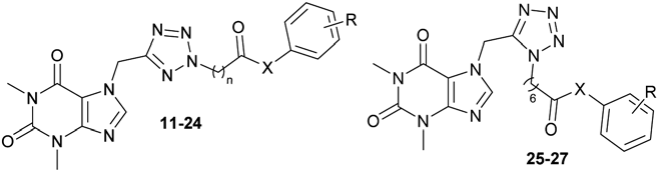					
Cpd	*n*	X	R	IC^AChE^_50_ (μM)	% Inhibition of BACE1 at 200 μM
11	4	NH	H	110.31 ± 17.53	−0.32 ± 2.05
12	4	NH	2-F, 4-I	15.68 ± 0.06	18.18 ± 3.9
13	4	O	4-OCH_3_	303.33 ± 34.06	1.40 ± 0.03
14	5	NH	3-Cl	153.43 ± 45.01	−2.01 ± 0.83
15	5	NH	4-Br	84.07 ± 20.19	8.94 ± 1.45
16	5	NH	3-Cl, 4-F	114.98 ± 10.56	−1.54 ± 1.19
17	5	NH	2-F, 4-I	104.69 ± 12.78	18.71 ± 2.26
18	5	O	H	94.36 ± 8.27	0.07 ± 3.57
19	5	O	4-OCH_3_	266.98 ± 30.37	−1.23 ± 0.35
20	6	NH	3-Cl	220.62 ± 37.73	10.72 ± 0.54
21	6	NH	4-Cl	126.08 ± 22.41	12.17 ± 0.47
22	6	NH	4-Br	63.51 ± 2.48	1.56 ± 3.43
23	6	NH	3-Cl, 4-F	109.94 ± 17.17	12.57 ± 0.45
24	6	O	4-OCH_3_	105.77 ± 25.73	0.74 ± 0.17
25	6	NH	4-Br	70.30 ± 0.10	−0.48 ± 0.76
26	6	NH	3-Cl, 4-F	55.35 ± 18.56	8.89 ± 0.49
27	6	NH	2-F, 4-I	143.49 ± 23.07	7.69 ± 0.86
Galantamine hydrobromide				2.50 ± 0.21	
Verubecestat					IC_50_ = 49.21 ± 7.20 (nM)

### Molecular docking simulations

Due to experimental results, physics-based methods including molecular docking and MD simulations were employed to clarify the physical insights into the ligand-binding process.^51^ In this context, the mVina package^51^ was suggested that form a successful-docking rate and correlation coefficient of 90 ± 10% and 0.86 ± 0.09, respectively.^51^ The obtained docking energy was described in Table 2 implying that the docking ligand affinity overestimates the ML calculations. It is in good agreement with the previous work that the mVina often adopts an overestimated outcome compared with experimental and ML results.^17,52^ Interestingly, six compounds almost form SC contacts to AChE-residues. Besides, the π-stacking interaction between six compounds to *Tpr86*, *Tpr286*, and *Tyr341* (*cf.* Table S2 in ESI†). There is no HB contact between two molecules was found. The outcomes imply that vdW interaction may plays an important role in the ligand binding process.

**Table 2:** The *in silico* binding energy of compounds 12, 15, 18, 22, 25, and 26 to AChE^a^

Cpd	*n*	X	R	Δ*G*^AChE^_mVina_	Δ*G*^AChE^_cou_	Δ*G*^AChE^_vdW_	Δ*G*^AChE^_FEP_
12	4	NH	2-F, 4-I	−14.1	0.14	−13.79	−13.65 ± 0.33
15	5	NH	4-Br	−14.6	−0.15	−17.76	−17.91 ± 1.03
18	5	O	H	−13.5	−1.78	−13.33	−15.11 ± 0.87
22	6	NH	4-Br	−15.2	−2.24	−15.39	−17.63 ± 1.30
25	6	NH	4-Br	−14.3	−5.17	−19.78	−24.95 ± 2.54
26	6	NH	3-Cl, 4-F	−14.7	−1.84	−10.39	−12.23 ± 4.44

a The unit of energy is of kcal mol^−1^.

### Atomistic simulations

To rapidly gain the outcomes, molecular docking simulations often apply several computational restraints such as using united-atoms, rigid receptors, *etc.*^20,53^ Therefore, MD simulations were utilized to validate the molecular docking results.^50,54^ The ligand-binding mechanisms can be obtained.^55,56^ In this work, the AChE + ligand complexes were thus mimicked in solution starting from docking conformation. Each MD simulation was 200 ns and repeated two times. During conventional MD simulations, the AChE + ligand complexes archive the equilibrium domains after *ca.* 75 ns (*cf.* Tables S3 and S4 of the ESI†).

**Fig. 2:**
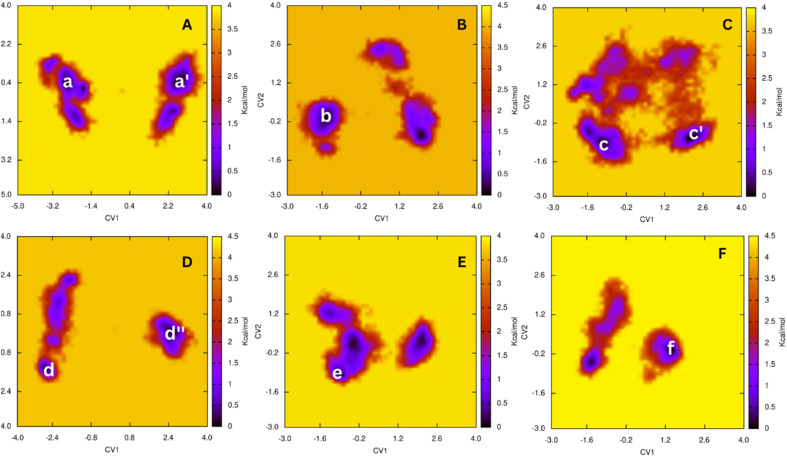
FELs of six complexes AChE + compounds over equilibrium conformations *via* PCA approach during interval 100–200 ns of MD simulations. In particular, (A–F) patterns are FEL of compounds 12, 15, 18, 22, 25, and 26, respectively.

Over equilibrium snapshots, analyzed tools were employed to discover the physical insights into the ligand-binding mechanism of theophylline derivatives to AChE enzyme.^57–59^ The popular conformations of the complexes were obtained by using a combination of FEL and clustering methods.^34,35^ In particular, the PCA approach^33^ was employed to calculate the first and second principal components, denoted as CV1 and CV2. The metrics were then used as two reaction coordinates to construct the FEL. The obtained FEL was then provided and shown in Fig. 2. The minima of the AChE – ligand complexes corresponding to the most popular conformations was denoted as a, a′, b, c, c′, d, d′, e and f, respectively. In details, the corresponding coordinates (CV1, CV2) of the minima is (−2.71, 0.31), (2.59, 0.42), (−1.48, −0.15), (−1.18, −1.02), (2.04, −0.76), (−2.88, −1.60), (2.40, 0.00), (−1.04, −1.04), and (1.26, −0.20), respectively. The population of the minima at the zero free energy is of 4.6, 1.9, 2.9, 1.8, 9.2, 3.5, 5.9, 2.1, and 10.8%, respectively.

**Fig. 3:**
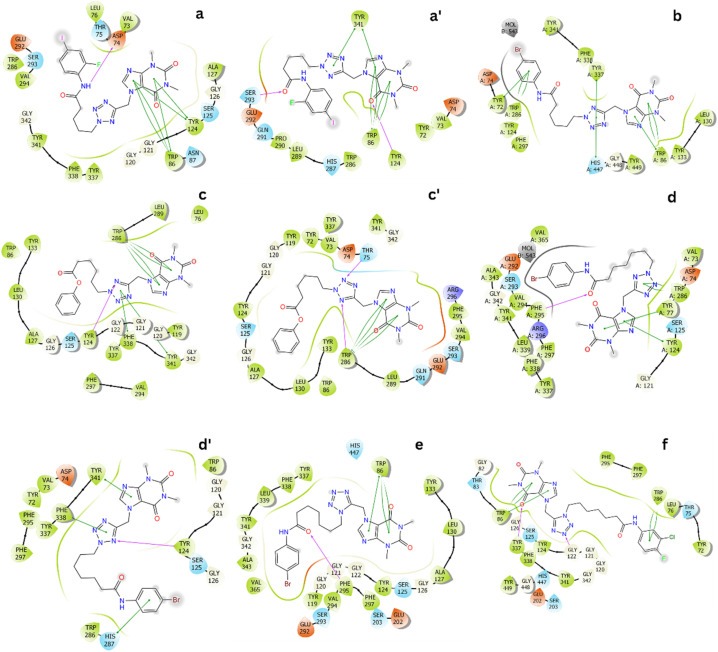
Two-dimension ligand-binding poses of AChE – theophylline derivatives matching to the minima a, a′, b, c, c′, d, d′, e and f. The diagram were generated *via* the Maestro free version.^36^

The representative conformations of the AChE – ligand complexes were then searched using the clustering method.^34,35^ In particular, the typical structure was archived *via* 0.3 nm non-hydrogen atom RMSD estimation. The two-dimension ligand-binding pose was thus analyzed and described in Fig. 3. In particular, theophylline derivatives adopt rigid π-stacking contacts to residues tryptophan and tyrosine such as *Trp86*, *Tyr124*, *Trp286*, *Tyr337*, and *Tyr341*. Besides, HB contacts between ligands and AChE were mostly measured between residues tyrosine including *Tyr124* and ligands.

In addition, ligands form SC contacts to more than 20 residues of the AChE. The obtained results are consistent with perturbation simulations, as mentioned below, in which indicated that ligands form rigid vdW interactions to AChE.

### Perturbation calculation

The perturbation simulations were performed to calculate the binding free energy between AChE and theophylline derivatives. The last structures of the complexes were used as initial conformation of the simulations. Besides, the last structures of free ligand in solution were also used as starting shape for *λ*-alteration simulations. The ligand-binding free energy of theophylline derivatives to AChE was acquired and designated in Table 2. The outcomes, range from −12.23 ± 4.44 to −24.95 ± 2.54 kcal mol^−1^, are in good agreement with experiments that six compounds named 12, 15, 18, 22, 25, and 26 can be play as potential inhibitors for preventing the biological activity of AChE. Moreover, the vdW interaction free energy adopts in the range from −10.39 to −19.78 kcal mol^−1^ that rules the ligand-binding free energy since the electrostatic interaction free energy diffuses in the range from 0.14 to −5.17 kcal mol^−1^. The observation confirm the finding above when ligands mostly form SC and π-stacking contacts to AChE instead of HB one.

### Physicochemical properties and drug-likeness predictions

The synthesized compounds were predicted drug-likeness based on Linpinski's rule of five and Veber's rule through the physicochemical properties: the log *P*, molecular weight, number of rotatable bonds, number of hydrogen bonding acceptor/donors, and topological polar surface area (Table 3). None of them, except compound 24, violated more than one property indicating they can use the oral route with good bioavailability. Furthermore, the BBB and HIA values observed for compound 12 were comparable to those of galantamine (*cf.* Table S5 in ESI†). These data indicate that compound 12 may exhibit favorable BBB permeability and absorption from the human intestine, suggesting its potential suitability as a drug candidate.

**Table 3:** The ADME prediction of the AChE inhibitor compounds 11–27^a^

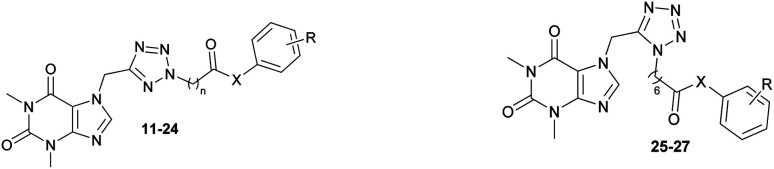										
Cpd	*n*	X	R	Formula	log *P*	MW	#Rot	#H-a	#H-d	TPSA (Å^2^)
11	4	NH	H	C_20_H_23_N_9_O_3_	0.95	437.46	9	7	1	134.52
12	4	NH	2-F, 4-I	C_20_F_21_FIN_9_O_3_	2.06	581.34	9	8	1	134.52
13	4	O	4-OCH_3_	C_21_H_24_N_8_O_5_	1.42	468.47	10	9	0	140.95
14	5	NH	3-Cl	C_21_H_24_ClN_9_O_3_	1.92	485.93	10	7	1	134.52
15	5	NH	4-Br	C_21_H_24_BrN_9_O_3_	2.00	530.38	10	7	1	134.52
16	5	NH	3-Cl, 4-F	C_21_H_23_ClFN_9_O_3_	2.22	503.92	10	8	1	134.52
17	5	NH	2-F, 4-I	C_21_H_23_FIN_9_O_3_	2.39	595.37	10	8	1	134.52
18	5	O	H	C_21_H_24_N_8_O_4_	1.72	452.47	10	8	0	131.72
19	5	O	4-OCH_3_	C_22_H_26_N_8_O_5_	1.75	482.49	11	9	0	140.95
20	6	NH	3-Cl	C_22_H_26_ClN_9_O_3_	2.15	499.95	11	7	1	134.52
21	6	NH	4-Cl	C_22_H_26_ClN_9_O_3_	2.38	499.95	11	7	1	134.52
22	6	NH	4-Br	C_22_H_26_BrN_9_O_3_	2.22	544.40	11	7	1	134.52
23	6	NH	3-Cl, 4-F	C_22_H_25_ClFN_9_O_3_	2.40	517.94	11	8	1	134.52
24	6	O	4-OCH_3_	C_23_H_28_N_8_O_5_	2.14	496.52	12	9	0	140.95
25	6	NH	4-Br	C_22_H_26_BrN_9_O_3_	2.18	544.40	11	7	1	134.52
26	6	NH	3-Cl, 4-F	C_22_H_25_ClFN_9_O_3_	2.38	517.94	11	8	1	134.52
27	6	NH	2-F, 4-I	C_22_H_25_FIN_9_O_3_	2.53	609.40	11	8	1	134.52
RO5**					≤5	≤500		≤10	≤5	
Veber							≤10	Total H-bond ≤12		≤140

a *TPSA: Topological Polar Surface Area; MW: Molecular Weight; #H-a: Hydrogen Bonding Acceptor; #H-d: Hydrogen Bonding Donor; #Rot: Number of Rotatable Bonds; **RO5: Lipinski's rule of five.

## Conclusions

Seventeen theophylline derivatives were designed, evaluated anti-AD through their *in vitro* AChE and BACE1 inhibitor ability. In which, compound 12 demonstrated the best activity on AChE with the IC_50_ of 15.68 μM but poor activity on BACE1. In addition, six compounds showed significant AChE inhibition with IC_50_ value less than 100 μM, while their activity against BACE1 was limited. Atomistic simulations revealed that theophylline derivatives interact strongly with AChE, primarily through van der Waals forces such as π-stacking and sulfur–carbon (SC) contacts. All of seventeen compounds passed the Linpinski's rule of five and Veber's rule for the orally drugs. These findings suggest that inhibiting AChE with theophylline derivatives may offer a potential therapeutic strategy for AD.

## Data availability

The data that support the findings of this study are available from the corresponding author, upon reasonable request.

## Author contributions

P.-T. Tran conceived and planned the experiments. N. V. Hung, L. Q. Tien, V. N. H. Linh, H. Tran, H.-V. Hai, and T. T. T. Hien carried out the synthetic experiments. T. K. Nguyen, D.-V. Pham, and T. X. Nguyen carried out the bio-assay experiments. S. T. Ngo, T. H. Nguyen, and Q. M. Thai planned and carried out the simulations. P.-T. Tran, N. V. Hung, L. Q. Tien, V. N. H. Linh, and H. Tran contributed to sample preparation. P.-T. Tran, N. V. Hung, H.-V. Hai, Q. M. Thai and S. T. Ngo contributed to the interpretation of the results. P.-T. Tran took the lead in writing the manuscript. All authors provided critical feedback and helped shape the research, analysis and manuscript.

## Conflicts of interest

There are no conflicts to declare.

## Supplementary Material

RA-015-D5RA00488H-s001

RA-015-D5RA00488H-s002
