# Supplementary material for: Discovery of novel theophylline derivatives bearing tetrazole scaffold for the treatment of Alzheimer's disease
Source: RSC Adv. 2025 Mar 4;15(9):6994–7003. doi: 10.1039/d5ra00488h (PMC11877286; doi:10.1039/d5ra00488h)
Supplement: RA-015-D5RA00488H-s002 [file RA-015-D5RA00488H-s002.pdf]

Supplementary Information (SI) for RSC Advances.
This journal is © The Royal Society of Chemistry 2025

SUPPORTING INFORMATION FOR

Discovery of novel theophylline derivatives bearing tetrazole scaffold for the treatment of Alzheimer's disease

Nguyen Viet Hung,[ab] Le Quoc Tien,[a] Vu Ngoc Hai Linh,[a] Hoang Tran,[a] Tiep K. Nguyen,[a] Duc-Vinh Pham,[a] Van-Hai Hoang,[c] Tran Thi Thu Hien,[d] Thanh Xuan Nguyen,[e] Quynh Mai Thai,[fg] Trung Hai Nguyen,[fg] Son Tung Ngo,[fg] Phuong-Thao Tran,[a]*

[a]Hanoi University of Pharmacy, 13-15 Le Thanh Tong, Hanoi 11021, Vietnam. E-mail: thaotp119@gmail.com/ thaotp@hup.edu.vn

[b]Hanoi University of Mining and Geology, 18 Vien, Bac Tu Liem, Hanoi 11910, Vietnam.

[c]Faculty of Pharmacy, PHENIKAA University, Hanoi 12116, Viet Nam.

[d]Vietnam University of Traditional Medicine, 2 Tran Phu, Ha Dong, Hanoi 100000, Vietnam

[e]Department of Surgical Oncology, Viet-Duc University Hospital, Hanoi 100000, Vietnam.

[f]Laboratory of Biophysics, Institute of Advanced Study in Technology, Ton Duc Thang University, Ho Chi Minh City 72915, Vietnam

[g]Faculty of Pharmacy, Ton Duc Thang University, Ho Chi Minh City 72915, Vietnam

MACHINE LEARNING RESULTS

Table S1. Binding energy caculated by Machine learning approach

Smiles	ΔG_{ML}^{AChE}	ΔG_{ML}^{BACE-1}
O=C1C2=C(N=CN2CC3=NN(CCCCCC(NC4=CC=C(I)C=C4F)=O)N=N3)N(C)C(N1C)=O	-10.409693	-10.068348
O=C1C2=C(N=CN2CC3=NN=NN3CCCCCC(NC4=CC=C(I)C=C4F)=O)N(C)C(N1C)=O	-10.4689455	-9.965019
O=C1C2=C(N=CN2CC3=NN(CCCCCCC(NC4=CC=C(I)C=C4F)=O)N=N3)N(C)C(N1C)=O	-10.293982	-10.011011
O=C1C2=C(N=CN2CC3=NN=NN3CCCCCCC(NC4=CC=C(I)C=C4F)=O)N(C)C(N1C)=O	-10.3300085	-9.90273
O=C1C2=C(N=CN2CC3=NN=NN3CCCCCCC(NC4=CC=C(F)C(Cl)=C4)=O)N(C)C(N1C)=O	-10.247373	-9.609058
O=C1C2=C(N=CN2CC3=NN=NN3CCCCCCC(NC4=CC=C(Br)C=C4)=O)N(C)C(N1C)=O	-10.555878	-9.291848
O=C1C2=C(N=CN2CC3=NN=NN3CCCCCCC(NC4=CC=C(Cl)C=C4)=O)N(C)C(N1C)=O	-10.514137	-9.29776
O=C1C2=C(N=CN2CC3=NN=NN3CCCCCCC(NC4=CC=C([N+]([O-])=O)C=C4)=O)N(C)C(N1C)=O	-10.271304	-9.487595
O=C1C2=C(N=CN2CC3=NN(CCCCC(NC4=CC=C(I)C=C4F)=O)N=N3)N(C)C(N1C)=O	-9.712008	-10.040927
O=C1C2=C(N=CN2CC3=NN=NN3CCCCCCC(NC4=CC=CC(Cl)=C4)=O)N(C)C(N1C)=O	-10.442992	-9.272117
O=C1C2=C(N=CN2CC3=NN(CCCCCCC(NC4=CC=C(Br)C=C4)=O)N=N3)N(C)C(N1C)=O	-10.146721	-9.50038
O=C1C2=C(N=CN2CC3=NN=NN3CCCCCCC(NC4=CC=CC=C4Cl)=O)N(C)C(N1C)=O	-10.513418	-9.109821
O=C1C2=C(N=CN2CC3=NN(CCCCCCC(NC4=CC=C(F)C(Cl)=C4)=O)N=N3)N(C)C(N1C)=O	-10.171043	-9.432928
O=C1C2=C(N=CN2CC3=NN=NN3CCCCCCC(NC4=CC=C(OC)C=C4)=O)N(C)C(N1C)=O	-9.823613	-9.704937
O=C1C2=C(N=CN2CC3=NN=NN3CCCCCC(NC4=CC=C(Br)C=C4)=O)N(C)C(N1C)=O	-10.400115	-9.093646
O=C1C2=C(N=CN2CC3=NN=NN3CCCCCC(NC4=CC=CC(Cl)=C4)=O)N(C)C(N1C)=O	-10.210088	-9.265701
O=C1C2=C(N=CN2CC3=NN=NN3CCCCCC(NC4=CC=C(F)C(Cl)=C4)=O)N(C)C(N1C)=O	-9.872982	-9.576952
O=C1C2=C(N=CN2CC3=NN(CCCCCCC(NC4=CC=C(Cl)C=C4)=O)N=N3)N(C)C(N1C)=O	-9.960075	-9.485219
O=C1C2=C(N=CN2CC3=NN=NN3CCCCCC(NC4=CC=CC=C4Cl)=O)N(C)C(N1C)=O	-10.272831	-9.135108
O=C1C2=C(N=CN2CC3=NN(CCCCCCC(NC4=CC=C([N+]([O-])=O)C=C4)=O)N=N3)N(C)C(N1C)=O	-9.940655	-9.465313
O=C1C2=C(N=CN2CC3=NN=NN3CCCCCC(NC4=CC=C([N+]([O-])=O)C=C4)=O)N(C)C(N1C)=O	-9.903742	-9.477954
O=C1C2=C(N=CN2CC3=NN=NN3CCCCCC(NC4=CC=C(Cl)C=C4)=O)N(C)C(N1C)=O	-10.233639	-9.105392
O=C1C2=C(N=CN2CC3=NN(CCCCCC(NC4=CC=C(F)C(Cl)=C4)=O)N=N3)N(C)C(N1C)=O	-9.896478	-9.413058
O=C1C2=C(N=CN2CC3=NN=NN3CCCCCC(NC4=CC=C(OC)C=C4)=O)N(C)C(N1C)=O	-9.806946	-9.49427
O=C1C2=C(N=CN2CC3=NN(CCCCCCC(NC4=CC=CC(Cl)=C4)=O)N=N3)N(C)C(N1C)=O	-9.971409	-9.307013
O=C1C2=C(N=CN2CC3=NN=NN3CCCCCCC(NC4=CC=C(F)C=C4)=O)N(C)C(N1C)=O	-9.841448	-9.420293
O=C1C2=C(N=CN2CC3=NN(CCCCCCC(NC4=CC=C(F)C=C4)=O)N=N3)N(C)C(N1C)=O	-9.65381	-9.602494
O=C1C2=C(N=CN2CC3=NN=NN3CCCCCCC(NC4=CC=C(OC)C=C4OC)=O)N(C)C(N1C)=O	-9.937963	-9.318114
O=C1C2=C(N=CN2CC3=NN(CCCCCCC(NC4=CC=CC=C4F)=O)N=N3)N(C)C(N1C)=O	-9.661625	-9.580032
O=C1C2=C(N=CN2CC3=NN=NN3CCCCCCC(NC4=CC=CC5=C4C=CC=C5)=O)N(C)C(N1C)=O	-9.926688	-9.291291
O=C1C2=C(N=CN2CC3=NN(CCCCCCC(NC4=CC=CC=C4Cl)=O)N=N3)N(C)C(N1C)=O	-10.036669	-9.180031
O=C1C2=C(N=CN2CC3=NN=NN3CCCCCC(NC4=CC=CC=C4F)=O)N(C)C(N1C)=O	-9.723577	-9.491406
O=C1C2=C(N=CN2CC3=NN(CCCCCC(NC4=CC=C(Br)C=C4)=O)N=N3)N(C)C(N1C)=O	-9.914809	-9.293579
O=C1C2=C(N=CN2CC3=NN=NN3CCCCCCC(NC4=CC=CC(F)=C4)=O)N(C)C(N1C)=O	-9.753476	-9.43736
O=C1C2=C(N=CN2CC3=NN=NN3CCCCCCC(NC4=CC=CC=C4F)=O)N(C)C(N1C)=O	-9.646044	-9.5428915
O=C1C2=C(N=CN2CC3=NN(CCCCCCC(NC4=CC=CC(F)=C4)=O)N=N3)N(C)C(N1C)=O	-9.598281	-9.569607
O=C1C2=C(N=CN2CC3=NN(CCCCCC(NC4=CC=CC=C4F)=O)N=N3)N(C)C(N1C)=O	-9.702657	-9.363819
O=C1C2=C(N=CN2CC3=NN(CCCCCC(NC4=CC=CC(Cl)=C4)=O)N=N3)N(C)C(N1C)=O	-9.755661	-9.306952
O=C1C2=C(N=CN2CC3=NN(CCCCCC(NC4=CC=CC(F)=C4)=O)N=N3)N(C)C(N1C)=O	-9.5940485	-9.454989
O=C1C2=C(N=CN2CC3=NN=NN3CCCCCC(NC4=CC=C(OC)C=C4OC)=O)N(C)C(N1C)=O	-9.763484	-9.238015
O=C1C2=C(N=CN2CC3=NN(CCCCCC(NC4=CC=CC=C4Cl)=O)N=N3)N(C)C(N1C)=O	-9.83854	-9.161812
O=C1C2=C(N=CN2CC3=NN=NN3CCCCCC(NC4=CC=CC5=C4C=CC=C5)=O)N(C)C(N1C)=O	-9.739863	-9.246615
O=C1C2=C(N=CN2CC3=NN(CCCCCC(NC4=CC=C(F)C=C4)=O)N=N3)N(C)C(N1C)=O	-9.627663	-9.353492
O=C1C2=C(N(C(N1C)=O)C)N=CN2CC3=NN(N=N3)CCCCCCC(NC4=CC=CC5=C4C=CC=C5)=O	-9.423247	-9.548932

O=C1C2=C(N=CN2CC3=NN=NN3CCCCCC(NC4=CC=C(F)C=C4)=O)N(C)C(N1C)=O	-9.805064	-9.129618
O=C1C2=C(N=CN2CC3=NN(CCCCCC(NC4=CC=C([N+]([O-])=O)C=C4)=O)N=N3)N(C)C(N1C)=O	-9.50172	-9.42738
O=C1C2=C(N=CN2CC3=NN(CCCCCC(NC4=CC=C(Cl)C=C4)=O)N=N3)N(C)C(N1C)=O	-9.684119	-9.243118
O=C1C2=C(N=CN2CC3=NN(CCCCCCC(NC4=CC=C(OC)C=C4OC)=O)N=N3)N(C)C(N1C)=O	-9.500409	-9.411681
O=C1C2=C(N=CN2CC3=NN=NN3CCCCCC(NC4=CC=CC(F)=C4)=O)N(C)C(N1C)=O	-9.707659	-9.196531
O=C1C2=C(N=CN2CC3=NN(CCCCCCC(NC4=CC=C(OC)C=C4)=O)N=N3)N(C)C(N1C)=O	-9.241608	-9.577578
O=C1C2=C(N=CN2CC3=NN(CCCCCC(NC4=CC=C(OC)C=C4OC)=O)N=N3)N(C)C(N1C)=O	-9.39806	-9.407064
O=C1C2=C(N(C(N1C)=O)C)N=CN2CC3=NN(N=N3)CCCCCC(NC4=CC=CC5=C4C=CC=C5)=O	-9.231118	-9.521998
O=C1C2=C(N=CN2CC3=NN=NN3CCCCCCC(NC4=CC=CC=C4)=O)N(C)C(N1C)=O	-9.670733	-9.074527
O=C1C2=C(N=CN2CC3=NN(CCCCC(NC4=CC=C(F)C(Cl)=C4)=O)N=N3)N(C)C(N1C)=O	-9.268599	-9.411899
O=C1C2=C(N=CN2CC3=NN=NN3CCCCCC(NC4=CC=CC=C4)=O)N(C)C(N1C)=O	-9.682138	-8.986335
O=C1C2=C(N=CN2CC3=NN(CCCCCC(NC4=CC=C(OC)C=C4)=O)N=N3)N(C)C(N1C)=O	-9.213857	-9.4022045
O=C1C2=C(N=CN2CC3=NN(CCCCC(NC4=CC=C(Br)C=C4)=O)N=N3)N(C)C(N1C)=O	-9.250253	-9.343806
O=C1C2=C(N=CN2CC3=NN=NN3CCCCCC(NC4=CC=CC=N4)=O)N(C)C(N1C)=O	-9.6973295	-8.875869
O=C1C2=C(N(C(N1C)=O)C)N=CN2CC3=NN(N=N3)CCCCC(NC4=CC=CC5=C4C=CC=C5)=O	-9.061124	-9.488653
O=C1C2=C(N=CN2CC3=NN=NN3CCCCCCC(NC4=CC=CC=N4)=O)N(C)C(N1C)=O	-9.628928	-8.899093
O=C1C2=C(N=CN2CC3=NN(CCCCC(NC4=CC=C(OC)C=C4)=O)N=N3)N(C)C(N1C)=O	-9.240488	-9.225705
O=C1C2=C(N=CN2CC3=NN(CCCCC(NC4=CC=C(F)C=C4)=O)N=N3)N(C)C(N1C)=O	-9.111293	-9.3255
O=C1C2=C(N=CN2CC3=NN(CCCCC(NC4=CC=CC(F)=C4)=O)N=N3)N(C)C(N1C)=O	-9.066723	-9.36249
O=C1C2=C(N=CN2CC3=NN(CCCCC(NC4=CC=C(OC)C=C4OC)=O)N=N3)N(C)C(N1C)=O	-9.020803	-9.37702
O=C1C2=C(N=CN2CC3=NN(CCCCC(NC4=CC=C(Cl)C=C4)=O)N=N3)N(C)C(N1C)=O	-9.137405	-9.220517
O=C1C2=C(N=CN2CC3=NN(CCCCC(NC4=CC=CC(Cl)=C4)=O)N=N3)N(C)C(N1C)=O	-9.119699	-9.222841
O=C1C2=C(N=CN2CC3=NN(CCCCC(NC4=CC=CC=C4F)=O)N=N3)N(C)C(N1C)=O	-9.052198	-9.27806
O=C1C2=C(N=CN2CC3=NN(CCCCC(NC4=CC=C([N+]([O-])=O)C=C4)=O)N=N3)N(C)C(N1C)=O	-9.101535	-9.223135
O=C1C2=C(N=CN2CC3=NN(CCCCC(NC4=CC=CC=C4Cl)=O)N=N3)N(C)C(N1C)=O	-9.252419	-8.997787
O=C1C2=C(N=CN2CC3=NN(CCCCCCC(NC4=CC=CC=N4)=O)N=N3)N(C)C(N1C)=O	-9.148231	-9.059104
O=C1C2=C(N=CN2CC3=NN(CCCCCC(NC4=CC=CC=N4)=O)N=N3)N(C)C(N1C)=O	-9.124372	-9.056039
O=C1C2=C(N=CN2CC3=NN(CCCCCC(NC4=CC=CC=C4)=O)N=N3)N(C)C(N1C)=O	-9.015229	-9.076412
O=C1C2=C(N=CN2CC3=NN(CCCCCCC(NC4=CC=CC=C4)=O)N=N3)N(C)C(N1C)=O	-8.969881	-9.117786
O=C1C2=C(N=CN2CC3=NN(CCCCC(NC4=CC=CC=N4)=O)N=N3)N(C)C(N1C)=O	-8.980835	-9.083343
O=C1C2=C(N=CN2CC3=NN(CCCCC(NC4=CC=CC=C4)=O)N=N3)N(C)C(N1C)=O	-8.820724	-9.052842
O=C1C2=C(N(C(N1C)=O)C)N=CN2CC3=NN(N=N3)CCCCCC(OC4=CC=CC=C4)=O	-8.85575	-9.355982
O=C1C2=C(N(C(N1C)=O)C)N=CN2CC3=NN(N=N3)CCCCC(OC4=CC=C(OC)C=C4)=O	-8.806358	-9.129724
O=C1C2=C(N(C(N1C)=O)C)N=CN2CC3=NN(N=N3)CCCCCC(OC4=CC=C(OC)C=C4)=O	-8.927311	-9.225198
O=C1C2=C(N(C(N1C)=O)C)N=CN2CC3=NN(N=N3)CCCCCCC(OC4=CC=C(OC)C=C4)=O	-8.86567	-9.398321

CHEMISTRY RESULTS

Detailed synthetic protocol

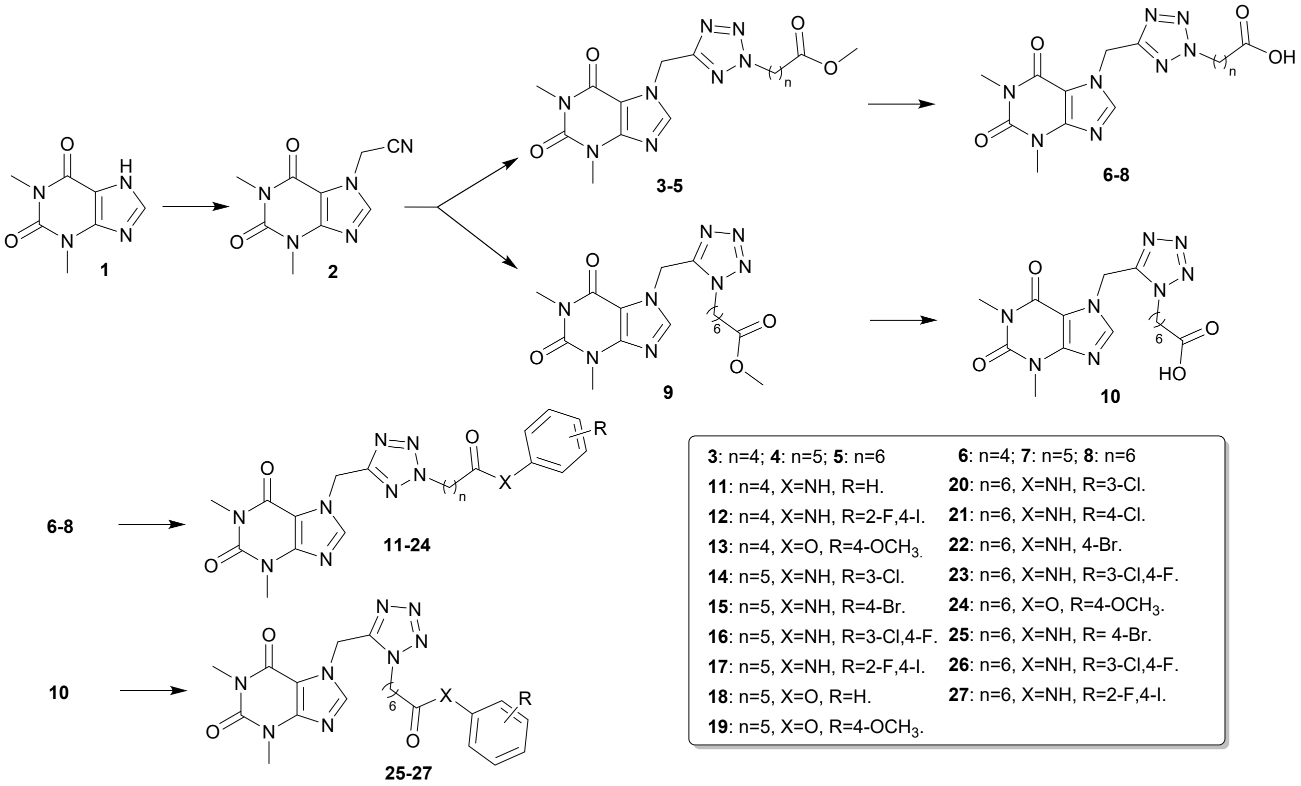

5-(5-((1,3-dimethyl-2,6-dioxo-1,2,3,6-tetrahydro-7H-purin-7-yl)methyl)-2H-tetrazol-2-yl)-N phenylpentanamide (11). Prepared from acid (**7**) and aniline following general procedure (d) to afford a off-white solid, yield: 60%. ^{1}H NMR (500 MHz, $CDCl_3$) *δ* ppm: 7.76 (s, 1H, H2''), 7.50 (m, 3H, CO-N<u>H</u>, H2', H6'), 7.31 (t, 2H, H3', H5', *J* = 8.0Hz), 7.10 (t, 1H, H4', *J* = 7.5 Hz), 5.82 (s, 2H, N1''-C<u>H$_2$</u>), 4.65 (t, 2H, H5, *J* = 7.0 Hz), 3.60 (s, 3H, N5''-C<u>H$_3$</u>), 3.36 (s, 3H, N7''-C<u>H$_3$</u>), 2.32 (t, 2H, H2, *J* = 7.0 Hz), 2.11 (quint, 2H, H4, *J* = 7.0 Hz), 1.77 (quint, 2H, H3, *J* = 7.5 Hz). ^{13}C NMR (125 MHz, $CDCl_3$) δ 170.130 (C=O), 161.523 (N=C-N), 155.286 (C8''), 151.620 (C6''), 148.853 (C4''), 141.653 (C2''), 137.845 (C1'), 129.034 (C3', C5'), 124.348 (C4'), 119.780 (C2', C6'), 106.934 (C9''), 53.198 (C5), 41.383 (N1''-<u>C</u>H$_2$), 36.226 (C2), 29.883 (N5''-<u>C</u>H$_3$), 28.396 (N7''-<u>C</u>H$_3$), 28.042 (C4), 22.063 (C3). HRMS (ESI) m/z calc. for $C_{20}H_{23}N_9O_3$ [M+H]$^+$ 438.1997, **found 438.1951; 301.1392**

***5-(5-((1,3-dimethyl-2,6-dioxo-1,2,3,6-tetrahydro-7H-purin-7-yl)methyl)-2H-tetrazol-2-yl)-N-(2-fluoro-4-iodophenyl)pentanamide* (*12*).** Prepared from acid (**7**) and 2-flouro-4-iodoaniline following general procedure (d) to afford a off-white solid, yield: 56.1%. ^{1}H NMR (500 MHz, $CDCl_3$) δ ppm 8.06 (s, 1H, H3'), 7.76 (s, 1H, <u>H2''</u>), 7.44 (m, 3H, CO-<u>NH,',</u> H5', H6'), 5.84 (s, 2H, N1''-C<u>H$_2$</u>), 4.65 (t, 2H, H5, *J* = 7.0 Hz), 3.60 (s, 3H, N5''-C<u>H$_3$</u>), 3.37 (s, 3H, N7''-C<u>H$_3$</u>), 2.41 (t, 2H, H2, *J* = 7.0 Hz), 2.11 (quint, 2H, H4, *J* = 7.0 Hz), 1.76 (quint, 2H, H3, *J* = 7.5 Hz). ^{13}C NMR ($CDCl_3$, 125 MHz) δ 170.106 (C=O), 161.513 (N=C-N), 155.203 (C8''), 151,626 (C6''), 152.763-150.792 (C2', $^1J_{C-F}$ = 246.48 Hz), 148.741 (C4''), 141.526 (C2''), 133.829-133.800 (C4', $^3J_{C-F}$ = 3.63 Hz), 126.150 (C1'), 124.049-123.878 (C3', $^2J_{C-F}$ = 21.38 Hz), 123.115 (C5'), 123.115 (C6'), 106.903 (C9''), 53.095 (C5), 41.247 <u>(N1'-<u>C</u>H$_2$),</u> 36.220 (C2), 29.848 (N5''-<u>C</u>H$_3$), 28.393 (N7''-<u>C</u>H$_3$), 27.986 (C4), 21.822 (C3). HRMS (ESI) m/z calc. for $C_{20}H_{21}FIN_9O_3$ [M+Na]$^+$ 604.0688, found 604.0682.

4-methoxyphenyl 5-(5-((1,3-dimethyl-2,6-dioxo-1,2,3,6-tetrahydro-7H-purin-7-yl)methyl)-2H-tetrazol-2-yl)pentanoate (13). Prepared from acid (**7**) and 4-methoxyphenol following general procedure (d) to afford a off-white solid, **yield: 63.1%** ^{1}H NMR (600 MHz, $CDCl_3$) δ ppm: 7.74 (s, 1H, H2''), 6.97 (dd, 2H, H2', H6', J_1 = 7.2 Hz, J_2 = 1.8 Hz), 6.88 (dd, 2H, H3', H5', J_1 = 5 Hz, J_2 = 2.4 Hz), 5.85 (s, 2H, N1''-C<u>H$_2$</u>), 4.66 (t, 2H, H5, *J* = 7.2 Hz), 3.80 (s, 3H, OC<u>H$_3$</u>), 3.59 (s, 3H, N5''-C<u>H$_3$</u>), 3.39 (s, 3H, N7''-C<u>H$_3$</u>), 2.58 (t, 2H, H2, *J* = 7.2 Hz), 2.13 (m, 2H, H4), 1.77 (m, 2H, H3). ^{13}C NMR (125 MHz, $CDCl_3$) δ 171.564 (C=O), 161.510 (N=C-N), 157.338 (C4'), 155.218 (C8''), 151.662 (C6''), 148.690 (C4''), 144.023 (C2''), 141.441 (C1'), 122.168 (C2', C6'), 114.495 (C3', C5'), 106.915 (C9''), 55.601 (O<u>C</u>H$_3$), 53.009 (C5), 41.112 (N1''-<u>C</u>H$_2$), 33.257 (C2), 29.793 (N5''-<u>C</u>H$_3$), 28.451 (N7''-<u>C</u>H$_3$), 27.958 (C4), 21.627 (C3). HRMS (ESI) m/z calc. for $C_{21}H_{24}N_8O_5$ [M+Na]$^+$ 491.1762, found 491.1723.

N-(3-chlorophenyl)-6-(5-((1,3-dimethyl-2,6-dioxo-1,2,3,6-tetrahydro-7H-purin-7-yl)methyl)-2H-tetrazol-2-yl)hexanamide (14). Prepared from acid (**8**) and 3-chloroaniline following general procedure (d) to afford a off-white solid, yield: 50.2%. ^{1}H NMR (500 MHz, $CDCl_3$) δ ppm 7.79 (s, 1H, H2''), 7.68 (s, 1H, H2'), 7.58 (s, 1H, CO-N<u>H</u>), 7.40 (d, 1H, H6', *J* = 8.0 Hz), 7.26 (t, 1H, H5', *J* = 8.0 Hz), 7.10 (d, 1H, H4', *J* = 7.0 Hz), 5.85 (s, 2H, N1''-C<u>H$_2$</u>), 4.63 (t, 2H, H6, *J* = 7.0 Hz), 3.63 (s, 3H, N5''-C<u>H$_3$</u>), 3.41 (s, 3H, N7''-C<u>H$_3$</u>), 2.32 (t, 2H, H2, *J* = 7.5 Hz), 2.05 (quint, 2H, H5, J_1 = 7.5 Hz, J_2 = 7.5 Hz), 1.76 (quint, 2H, H4, *J* = 7.5 Hz), 1.38 (quint, 2H, H3, *J* = 7.5 Hz). ^{13}C NMR (125 MHz, $CDCl_3$) δ 170.800 (C=O), 161.440 (N=C-N), 155.282 (C8''), 151.652 (C6''), 148.816 (C4''), 141.658 (C2''), 139.105 (C1), 134.663 (C3'), 130.001 (C5'), 124.268 (C4'), 119.815 (C2'), 117.626 (C6'), 106.914 (C9''), 53.159 (C6), 41.283 (N1''-CH$_2$), 37.106 (C2), 29.893 (N5''-<u>C</u>H$_3$), 28.766 (N7''-<u>C</u>H$_3$), 28.061 (C4), 25.816 (C5), 24.315 (C3). HRMS (ESI) m/z calc. for $C_{21}H_{24}{}^{35}ClN_9O_3$ [M+Na]$^+$ 508.1583, found 508.1579; calc. for $C_{21}H_{24}{}^{37}ClN_9O_3$ [M+Na]$^+$ 510.1553, found 510.1563.

N-(4-bromophenyl)-6-(5-((1,3-dimethyl-2,6-dioxo-1,2,3,6-tetrahydro-7H-purin-7-yl)methyl)-2H-tetrazol-2-yl)hexanamide (15). Prepared from acid (**8**) and 4-bromoaniline following general procedure (d) to afford a off-white solid, yield: 54.1%. ^{1}H NMR (500 MHz, $CDCl_3$) δ ppm 7.76 (s, 1H, H2''), 7.505 (s, 1H, CO-N<u>H)</u>, 7.43 (m, 4H, H2', H3', H5', H6'), 5.83 (s, 2H, N1''-C<u>H$_2$</u>), 4.61 (t, 2H, H6, *J* = 7.0 Hz), 3.60 (s, 3H, N5''-C<u>H$_3$</u>), 3.39 (s, 3H, N7''-C<u>H$_3$</u>), 2.29 (t, 2H, H2, *J* = 7.5 Hz), 2.03 (quint, 2H, H5, J_1= 7.5 Hz, J_2= 7.0 Hz), 1.74 (quint, 2H, H4, *J* = 7.5 Hz), 1.36 (t, 2H, H3, *J* = 7.5 Hz). ^{13}C NMR (125 MHz, $CDCl_3$) δ 170.691 (C=O), 161.439 (N=<u>C</u>-N), 155.275 (C8''), 151.650 (C6''), 148.810 (C48''), 141.632 (C2''), 137.035 (C1'), 131.974 (C3', C5'), 121.282 (C2', C6'), 116.778 (C4'), 106.909 (C9''), 53.157 (C6), 41.267 (N1''-C), 37.106 (C2), 29.886 (N5''-<u>C</u>H$_3$), 28.765 (N7''-<u>C</u>H$_3$), 28.052 (C5), 25.829 (C4), 24.323 (C3). HRMS (ESI) m/z calc. for $C_{21}H_{24}{}^{79}BrN_9O_3$ [M+Na]$^+$ 552.1078, found 552.1069; calc. for $C_{21}H_{24}{}^{81}BrN_9O_3$ [M+Na]$^+$ 554.1057, found 554.1058.

N-(3-chloro-4-fluorophenyl)-6-(5-((1,3-dimethyl-2,6-dioxo-1,2,3,6-tetrahydro-7H-purin-7-yl)methyl)-2H-tetrazol-2-yl)hexanamide (16). Prepared from acid (**8**) and 3-chloro-4-fluoro-bromoaniline following general procedure (d) to afford a off-white solid, yield: 61.6%. ^{1}H NMR (500 MHz, $CDCl_3$) δ ppm 7.77 (s, 1H, H2''), 7.73 (dd, 1H, H2', J_1 = 6.5 Hz, J_2 = 2.5 Hz), 7.63 (s, 1H, CO-N<u>H</u>'), 7.35 (t, 1H, H5'), 7.08 (t, 1H, H6', *J* = 8.5 Hz) 5.83 (s, 2H, N1''-C<u>H$_2$</u>), 4.61 (t, 2H, H6, *J* = 7.0 Hz), 3.61 (s, 3H, N5''-C<u>H$_3$</u>), 3.39 (s, 3H, N7''-C<u>H$_3$</u>), 2.29 (t, 2H, H2, *J* = 7.5 Hz), 2.03 (m, 2H, H5), 1.73 (quint, 2H, H4, *J* = 7.5 Hz), 1.34 (quint, 2H, H3, *J* = 7.5 Hz).^{13}C NMR (125 MHz, $CDCl_3$) δ 170.770 (C=O), 161.451 (N=C-N), 155.668-153.709 (C4', $^1J_{C-F}$ = 244.88 Hz) 155.297 (C8''), 151.641 (C6''), 148.852 (C4''), 141.689 (C2''), 134.621 (C1'), 122.017 (C6'), 121.028 (C3'), 119.430-119.375 (C2', $^3J_{C-F}$ = 6,88 Hz), 116.678-116.504 (C5', $^2J_{C-F}$ = 21.75 Hz), 106.906 (C9''), 53.152 (C6), 41.308 (N1''-C), 36.966 (C2), 29.705 (N5''-<u>C</u>H$_3$), 28.733 (N7''-<u>C</u>H$_3$), 28.067 (C4), 25.817 (C5), 24.294 (C3). HRMS (ESI) m/z calc. for $C_{21}H_{23}{}^{35}ClFN_9O_3$ [M+Na]$^+$ 526.1489 , found 526.1482; calc. for $C_{21}H_{23}{}^{37}ClFN_9O_3$ [M+Na]$^+$ 528.1459 , found 528.1483.

6-(5-((1,3-dimethyl-2,6-dioxo-1,2,3,6-tetrahydro-7H-purin-7-yl)methyl)-2H-tetrazol-2-yl)-N-(2-fluoro-4-iodophenyl)hexanamide (17). Prepared from acid (**8**) and 2-fluoro-4-iodo-bromoaniline following general procedure (d) to afford a off-white solid, yield: 44.3%. ^{1}H NMR (500 MHz, $CDCl_3$) δ ppm 8.08 (s, 1H, H3'), 7.75 (s, 1H, H2''), 7.42 (m, 3H, CO-N<u>H</u>, H5', H6'), 5.84 (s, 2H, N1''-C<u>H</u>$_2$), 4.61 (t, 2H, H6, *J* = 7.0Hz), 3.59 (s, 3H, N5''-C<u>H</u>$_3$), 3.39 (s, 3H, N7''-C<u>H</u>$_3$), 2.38 (t, 2H, H2, *J* = 7.5Hz), 2.04 (quint, 2H, H5, J_1 = 7.5Hz, J_2= 7.0Hz), 1.75 (quint, 2H, H3, J_1 = 8.0Hz, J_2= 7.5Hz), 1.39 (quint, 2H, H4, J_1 = 8.0Hz, J_2 = 7.5Hz). ^{13}C NMR (125 MHz, $CDCl_3$) δ 170.674 (C=O), 161.433 (N=<u>C</u>-N), 155.225 (C8''), 151.661 (C6''), 148.710 (C4''), 141.511 (C2', C2''), 133.836 (C3'), 133.806 (C1'), 124.033 (C5') 123.856 (C4'), 123.091 (C6'), 106.915 (C9''), 53.157 (C6), 41.165 (N1''-<u>C</u>H$_2$), 37.090 (C2), 29.848 (N5''-<u>C</u>H$_3$), 28.871 (N7''-<u>C</u>H$_3$), 28.008 (C5), 25.817 (C4), 24.322 (C3). HRMS (ESI) m/z calc. for $C_{21}H_{23}FIN_9O_3$ $[M+H]^+$ 618.0845, found 618.0848.

Phenyl 6-(5-((1,3-dimethyl-2,6-dioxo-1,2,3,6-tetrahydro-7H-purin-7-yl)methyl)-2H-tetrazol-2-yl)hexanoate (18). Prepared from acid (8) and phenol following general procedure (d) to afford a off-white solid, yield: 35.3%. ^{1}H NMR (500 MHz, $CDCl_3$) δ ppm 8.24 (s, 1H, H2''), 7.41 (t, 2H, H3', H5', *J* = 8.0Hz), 7.25 (t, 1H, H4', *J* =7.5Hz), 7.09 (d, 2H, H2', H6', *J* = 7.5Hz), 5.85 (s, 2H, N1''-C<u>H</u>$_2$), 4.67 (t, 2H, H6, *J* = 7.0Hz), 3.44 (s, 3H, N5''-C<u>H</u>$_3$), 3.17 (s, 3H, N7''-C<u>H</u>$_3$), 2.54 (t, 2H, H2, *J* = 7.5Hz), 1.92 (quint, 2H, H5, J_1 = 7.5Hz, J_2 = 7.0Hz), 1.64 (quint, 2H, H3, J_1 = 7.5Hz, J_2 = 7.0Hz), 1.30 (quint, 2H, H4, J_1 = 7.5Hz, J_2 = 7.0Hz). ^{13}C NMR (125 MHz, $CDCl_3$) δ 172.084 (C=O), 162.589 (N=C-N), 154.672 (C8''), 151.457-150.921 (C6''), 148.737 (C4''), 143.630 (C2''), 129.926 (C3', C5'), 126.215 (C4'), 122.246 (C2', C6'), 106.570 (C9''), 53.008 (C6), 41.648 (N1''-<u>C</u>H$_2$), 33.682 (C2), 29.951 (N5''-<u>C</u>H$_3$), 28.788 (N7''-<u>C</u>H$_3$), 27.934 (C4), 25.477 (C5), 24.047 (C3). HRMS (ESI) m/z calc. for $C_{21}H_{24}N_8O_4$ $[M+Na]^+$ 475.1813 , found 475.1876.

4-methoxyphenyl 6-(5-((1,3-dimethyl-2,6-dioxo-1,2,3,6-tetrahydro-7H-purin-7-yl)methyl)-2H-tetrazol-2-yl)hexanoate (19). Prepared from acid (**8**) and 4-methoxyphenol following general procedure (d) to afford a off-white solid, yield: 44.2% ^{1}H NMR (500 MHz, $CDCl_3$) δ ppm ^{1}H NMR (500 MHz, $CDCl_3$) δ ppm 7.76 (s, 1H, H2''), 6.99 (m, 2H, H2', H6'), 6.90 (m, 2H, H3', H5'), 5.87 (s, 2H, N1''-C<u>H</u>$_2$), 4.65 (t, 2H, H6, *J* = 7.0Hz), 3.82 (s, 3H, OC<u>H</u>$_3$), 3.62 (s, 3H, N5''-C<u>H</u>$_3$), 3.42 (s, 3H, N7''-C<u>H</u>$_3$), 2.56 (t, 2H, H2, *J* = 7.5Hz), 2.08 (quint, 2H, H5, *J* = 7.0Hz), 1.80 (quint, 2H, H4, *J* = 7.5Hz), 1.46 (quint, 2H, H3, *J* = 7.0Hz). ^{13}C NMR (125 MHz, $CDCl_3$) δ 172.083 (C=O), 161.455 (N=<u>C</u>-N), 157.277 (C4'), 155.222 (C8''), 151.676 (C6''), 148.681 (C4''), 144.081 (C2''), 141.461 (C1'), 122.235 (2C, C2',C6'), 114.477 (2C, C3', C5'), 106.919 (C9'')55.609 (O<u>C</u>H$_3$), 53.198 (C6), 41.130 (N1''-<u>C</u>H$_2$), 33.833 (C5), 29.826 (N5''-<u>C</u>H$_3$), 28.863 (N7''-<u>C</u>H$_3$), 27.990 (C2), 25.751 (C4), 24.082 (C3). HRMS (ESI) m/z calc. for $C_{22}H_{26}N_8O_5$ $[M+Na]^+$ 483.2099 , found 483.2080.

N-(3-chlorophenyl)-7-(5-((1,3-dimethyl-2,6-dioxo-1,2,3,6-tetrahydro-7H-purin-7-yl)methyl)-2H-tetrazol-2-yl)heptanamide (20). Prepared from acid (9) and 3-chloroaniline following general procedure (d) to afford a off-white solid off-white solid, yield: 50.2%. ^{1}H NMR (500 MHz, $CDCl_3$) δ ppm 7.79 (s, 1H, H2''), 7.68 (s, 2H, H2', CO-N<u>H</u>), 7.40 (d, 1H, H6', *J* = 8.0Hz), 7.24 (t, 1H, H5', *J* = 8.0Hz), 7.09 (d, 1H, H4', *J* = 7.5Hz), 5.87 (s, 2H, N1''-C<u>H</u>$_2$), 4.62 (t, 2H, H7, *J* = 7.0Hz), 3.62 (s, 3H, N5''-C<u>H</u>$_3$), 3.41 (s, 3H, N7''-C<u>H</u>$_3$), 2.32 (t, 2H, H2, *J* = 7.0Hz), 2.02 (quint, 2H, H6, *J* = 7.0Hz), 1.72 (quint, 2H, H3, *J* = 7.5Hz), 1.36 (m, 4H, H4, H5). ^{13}C NMR (125 MHz, $CDCl_3$) δ 171.124 (<u>C</u>=O), 161.432 (N=<u>C</u>-N), 155.301 (C8''), 151.630 (C6''), 148.783 (C4''), 141.607 (C2''), 139.234 (C1'), 134.635 (C3'), 129.979 (C5'), 124.164 (C4'), 119.769 (C2'), 117.586 (C6'), 106.916 (C9''), 53.244 (C7), 41.254 (N1''-C), 36.930 (C2), 29.886 (N5''-<u>C</u>H$_3$), 28.821 (N7''-<u>C</u>H$_3$), 28.055 (C4), 27.987 (C6), 25.666 (C5), 24.759 (C3). HRMS (ESI) m/z calc. for $C_{22}H_{26}{}^{35}ClN_9O_5$ $[M+Na]^+$ 522.1739 , found 522.1722; calc. for $C_{22}H_{26}{}^{37}ClN_9O_5$ $[M+Na]^+$ 524.1719 , found 524.1725.

N-(4-chlorophenyl)-7-(5-((1,3-dimethyl-2,6-dioxo-1,2,3,6-tetrahydro-7H-purin-7-yl)methyl)-2H-tetrazol-2-yl)heptanamide (21). Prepared from acid (**9**) and 4-chloroaniline following general procedure (d) to afford a off-white solid off-white solid, yield: 46.2%. ^{1}H NMR (500 MHz, $CDCl_3$) δ ppm 7.78 (s, 1H, H2''), 7.61 (s, 1H, CO-N<u>H</u>), 7.52 (d, 2H, H2', H6', *J* = 9.0Hz), 7.29 (t, 2H, H3', H5', *J* = 5.0 Hz), 5.86 (s, 2H, N1''-C<u>H</u>$_2$), 4.62 (t, 2H, H7, *J* = 7.0Hz), 3.62 (s, 3H, N5''-C<u>H</u>$_3$), 3.41 (s, 3H, N7''-C<u>H</u>$_3$), 2.32 (t, 2H, H2, *J* = 7.0Hz), 2.02 (quint, 2H, H6, *J* = 7.0Hz), 1.72 (t, 2H, H3, *J* = 7.5Hz), 1.40 (m, 4H, H4, H5). ^{13}C NMR (125 MHz, $CDCl_3$) δ 171.042 (C=O), 161.429 (N=<u>C</u>-N), 155.289 (C8''), 151.631 (C6''), 148.771 (C4''), 141.600 (C2''), 136.650 (C1'), 128.991 (C2', C6'), 128.991 (C3', C5'), 120.936 (C4'), 106.915 (C9''), 53.254 (C7), 41.247 (N1''-<u>C</u>H$_2$), 36.950 (C2), 29.883 (N5''-<u>C</u>H$_3$), 28.822 (N7''-<u>C</u>H$_3$), 28.048 (C4), 28.029 (C6), 25.686 (C5), 24.805 (C3). HRMS (ESI) m/z calc. for $C_{22}H_{26}{}^{35}ClN_9O_5$ $[M+Na]^+$ 522.1739 , found 522.1718; calc. for $C_{22}H_{26}{}^{37}ClN_9O_5$ $[M+Na]^+$ 524.1719, found 524.1724.

N-(4-bromophenyl)-7-(5-((1,3-dimethyl-2,6-dioxo-1,2,3,6-tetrahydro-7H-purin-7-yl)methyl)-2H-tetrazol-2-yl)heptanamide (22). Prepared from acid (9) and 4-bromoaniline following general procedure (d) to afford a off-white solid off-white solid, yield: 40.6%. ^{1}H NMR (500 MHz, $CDCl_3$) δ ppm 7.76 (s, 1H, H2''), 7.57 (s, 1H, CO-N<u>H</u>), 7.43 (m, 4H, H2', H3', H5', H6'), 5.84 (s, 2H, N1''-C<u>H</u>$_2$), 4.60 (t, 2H, H7, *J* = 7.0Hz), 3.60 (s, 3H, N5''-C<u>H</u>$_3$), 3.39 (s, 3H, N7''-C<u>H</u>$_3$), 2.30 (t, 2H, H2, *J* = 7.5Hz), 2.00 (quint, 2H, H6, *J* = 7.0Hz), 1.70 (quint, 2H, H3, *J* = 7.0Hz), 1.34 (m, 4H, H4, H5). ^{13}C NMR (125 MHz, $CDCl_3$) δ 170.996 (C=O), 161.425 (N=C-N), 155.298 (C8''), 151.632 (C6''), 148.776 (C4''), 141.582 (C2''), 137.133 (C1'), 131.957 (C2', C6'), 121.237 (C3', C5'), 116.680 (C4'), 106.914 (C9''), 53.245 (C7), 41.247 (N1''- <u>C</u>H$_2$), 36.969 (C2), 29.883 (N5''-<u>C</u>H$_3$), 28.812 (N7''-<u>C</u>H$_3$), 28.048 (C6), 27.999 (C3), 25.661 (C5), 24.763 (C4). HRMS (ESI) m/z calc. for $C_{22}H_{26}{}^{79}BrN_9O_5$ $[M+Na]^+$ 566.1234 , found 566.1231 ; calc. for $C_{22}H_{26}{}^{81}BrN_9O_5$ $[M+Na]^+$ 568.1214 , found 568.1207.

N-(3-chloro-4-fluorophenyl)-7-(5-((1,3-dimethyl-2,6-dioxo-1,2,3,6-tetrahydro-7H-purin-7-yl)methyl)-2H-tetrazol-2-yl)heptanamide (23). Prepared from acid (**9**) and 3-chloro-4-fluoroaniline following general procedure (d) to afford a off-white solid off-white solid, yield: 60.9%. ^{1}H NMR (500 MHz, $CDCl_3$) δ ppm 7.77 (s, 1H, H2'') 7.73 (d, 1H, H2', J = 3.5Hz), 7.65 (s, 1H, CO-NH) 7.35 (d, 1H, H5', *J* = 8.5Hz), 7.07 (t, 1H, H6', *J* = 8.5Hz), 5.85 (s, 2H, N1''-CH_2), 4.61 (t, 2H, H7, *J* = 7.0Hz), 3.60 (s, 3H, N5''-CH_3), 3.39 (s, 3H, N7''-CH_3), 2,29 (t, 2H, H2, *J* = 7.5Hz), 2.00 (m, 2H, H6), 1.70 (m, 2H, H3), 1.38 (m, 2H, H5), 1.31 (m, 2H,H4). ^{13}C NMR (125 MHz, $CDCl_3$) δ 171.042 (C=O), 161.436 (N=C-N), 155.318 (C8''), 153.655 (C4'), 151.619 (C6''), 148.805 (C4''), 141.625 (C2''), 134.697 (C1'), 121.955 (C6'), 119.302 (C3'), 116.654 (C2'), 116.479(C5'), 106.910 (C9''), 53.229 (C7), 41.276 (N1''-CH_2), 36.755 (C2), 29.895(N5''-CH_3), 28.787 (N7''-CH_3), 28.060 (C4), 27.391 (C6), 25.604 (C5), 24.717 (C3). HRMS (ESI) m/z calc. for $C_{22}H_{25}{}^{35}ClFN_9O_5$ $[M+Na]^+$ 540.1645 , found 540.1605 ; calc. for $C_{22}H_{25}{}^{37}ClFN_9O_5$ $[M+Na]^+$ 542.1616 , found 542.1601.

4-methoxyphenyl 7-(5-((1,3-dimethyl-2,6-dioxo-1,2,3,6-tetrahydro-7H-purin-7-yl)methyl)-2H-tetrazol-2-yl)heptanoate (24). Prepared from acid (**9**) and 4-methoxyphenol following general procedure (d) to afford a off-white solid off-white, yield: 48.7%. ^{1}H NMR (500 MHz, $CDCl_3$) δ ppm 7.76 (s, 1H, H2''), 7.01 (d, 2H, H2', H6', *J* = 9.0Hz), 6.90 (d, 2H, H3', H5', *J* = 9.0Hz), 5.87 (s, 2H, N1''-CH_2), 4.63 (t, 2H, H7, *J* = 7.0Hz), 3.82 (s, 3H, OCH_3), 3.62 (s, 3H, N5''-CH_3), 3.42 (s, 3H, N7''-CH_3), 2.55 (t, 2H, H2, *J* = 7.5Hz), 2.05 (quint, 2H, H6, *J* = 7.5Hz), 1.75 (quint, 2H, H3, *J* = 7.5Hz), 1.47 (m, 2H, H5), 1.41 (m, 2H, H4). ^{13}C NMR (125 MHz, $CDCl_3$) δ 172.360 (C=O), 157.239 (C4'), 155.222 (C8''), 151.676 (C6''), 144.135 (C2'') 141.453 (C1'), 122.268 (C2', C6'), 114.464 (C3', C5'), 106.923 (C9''), 55.606 (OCH_3), 53.338 (C7), 41.130 (N1''-CH_2), 34.016 (C2), 29.829 (N5''-CH_3), 28.977 (N7''-CH_3), 28.253 (C4), 27.992 (C6), 25.958 (C5), 24.536 (C3). HRMS (ESI) m/z calc. for $C_{23}H_{28}N_8O_5$ $[M+Na]^+$ 497.2255 , found 497.2240.

N-(4-bromophenyl)-7-(5-((1,3-dimethyl-2,6-dioxo-1,2,3,6-tetrahydro-7H-purin-7-yl)methyl)-1H-tetrazol-1-yl)heptanamide (25). Prepared from acid (**10**) and 4-bromoaniline following general procedure (d) to afford a off-white solid off-white, yield: 64.5%. ^{1}H NMR (500 MHz, $CDCl_3$) δ ppm 7.91 (s, 1H, H2''), 7.43 (m, 5H, CO-NH, H2', H3', H5', H6', *J* = 7.5Hz), 5.88 (s, 2H, N1''-CH_2), 4.55 (t, 2H, H7, *J* = 7.0Hz), 3.62 (s, 3H, N5''-CH_3), 3.39 (s, 3H, N7''-CH_3), 2,34 (t, 2H, H2, *J* = 7.0Hz), 1.94 (quint, 2H, H6, *J* = 7.0Hz), 1.73 (quint, 2H, H3, *J* = 7.5Hz), 1.40 (m, 4H, H4, H5). ^{13}C NMR (125 MHz, $CDCl_3$) δ 170.965 (C=O), 155.606 (N=C-N), 151.426 (C8''), 150.251 (C6''), 148.813 (C4''), 142.139 (C2''), 136.987 (C1'), 131.948 (C2', C6'), 121.206 (C3', C5'), 116.762 (C4'), 106.217 (C9''), 47.639 (C7), 37.908 (N1''-C), 37.068 (C2), 29.990 (N5''-CH_3), 29.713 (N7''-CH_3), 29.565 (C6), 28.059 (C3), 25.748 (C5), 24.743 (C4). HRMS (ESI) m/z calc. for $C_{22}H_{26}{}^{79}BrN_9O_5$ $[M+Na]^+$ 566.1234 , found 566.1241 ; calc. for $C_{22}H_{26}{}^{81}BrN_9O_5$ $[M+Na]^+$ 568.1214 , found 568.1228.

N-(3-chloro-4-fluorophenyl)-7-(5-((1,3-dimethyl-2,6-dioxo-1,2,3,6-tetrahydro-7H-purin-7-yl)methyl)-1H-tetrazol-1-yl)heptanamide (26). Prepared from acid (**10**) and 3-chloro-4-fluoroaniline following general procedure (d) to afford a off-white solid, yield: 72.4%. ^{1}H NMR (500 MHz, $CDCl_3$) δ ppm 7.91 (s, 1H, H2''), 7.74 (m, 1H, CO-NH), 7.56 (brs, 1H, H2'), 7.34 (m, 1H, H5'), 7.08 (t, 1H, H6', *J* = 8.5Hz), 5.89 (s, 2H, N1''-CH_2), 4.55 (t, 2H, H6, *J* = 7.0Hz), 3.61 (s, 3H, N5''-CH_3), 3.39 (s, 3H, N7''-CH_3), 2,33 (t, 2H, H2, *J* = 7.5Hz), 1.92 (quint, 2H, H6, *J* = 7.0Hz), 1.36 (m, 6H, H3, H4, H5). ^{13}C NMR (125 MHz, $CDCl_3$) δ 171.022 (C=O), 155.608 (N=C-N), 151.433 (C8''), 150.292 (C6''), 148.828 (C4''), 142.165 (C2''), 134.556 (C1'), 121.934 (C6'), 119.343 (C2'), 116.664-116.489 (C3'), 116.486 (C5'), 106.223 (C9''), 47.650 (C7), 37.947 (N1''-CH_2), 36.882 (C2), 29.977 (N5''-CH_3), 29.542 (N7''-CH_3), 28.136 (C6), 28.059 (C3), 25.738 (C5), 24.741 (C4). HRMS (ESI) m/z calc. for $C_{22}H_{25}{}^{35}ClFN_9O_5$ $[M+H]^+$ 518.1826 , found 518.1997 ; calc. for $C_{22}H_{25}{}^{37}ClFN_9O_5$ $[M+H]^+$ 520.1796 , found 520.1942; calc. for $[M+Na]^+$ 540.1645 , found 540.11803 ; calc. for $C_{22}H_{25}{}^{37}ClFN_9O_5$ $[M+Na]^+$ 542.1616, found 542.1795.

7-(5-((1,3-dimethyl-2,6-dioxo-1,2,3,6-tetrahydro-7H-purin-7-yl)methyl)-1H-tetrazol-1-yl)-N-(2-fluoro-4-iodophenyl)heptanamide (27). Prepared from acid (**10**) and 3-chloro-4-fluoroaniline following general procedure (d) to afford a off-white solid, yield: 68.3%. ^{1}H NMR (500 MHz, $CDCl_3$) δ 8.08 (t, 1H, H3', *J* =7.5Hz), 7.89 (s, 1H, H2''), 7.41 (m, 2H, H5', H6'), 7.38 (s, 1H, CO-NH), 5.86 (s, 2H, N1''-CH_2), 4.546 (t, 2H, H7, *J* = 7.0Hz), 3.60 (s, 3H, N5''-CH_3), 3.37 (s, 3H, N7''-CH_3), 2.39 (t, 2H, H2, *J* = 7.5Hz), 1.92 (quint, 2H, H6, *J* = 7.5Hz), 1.72 (quint, 2H, H3, *J* = 7.5Hz), 1.40 (m, 4H, H4, H5). ^{13}C NMR (125 MHz, $CDCl_3$) δ 170.954 (C=O), 155.593 (N=C-N), 151.381 (C8''), 150.168 (C6''), 148.788 (C4''), 142.086 (C2''), 133.785 (C2'), 123.991 (C5') 123.815 (C1'), 122.967 (C3',C6'), 106.168 (C9''), 47.639 (C7), 37.831 (N1''-CH_2), 37.197 (C2), 29.960 (N5''-CH_3), 29.702 (N7''-CH_3), 29.626 (C6), 28.185 (C3), 25.876 (C5), 24.700 (C4). HRMS (ESI) m/z calc. for $C_{22}H_{25}FIN_9O_5$ $[M+Na]^+$ 632.1001 , found 632.1000.

2D SPECTRA

Ester 3:

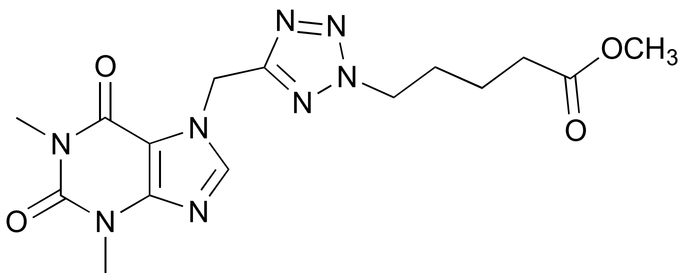

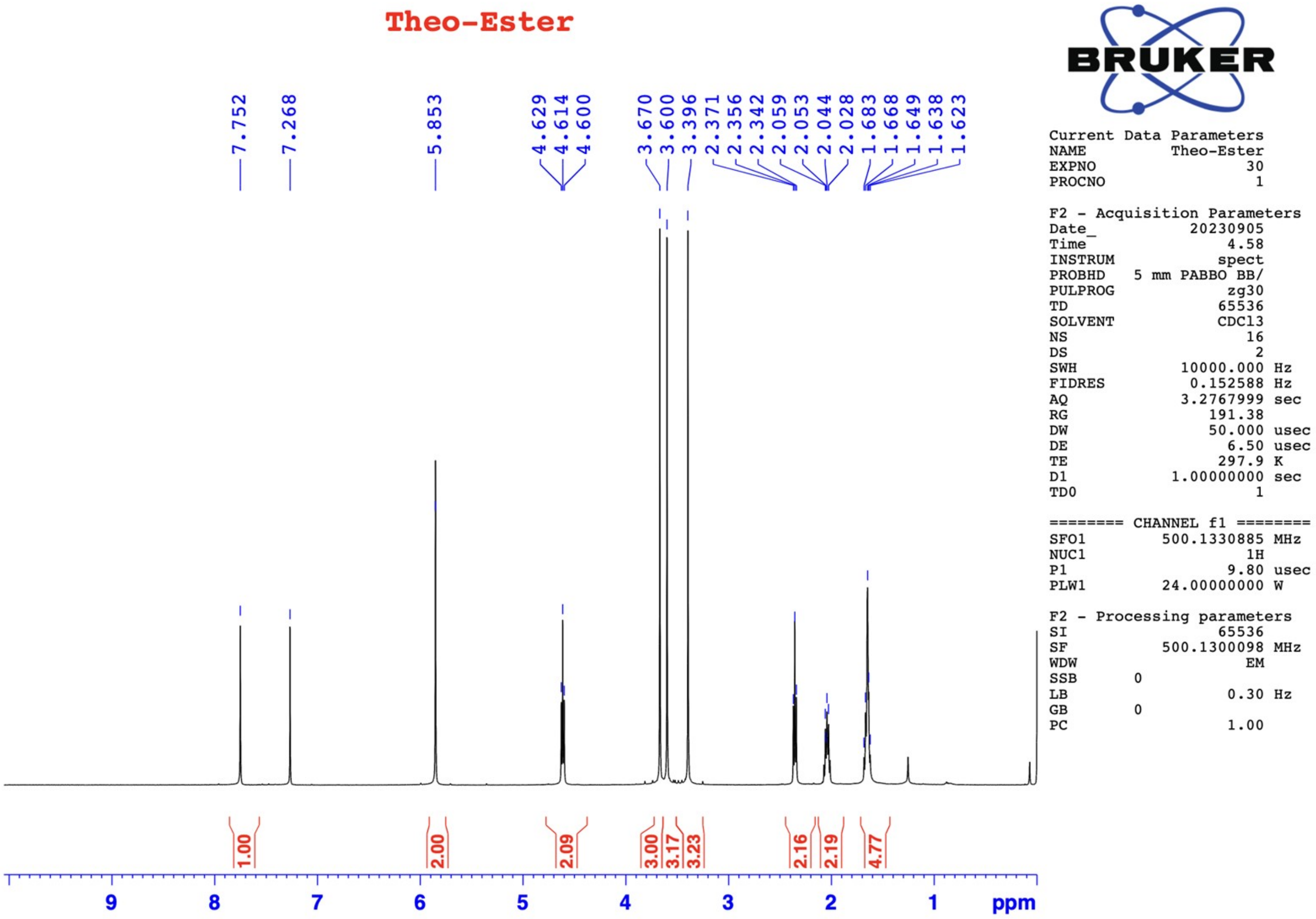

No interaction between N-C<u>H</u>$_2$-tetrazole and N-C<u>H</u>$_3$

No interaction between N-C<u>H</u>$_2$-tetrazole and N-C<u>H</u>$_2$-…-COOMe

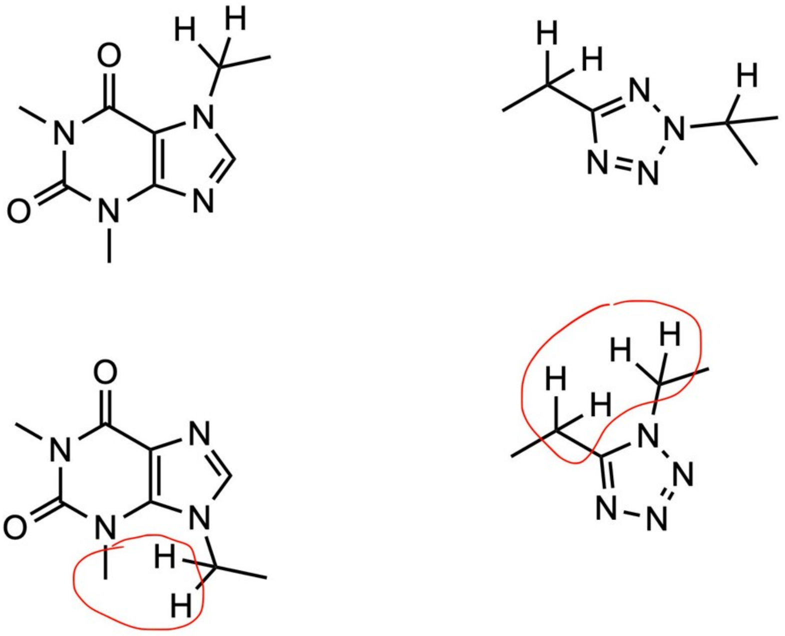

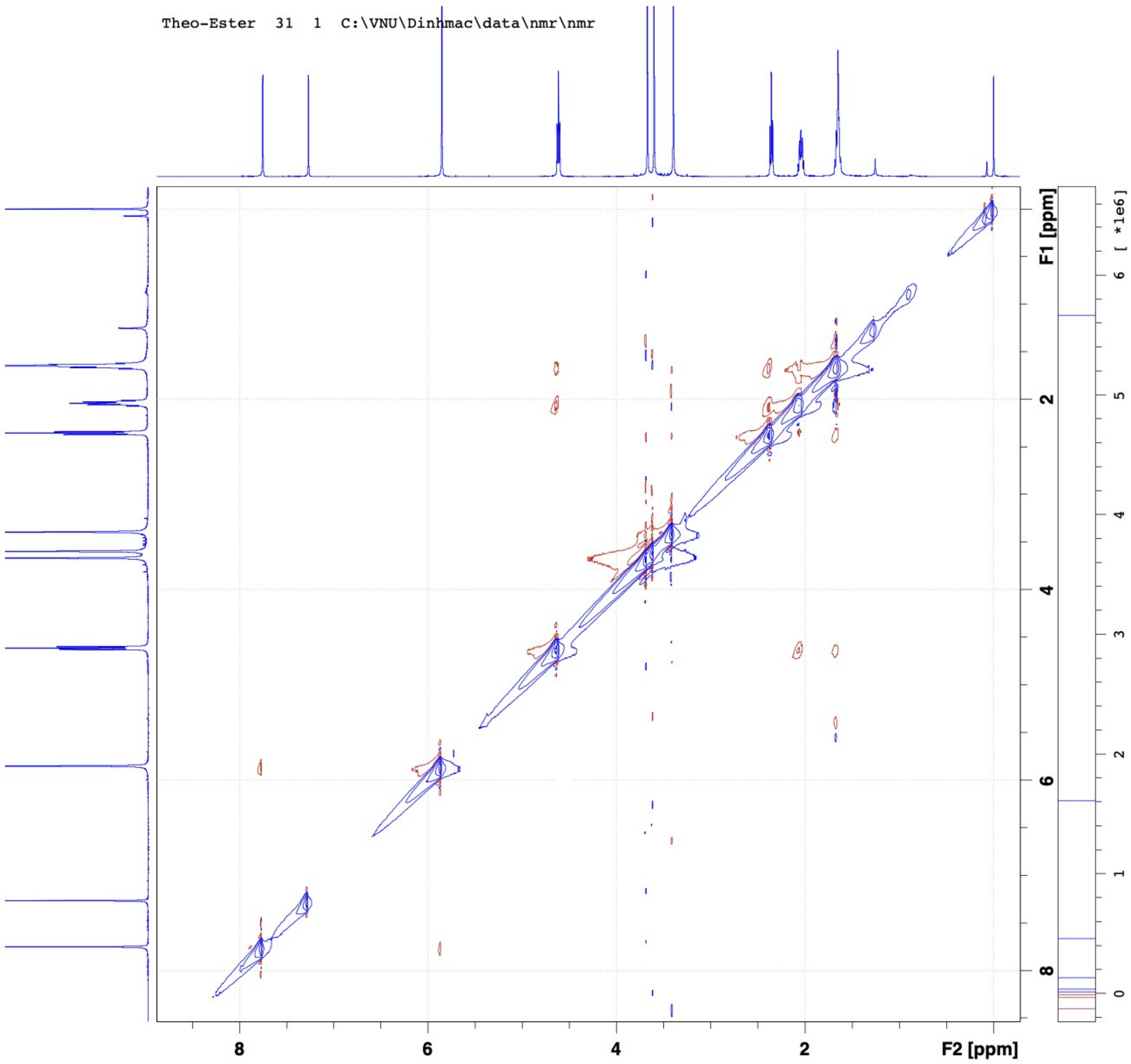
Theo-Ester 31 1 C:\VNU\Dinhmac\data\nmr\nmr
F1 [ppm]
F2 [ppm]
[*1e6]

Ester 4

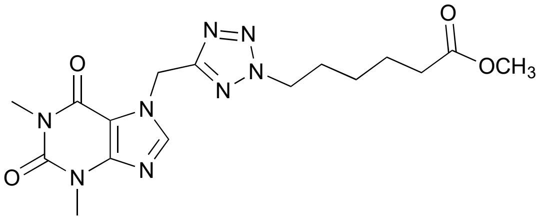

N=N
N
N
O
OCH3
O
N
N
N
O
N

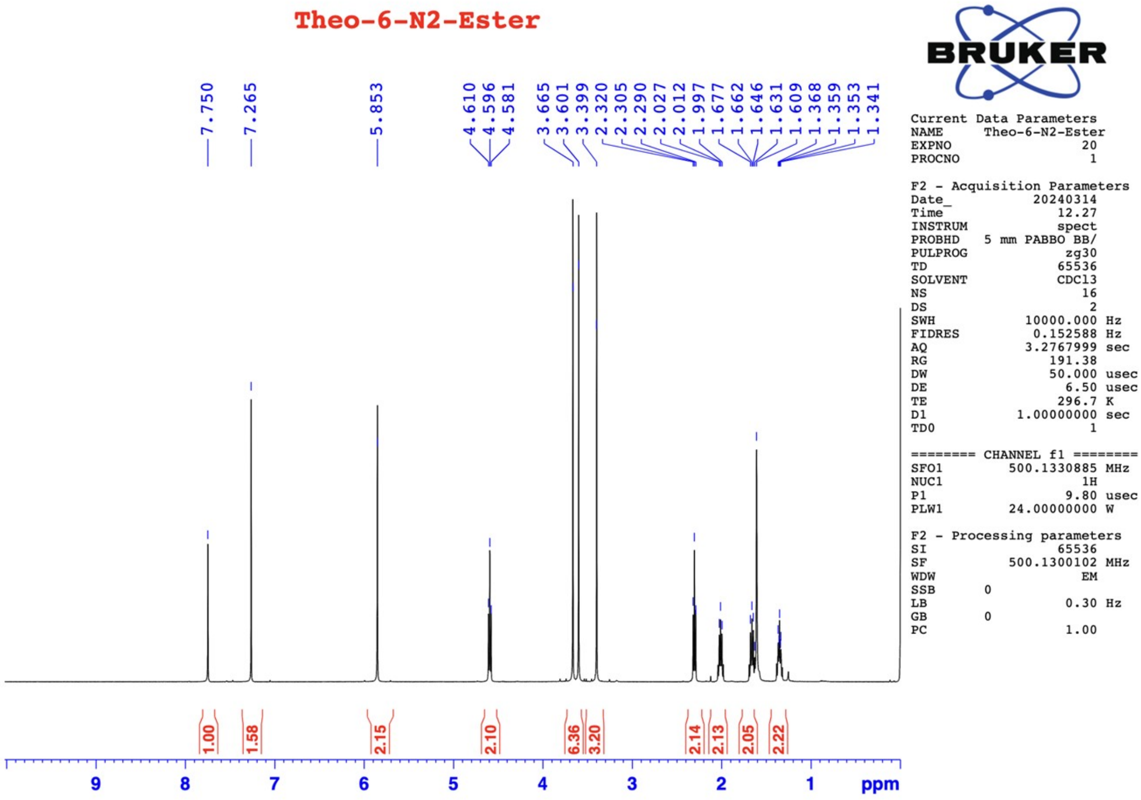

Theo-6-N2-Ester
BRUKER
7.750
7.265
5.853
4.610
4.596
4.581
3.665
3.601
3.399
2.320
2.305
2.290
2.027
2.012
1.997
1.677
1.662
1.646
1.631
1.609
1.368
1.359
1.353
1.341
1.00
1.58
2.15
2.10
6.36
3.20
2.14
2.13
2.05
2.22
9
8
7
6
5
4
3
2
1
ppm
Current Data Parameters
NAME Theo-6-N2-Ester
EXPNO 20
PROCNO 1
F2 - Acquisition Parameters
Date_ 20240314
Time 12.27
INSTRUM spect
PROBHD 5 mm PABBO BB/
PULPROG zg30
TD 65536
SOLVENT CDCl3
NS 16
DS 2
SWH 10000.000 Hz
FIDRES 0.152588 Hz
AQ 3.2767999 sec
RG 191.38
DW 50.000 usec
DE 6.50 usec
TE 296.7 K
D1 1.00000000 sec
TD0 1
======== CHANNEL f1 ========
SFO1 500.1330885 MHz
NUC1 1H
P1 9.80 usec
PLW1 24.00000000 W
F2 - Processing parameters
SI 65536
SF 500.1300102 MHz
WDW EM
SSB 0
LB 0.30 Hz
GB 0
PC 1.00

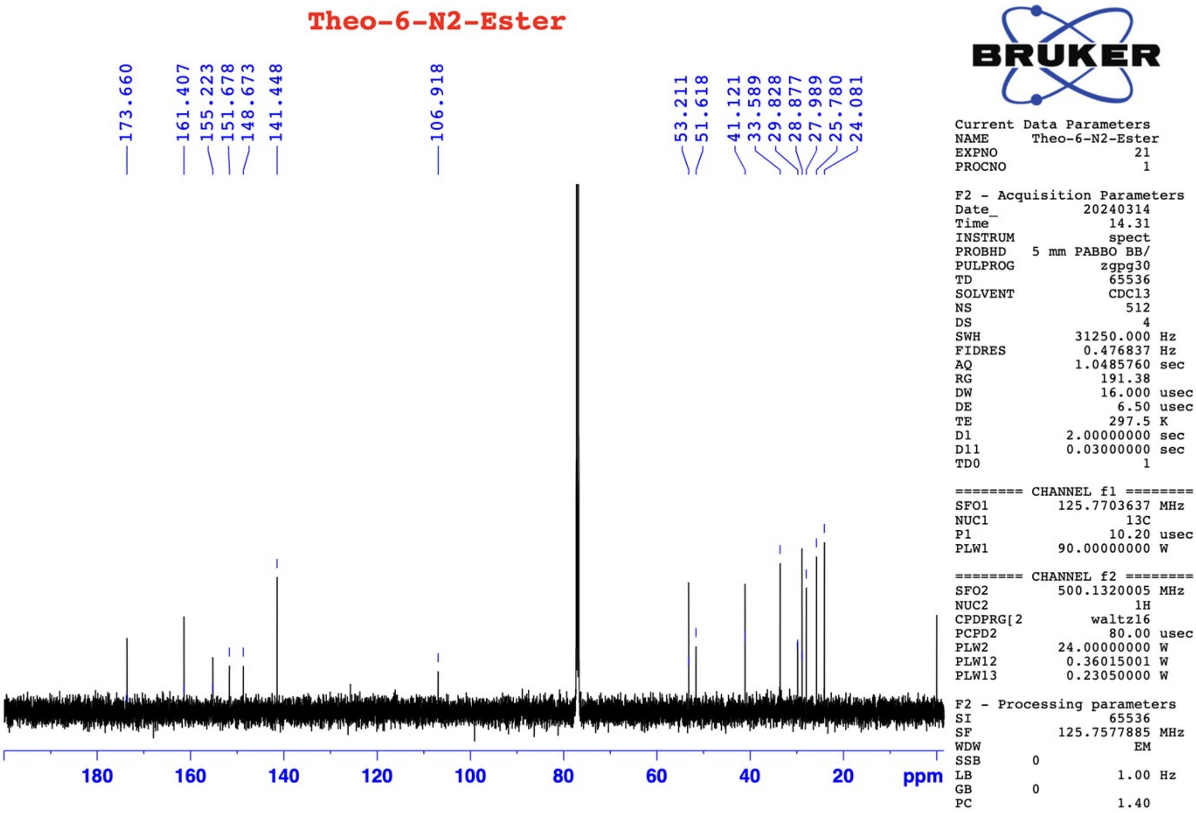

Theo-6-N2-Ester
BRUKER
173.660
161.407
155.223
151.678
148.673
141.448
106.918
53.211
51.618
41.121
33.589
29.828
28.877
27.989
25.780
24.081
180
160
140
120
100
80
60
40
20
ppm
Current Data Parameters
NAME Theo-6-N2-Ester
EXPNO 21
PROCNO 1
F2 - Acquisition Parameters
Date_ 20240314
Time 14.31
INSTRUM spect
PROBHD 5 mm PABBO BB/
PULPROG zgpg30
TD 65536
SOLVENT CDCl3
NS 512
DS 4
SWH 31250.000 Hz
FIDRES 0.476837 Hz
AQ 1.0485760 sec
RG 191.38
DW 16.000 usec
DE 6.50 usec
TE 297.5 K
D1 2.00000000 sec
D11 0.03000000 sec
TD0 1
======== CHANNEL f1 ========
SFO1 125.7703637 MHz
NUC1 13C
P1 10.20 usec
PLW1 90.00000000 W
======== CHANNEL f2 ========
SFO2 500.1320005 MHz
NUC2 1H
CPDPRG[2 waltz16
PCPD2 80.00 usec
PLW2 24.00000000 W
PLW12 0.36015001 W
PLW13 0.23050000 W
F2 - Processing parameters
SI 65536
SF 125.7577885 MHz
WDW EM
SSB 0
LB 1.00 Hz
GB 0
PC 1.40

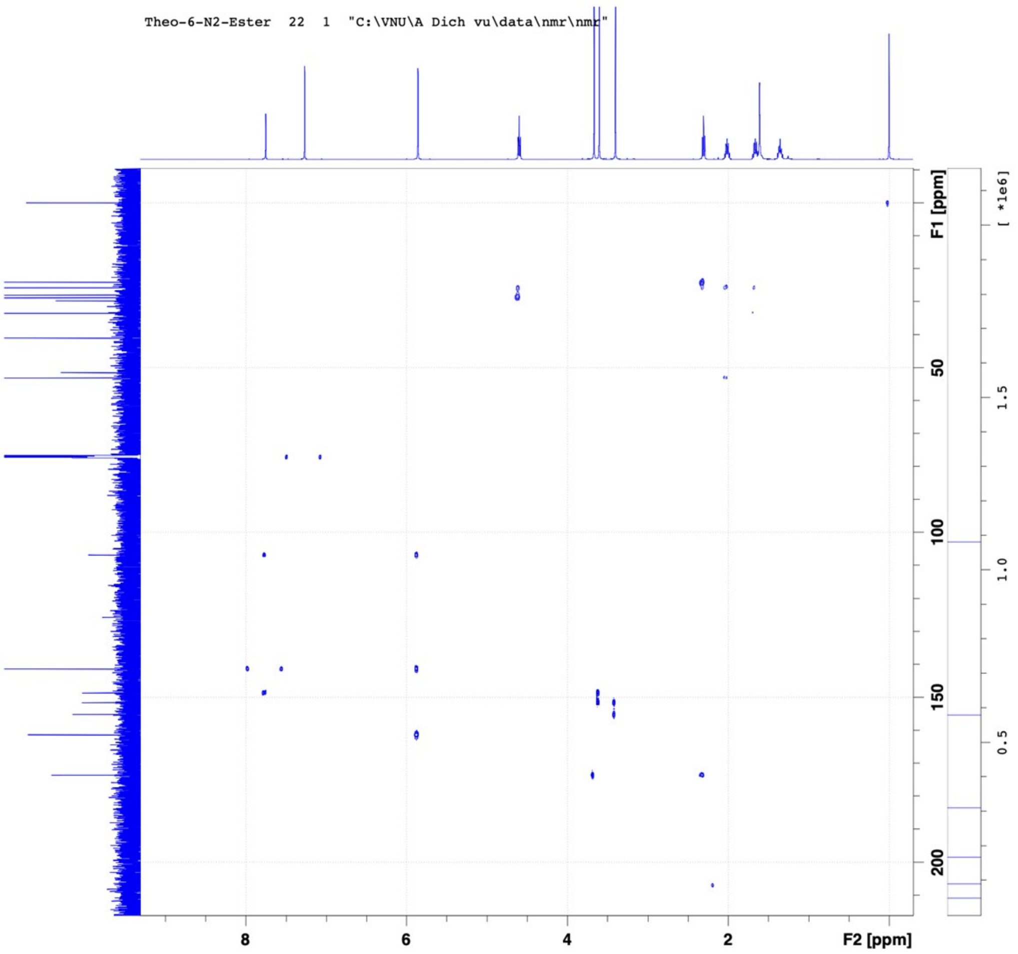
Theo-6-N2-Ester 22 1 "C:\VNU\A Dich vu\data\nmr\nmr"
F1 [ppm]
F2 [ppm]
[*1e6]

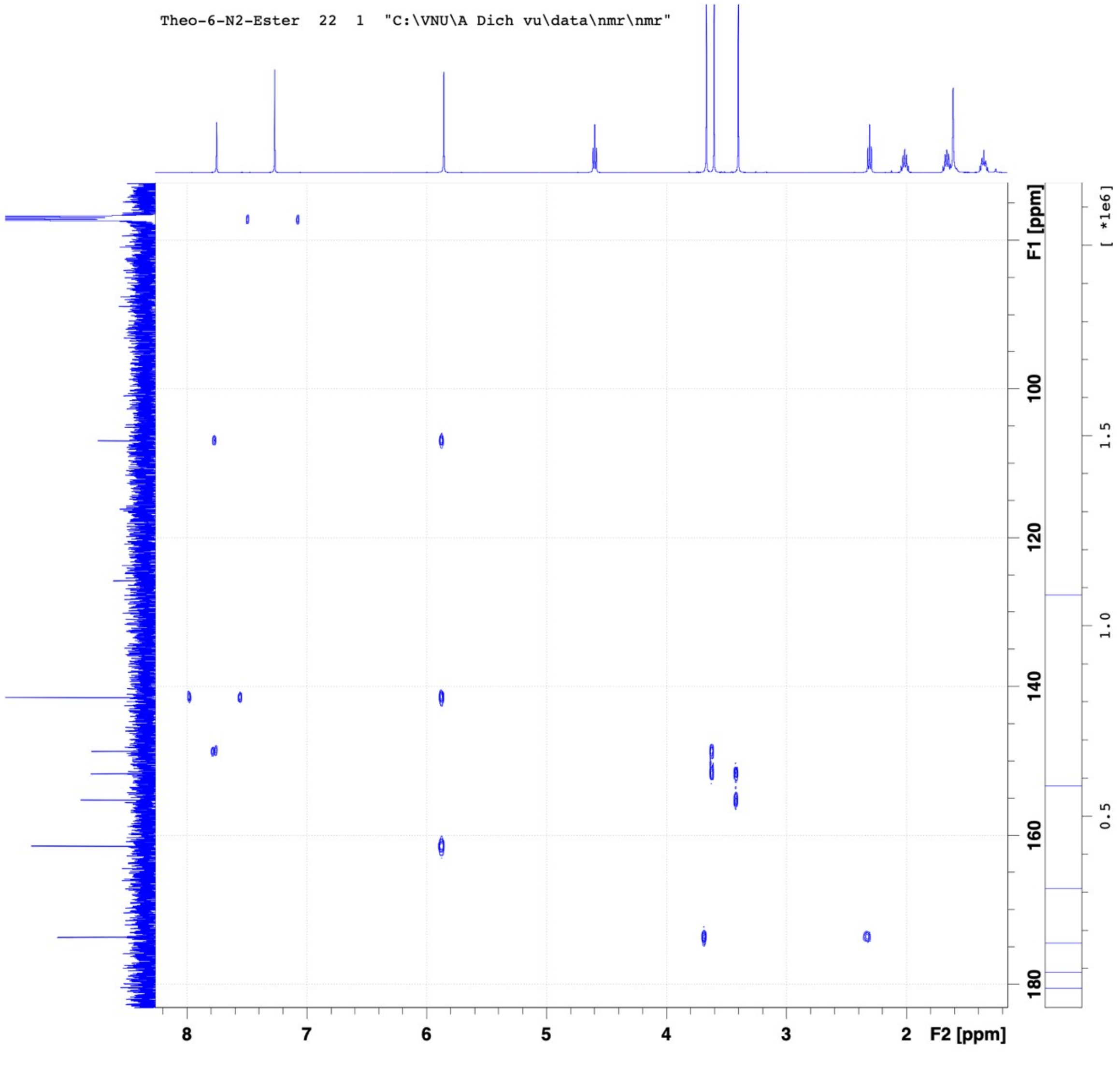
Theo-6-N2-Ester 22 1 "C:\VNU\A Dich vu\data\nmr\nmr"
F1 [ppm]
F2 [ppm]
[*1e6]

Ester 5:

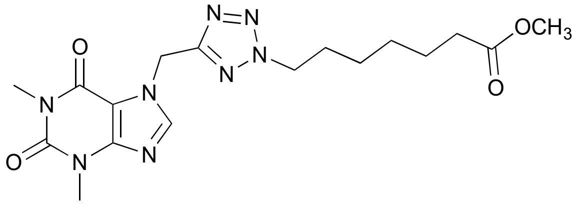

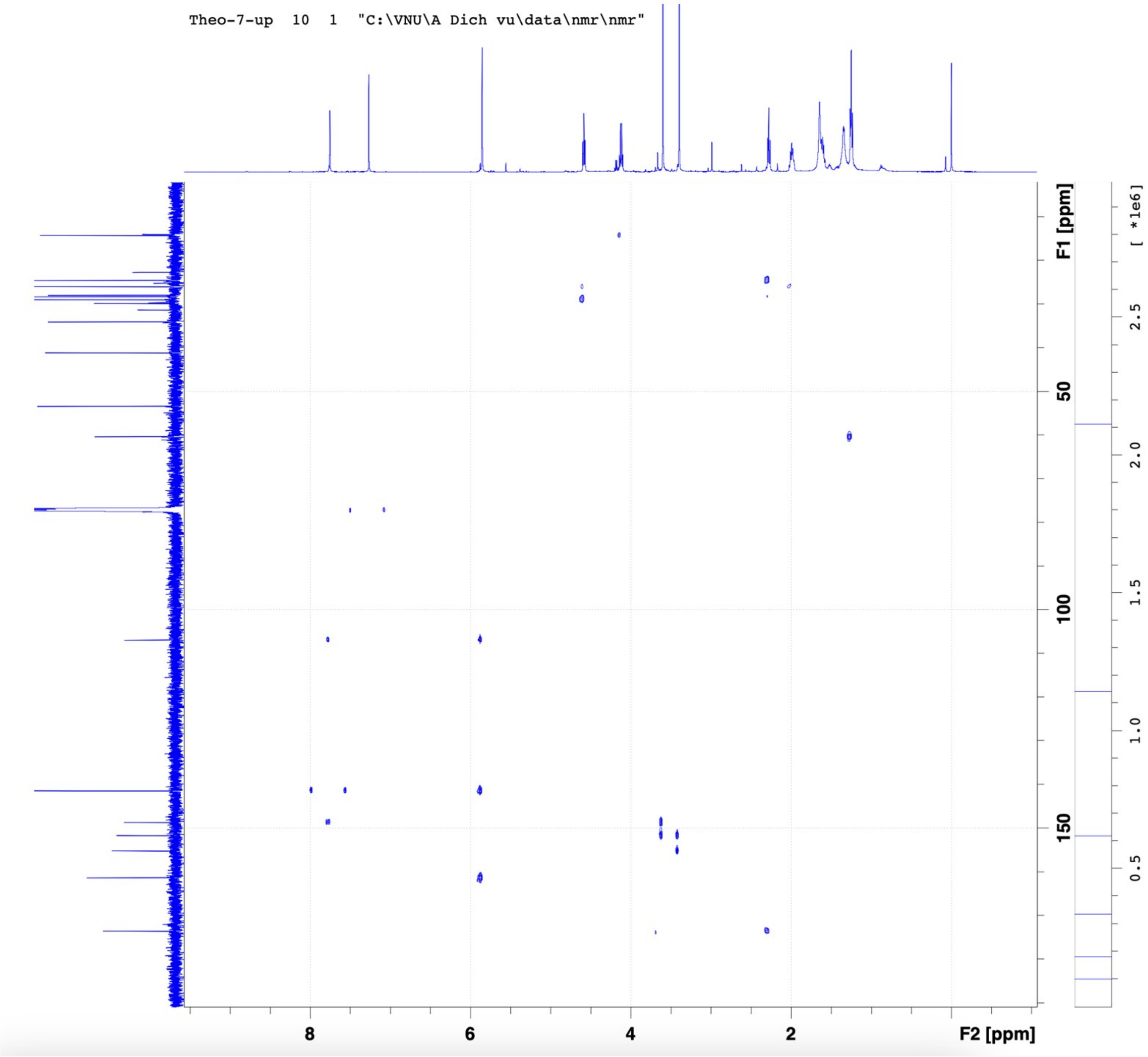
Theo-7-up 10 1 "C:\VNU\A Dich vu\data\nmr\nmr"
F1 [ppm]
50
100
150
[*1e6]
2.5
2.0
1.5
1.0
0.5
8
6
4
2
F2 [ppm]

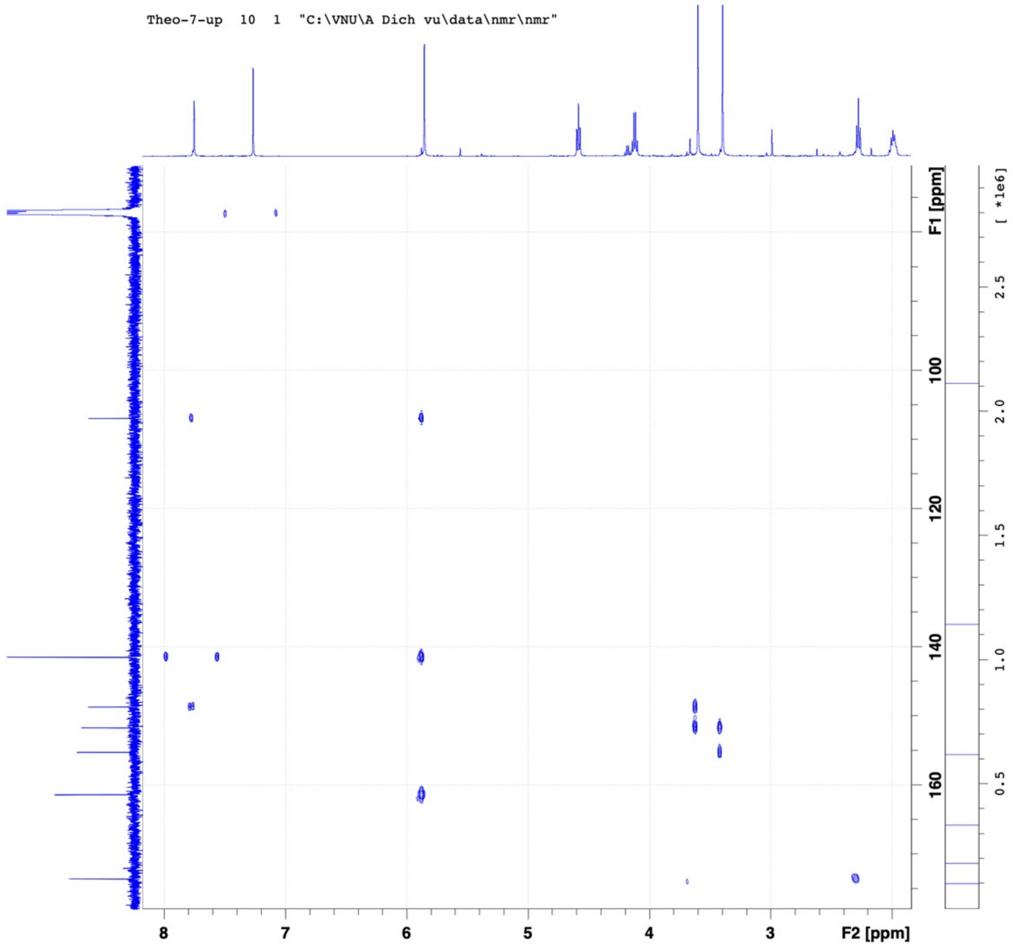

Theo-7-up 10 1 "C:\VNU\A Dich vu\data\nmr\nmr"
F1 [ppm]
F2 [ppm]
[*1e6]

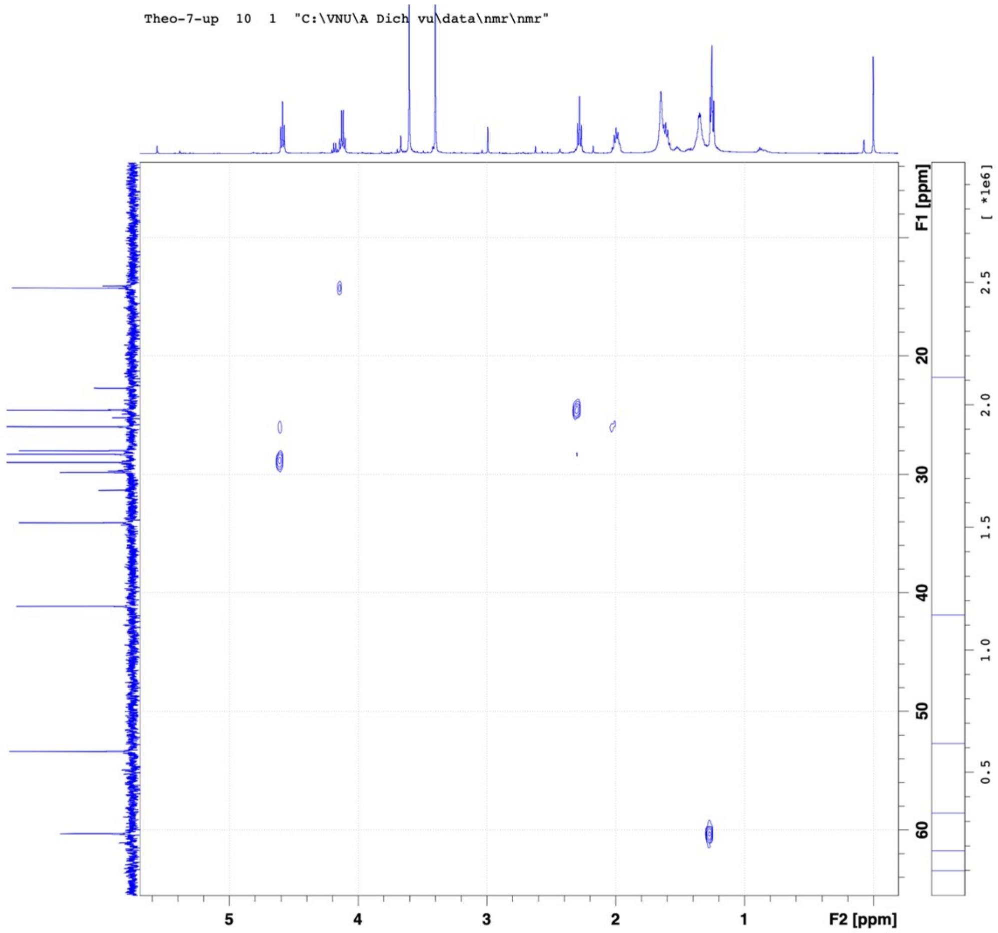

Theo-7-up 10 1 "C:\VNU\A Dich vu\data\nmr\nmr"
F1 [ppm]
F2 [ppm]
[*1e6]

ALL ^{1}H-NMR, ^{13}C-NMR & MS SPECTRA OF THE FINAL COMPOUNDS (11-27)

^{1}H-NMR of compound 11

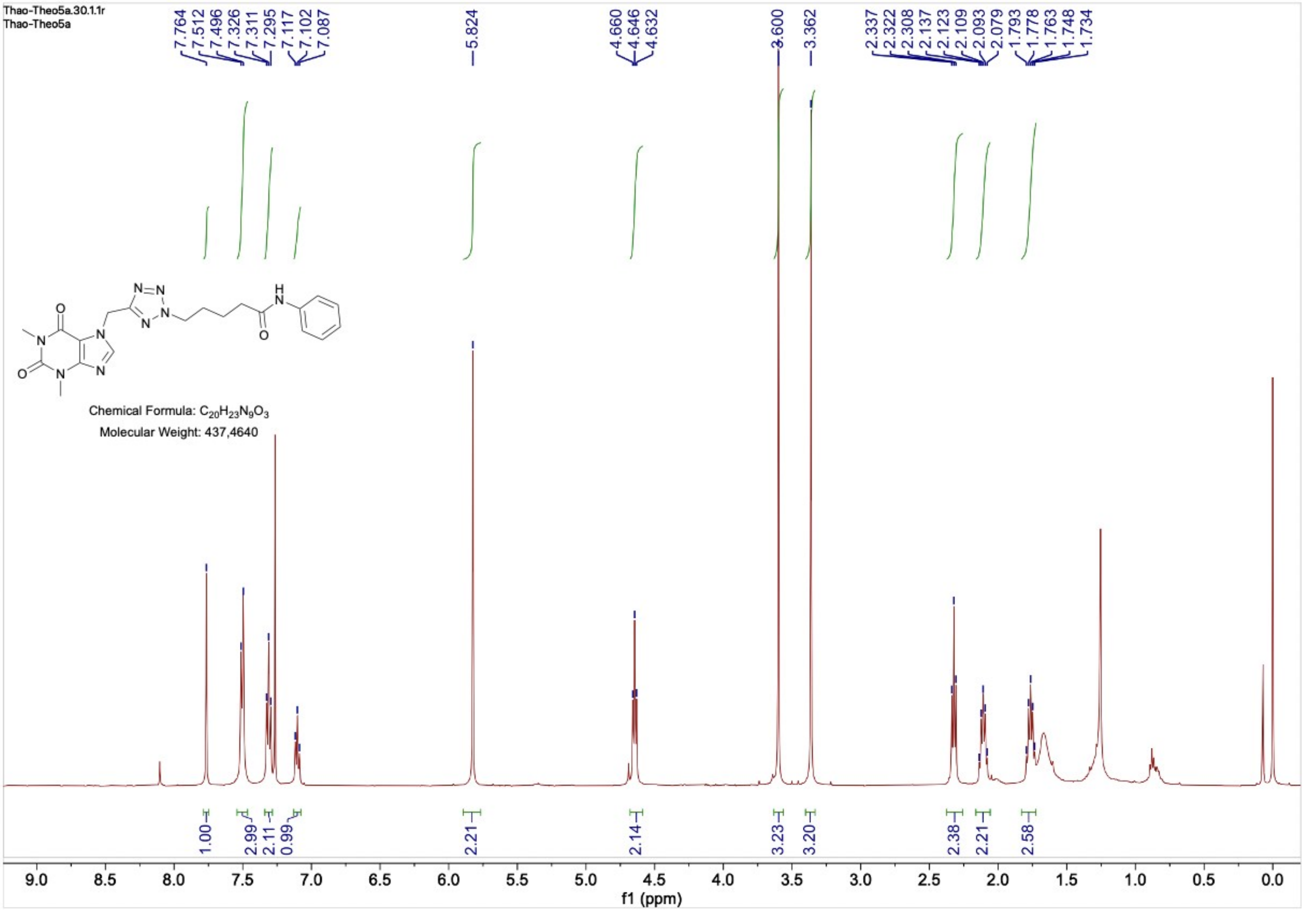

^{13}C-NMR of compound 11

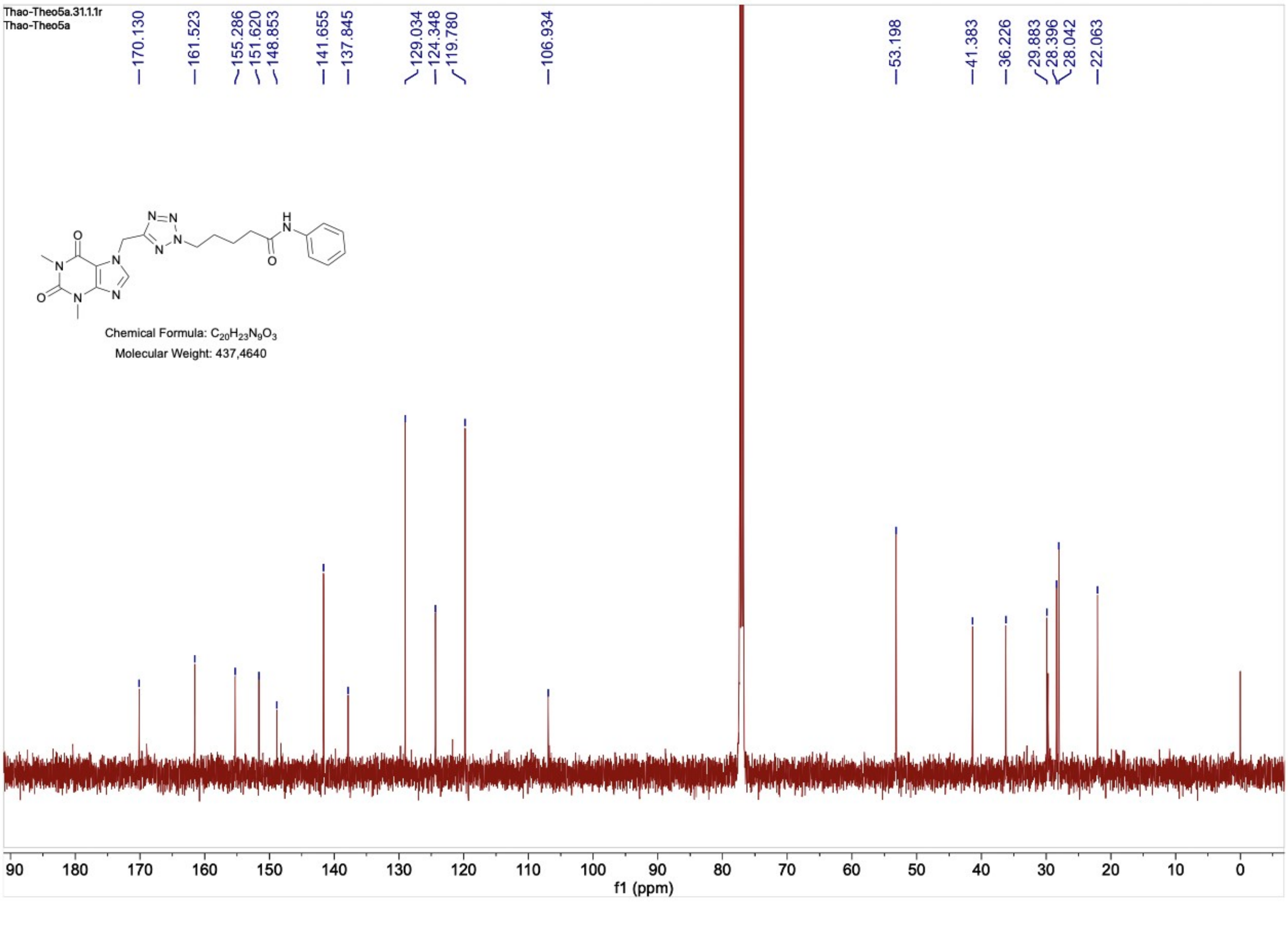

HR-MS of compound 11

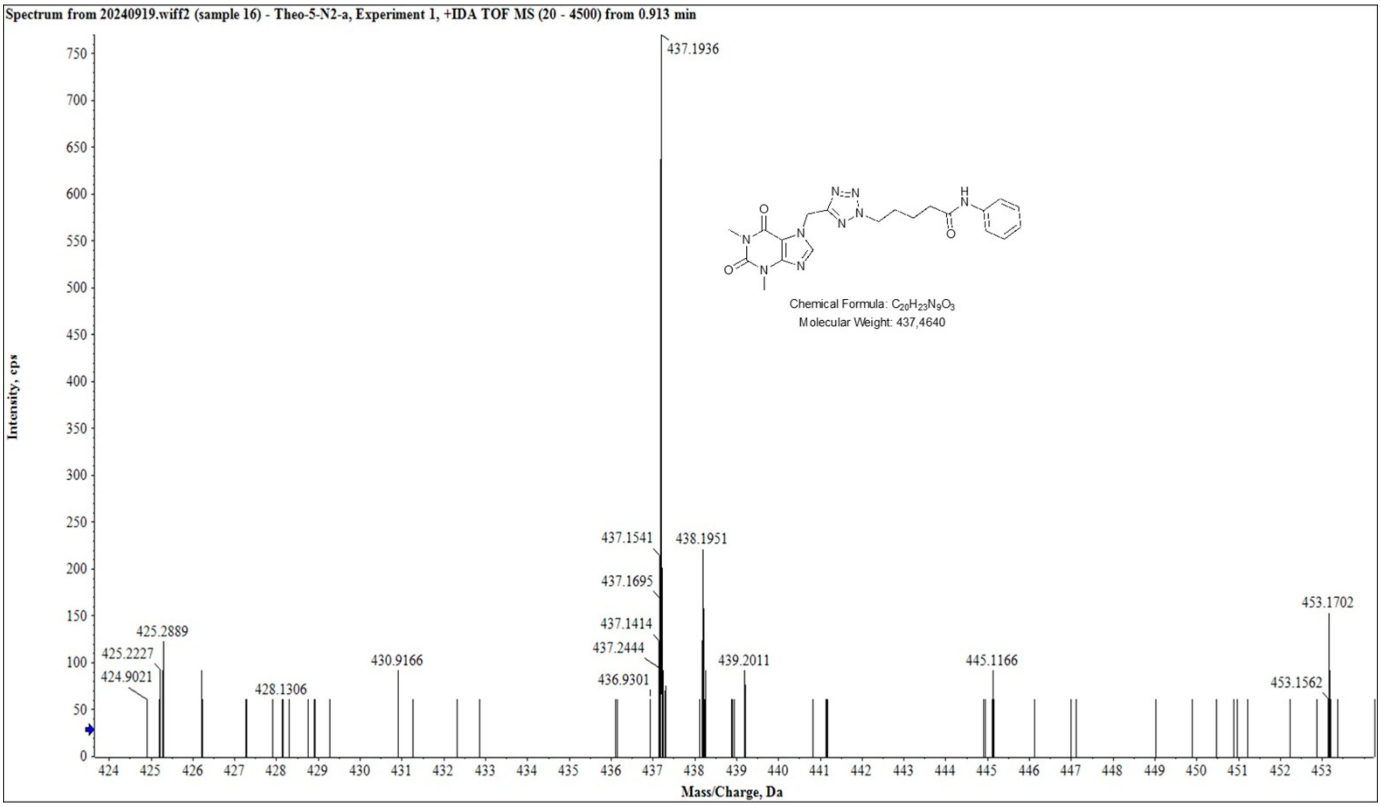

^{1}H-NMR of compound 12

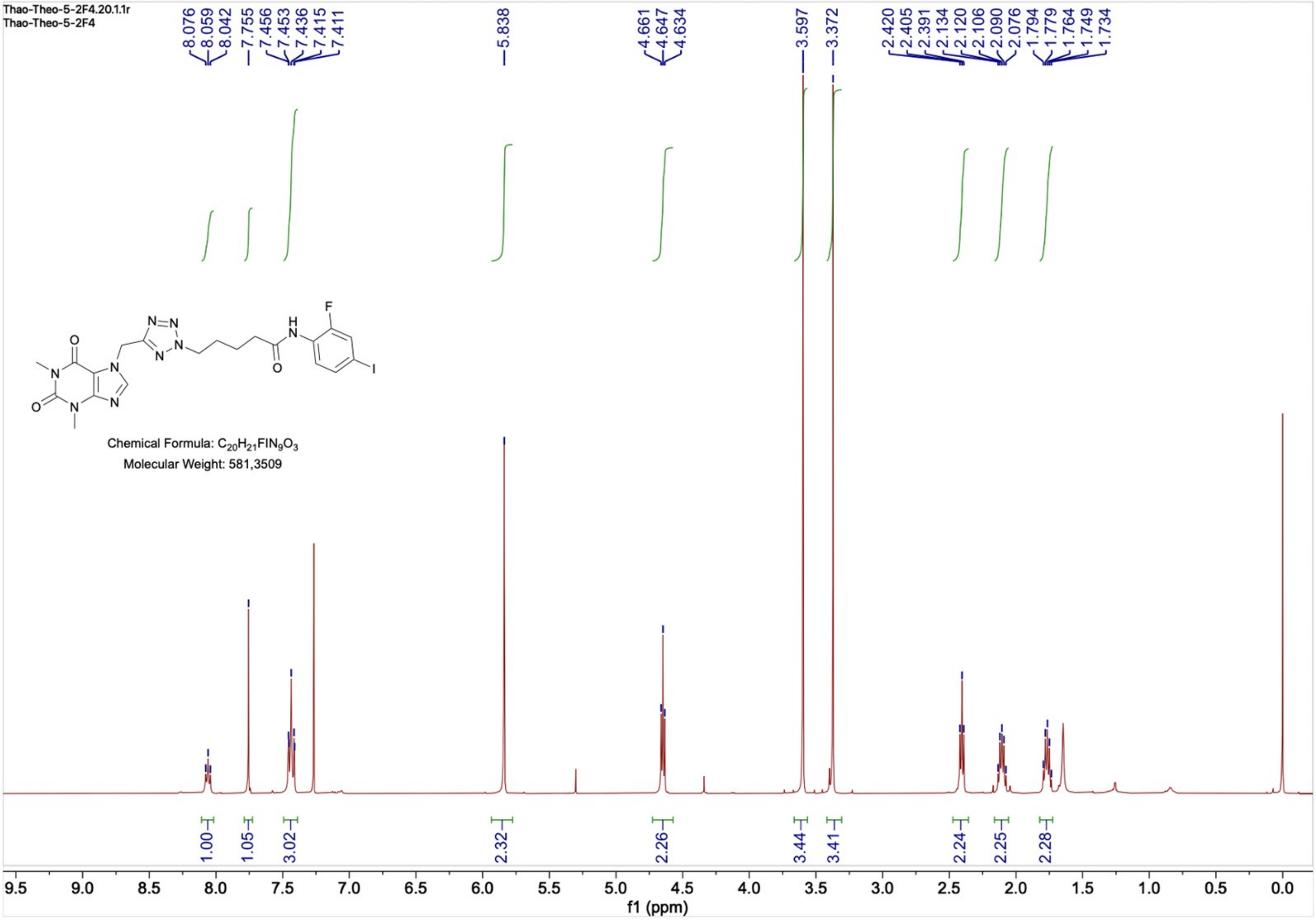

^{13}C-NMR of compound 12

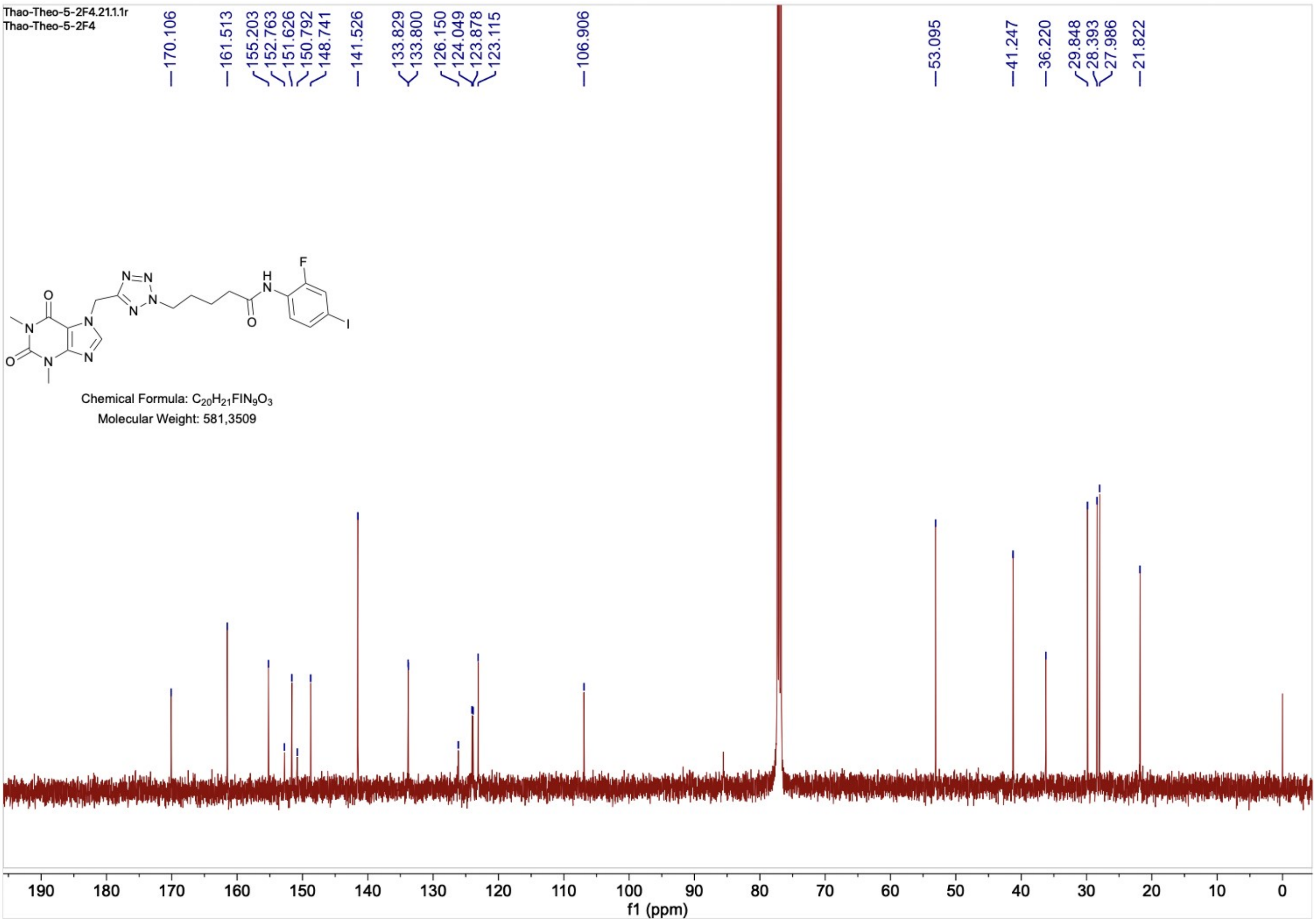

HR-MS of compound 12

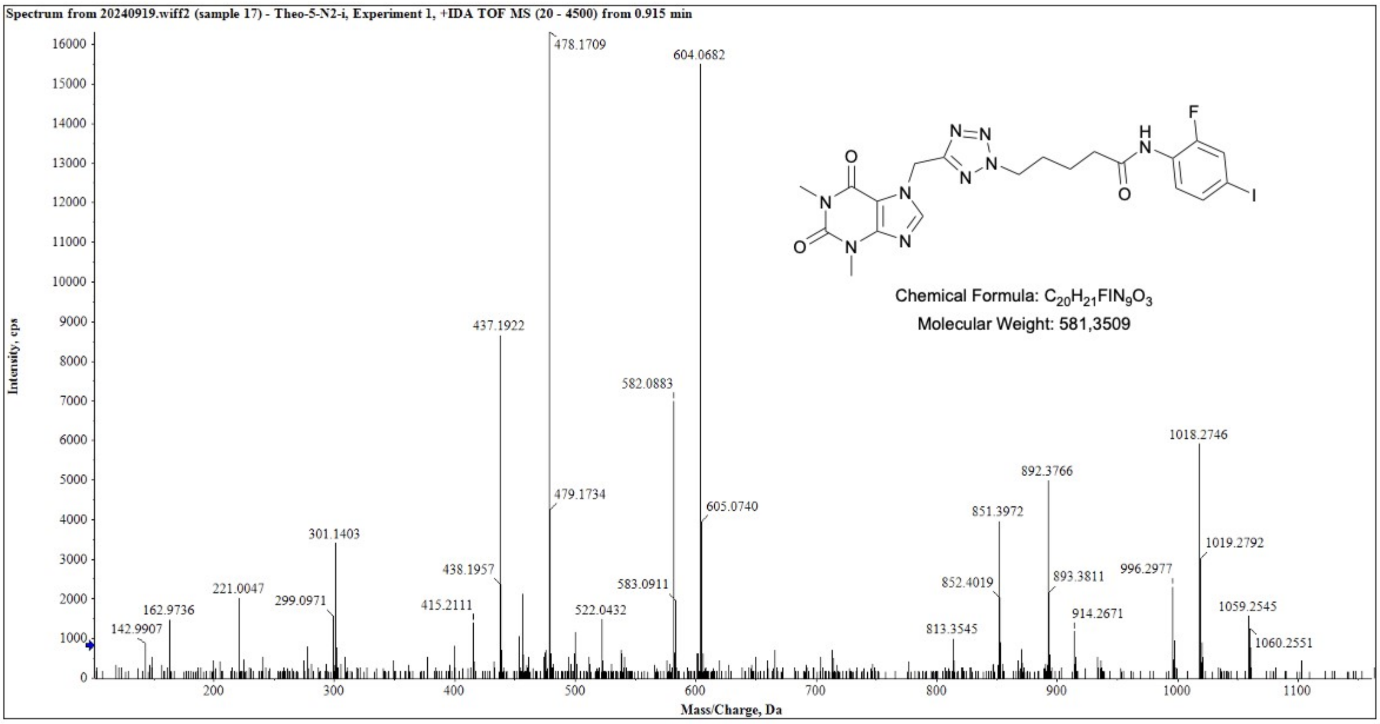

^{1}H-NMR of compound 13

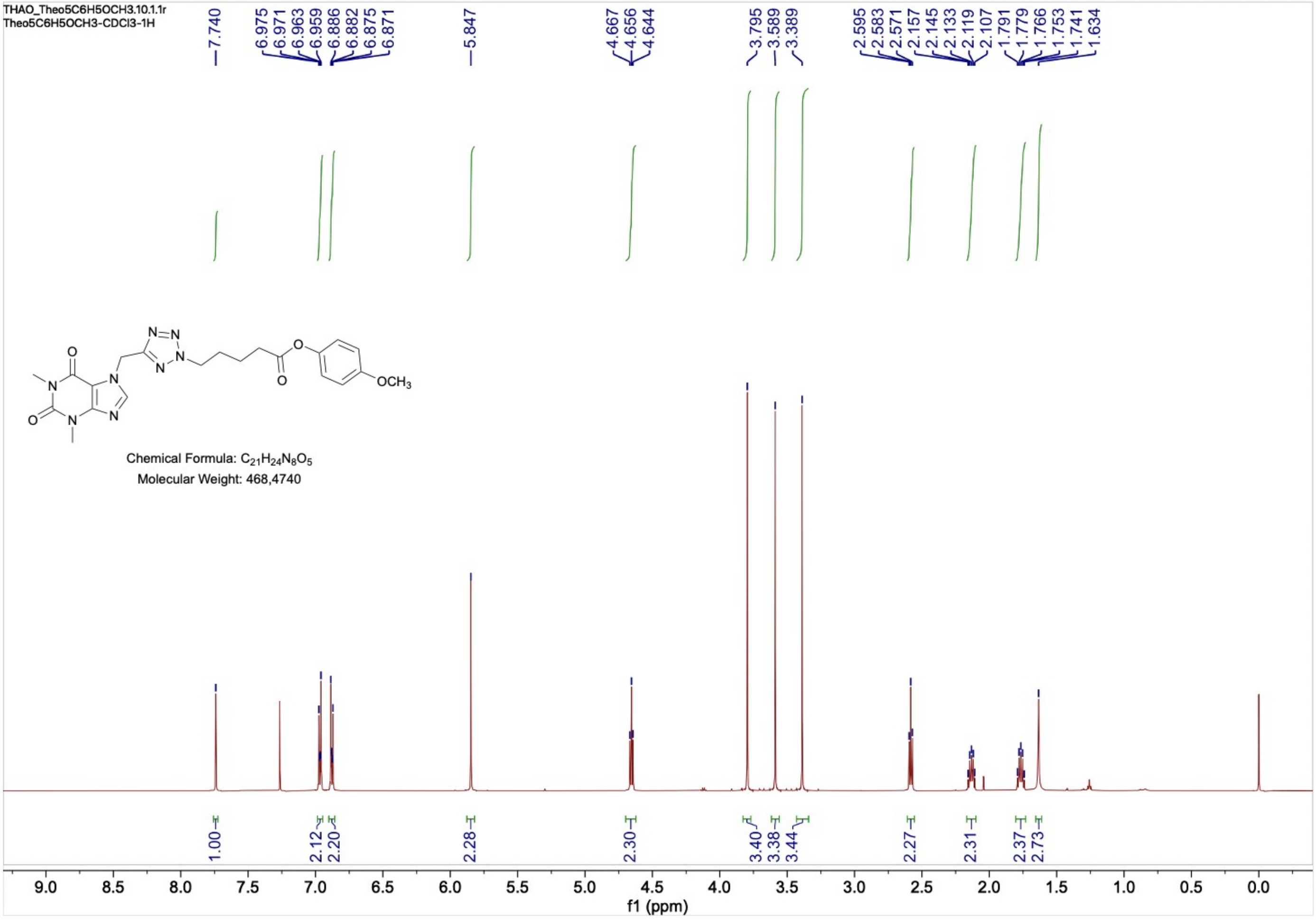

^{13}C-NMR of compound 13

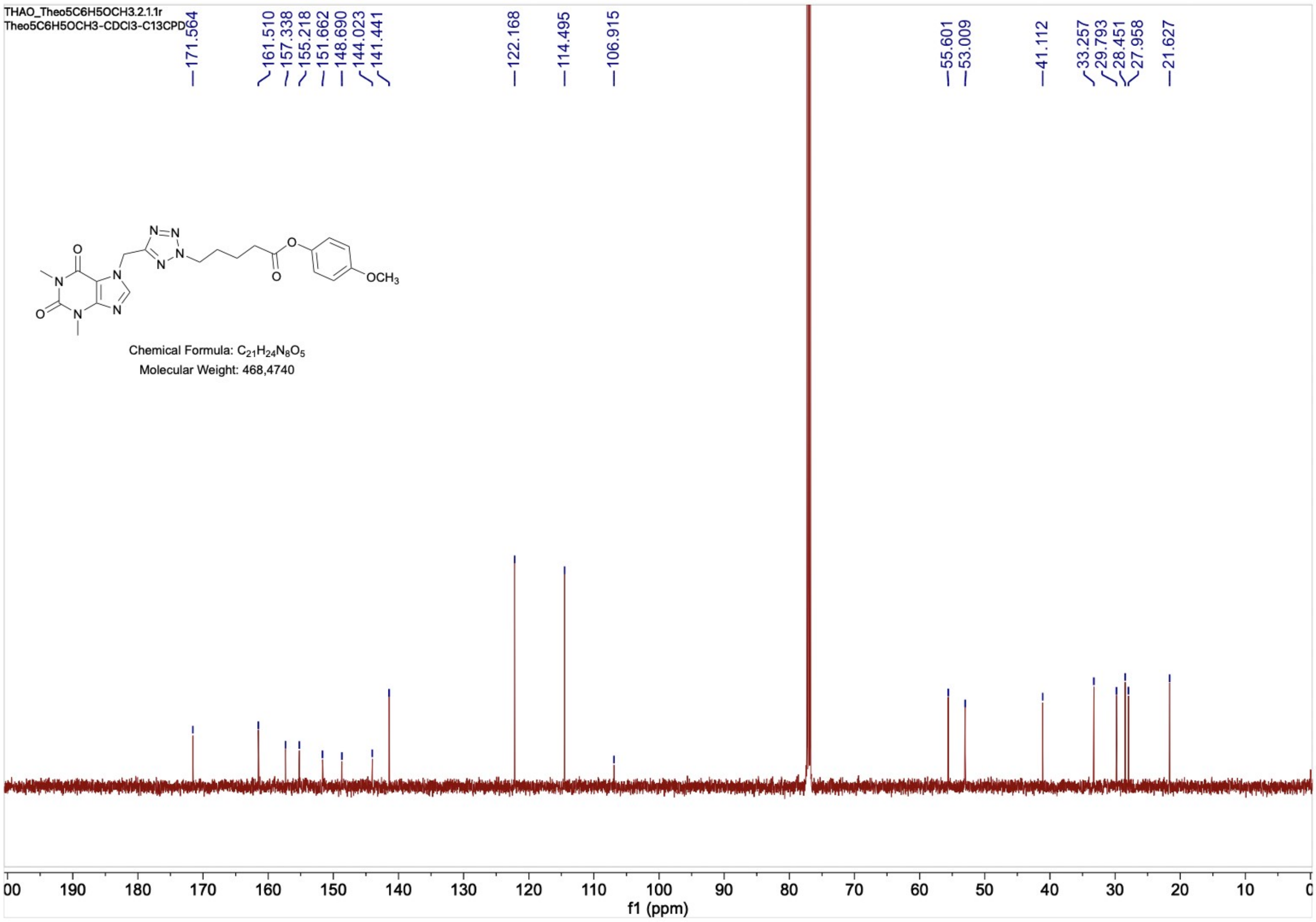

HR-MS of compound 13

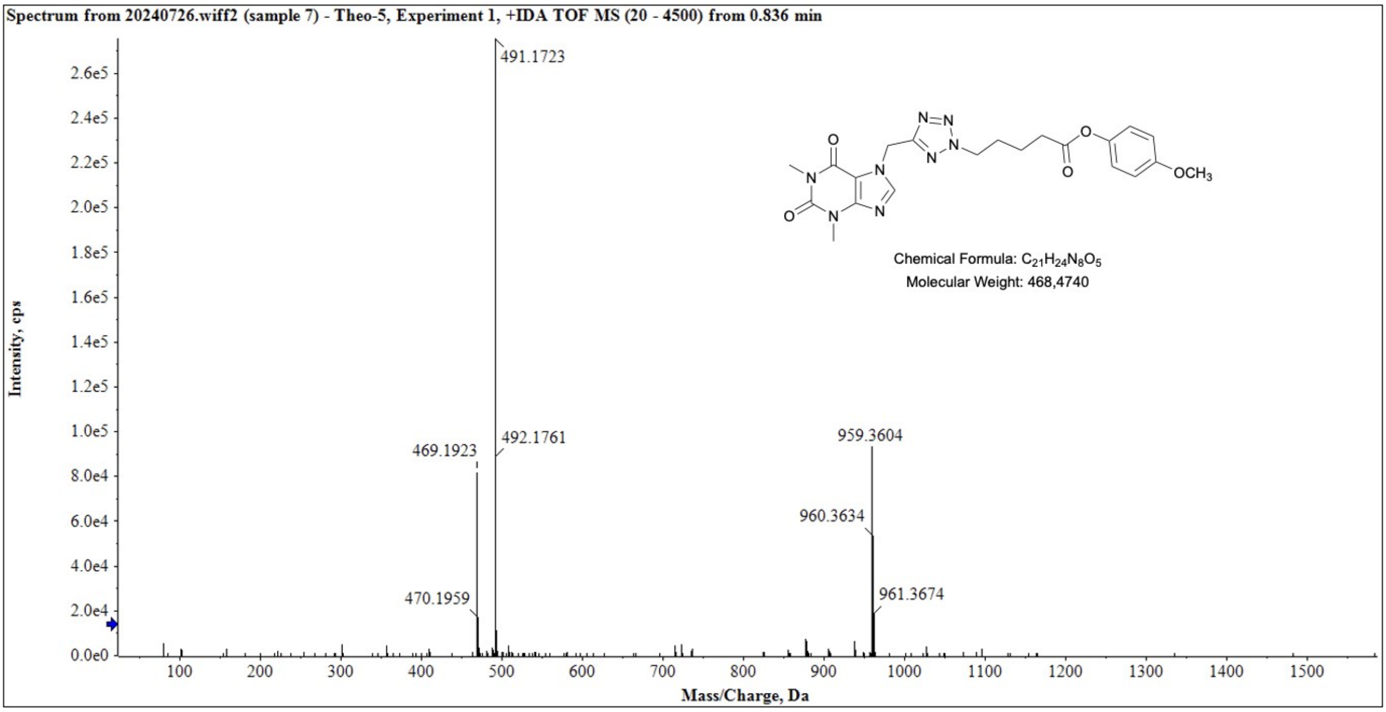

^{1}H-NMR of compound 14

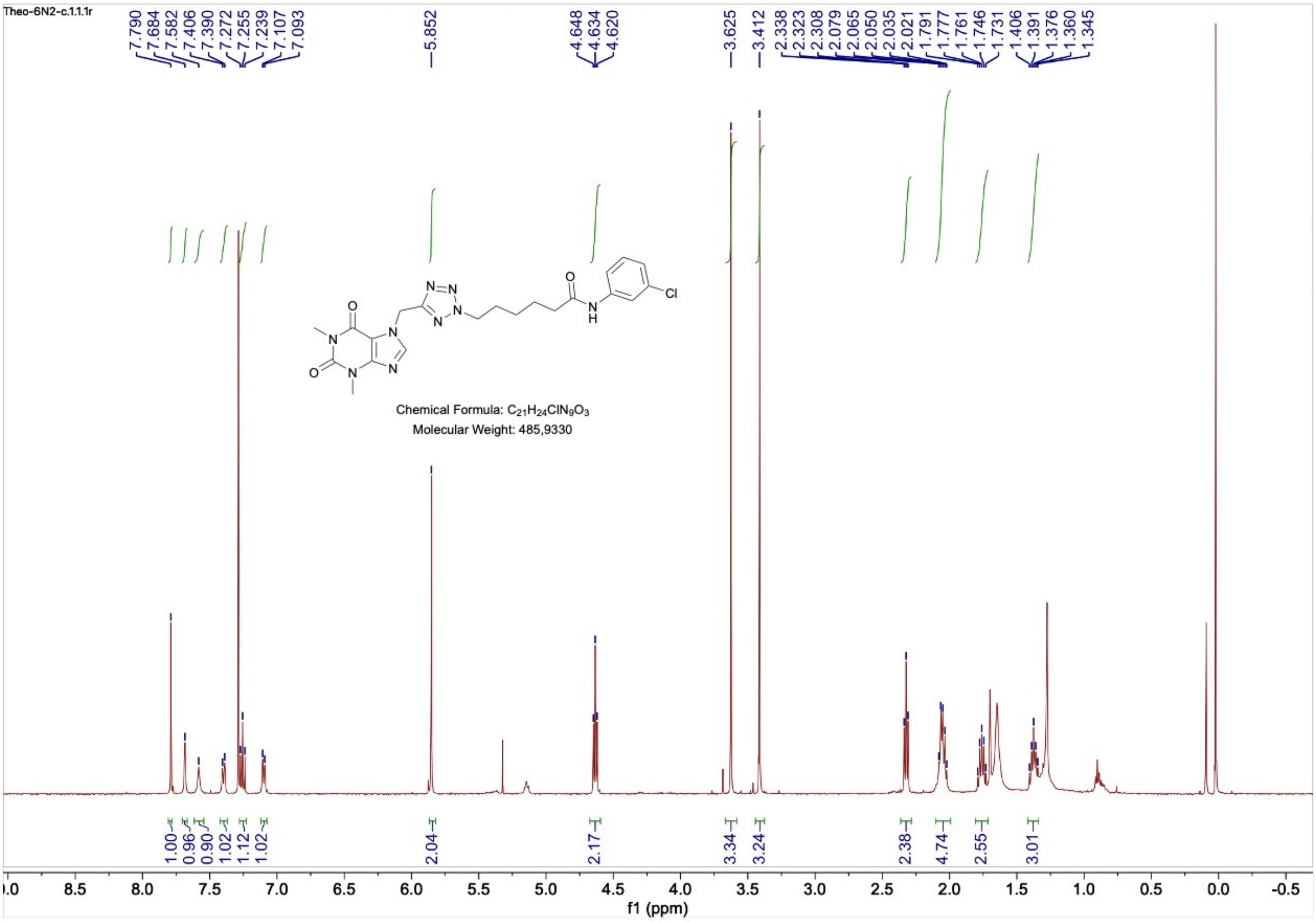

^{13}C-NMR of compound 14

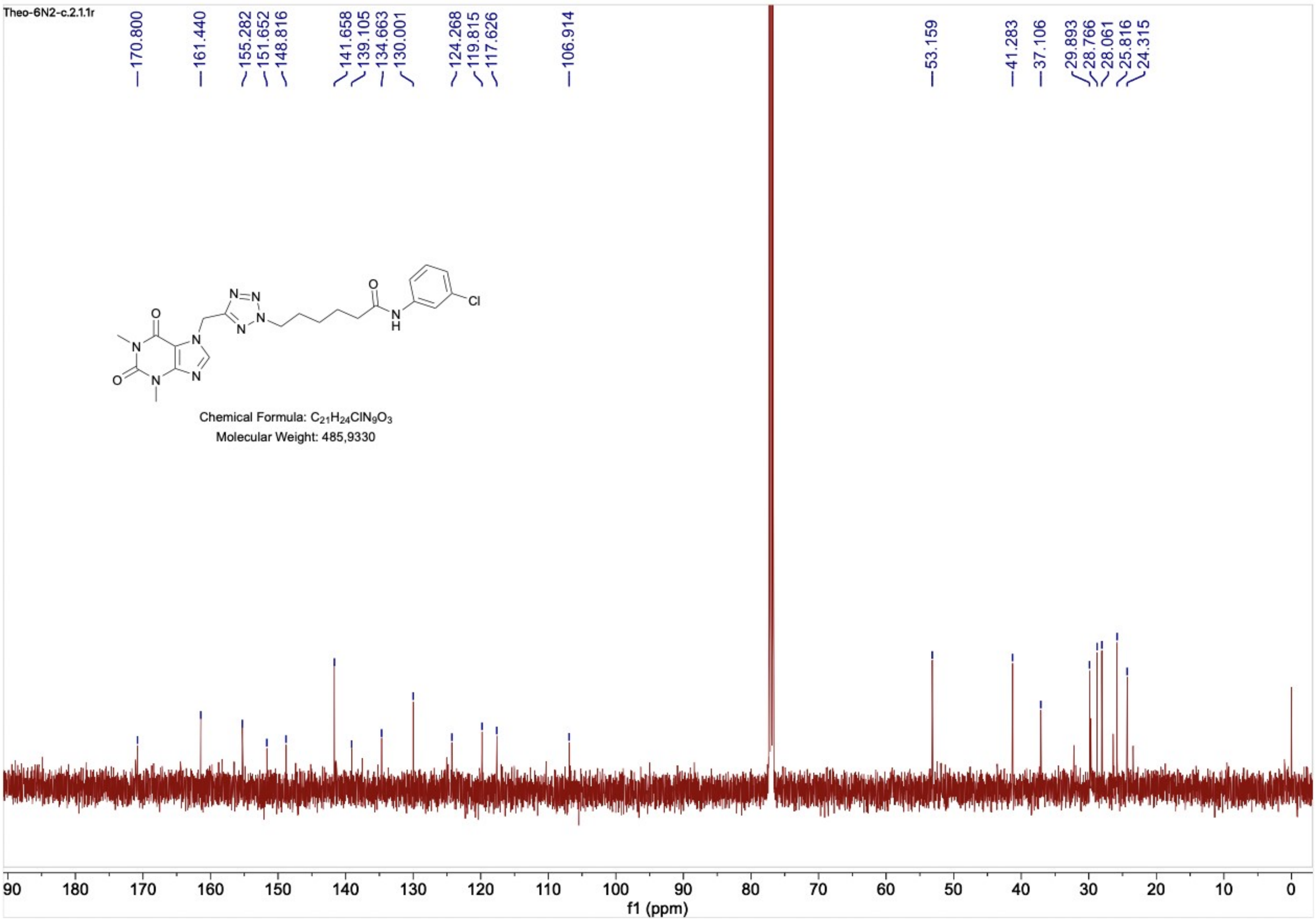

HR-MS of compound 14

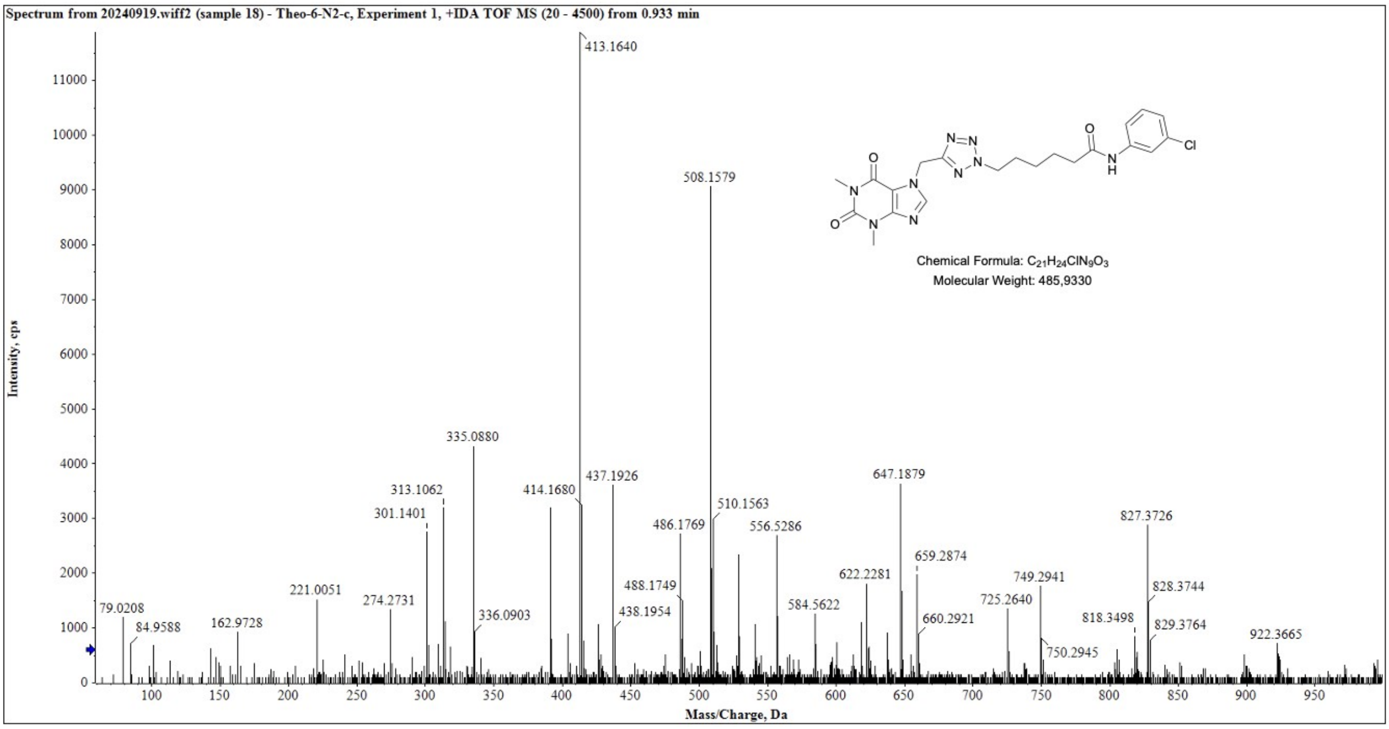

^{1}H-NMR of compound 15

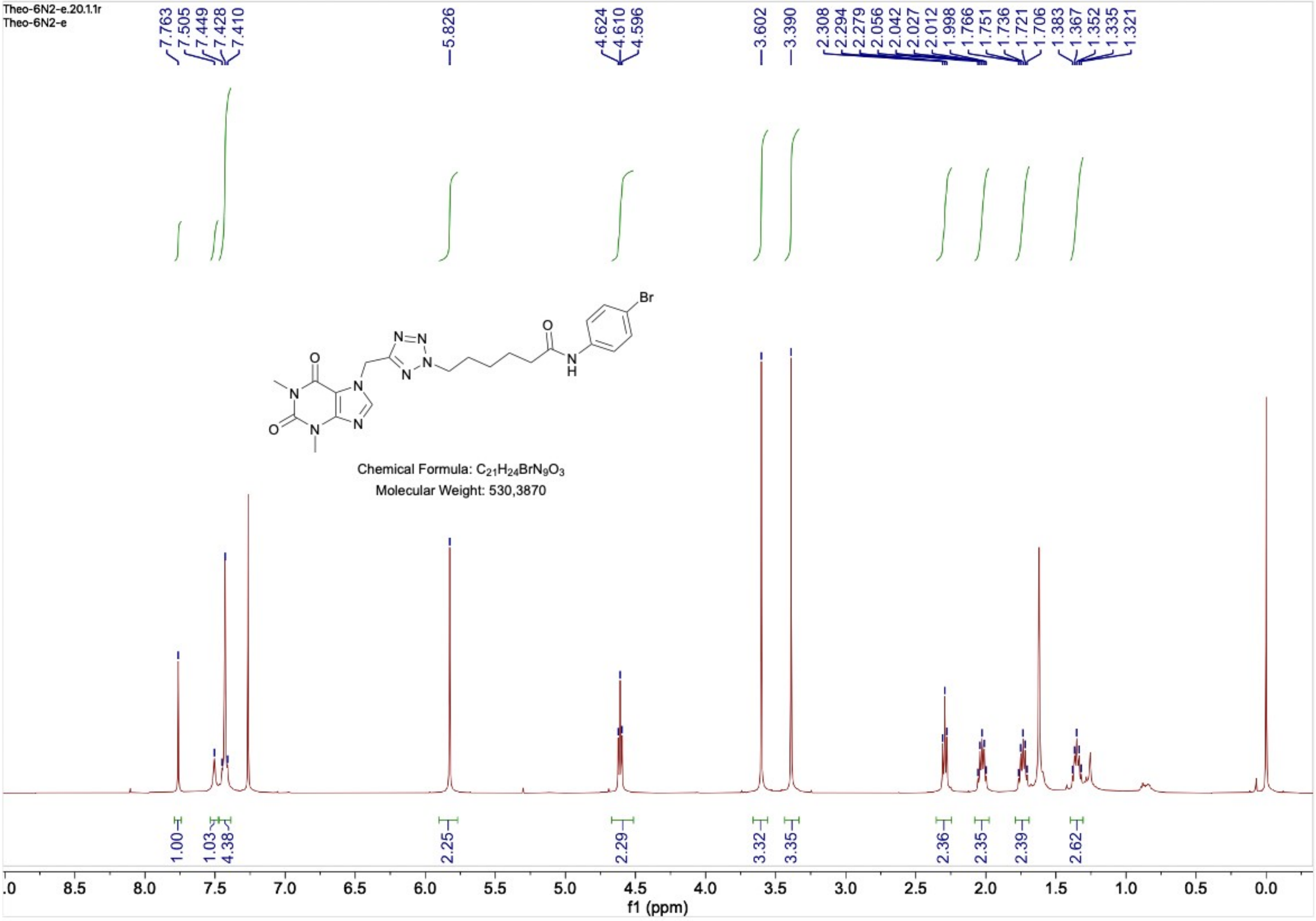

^{13}C-NMR of compound 15

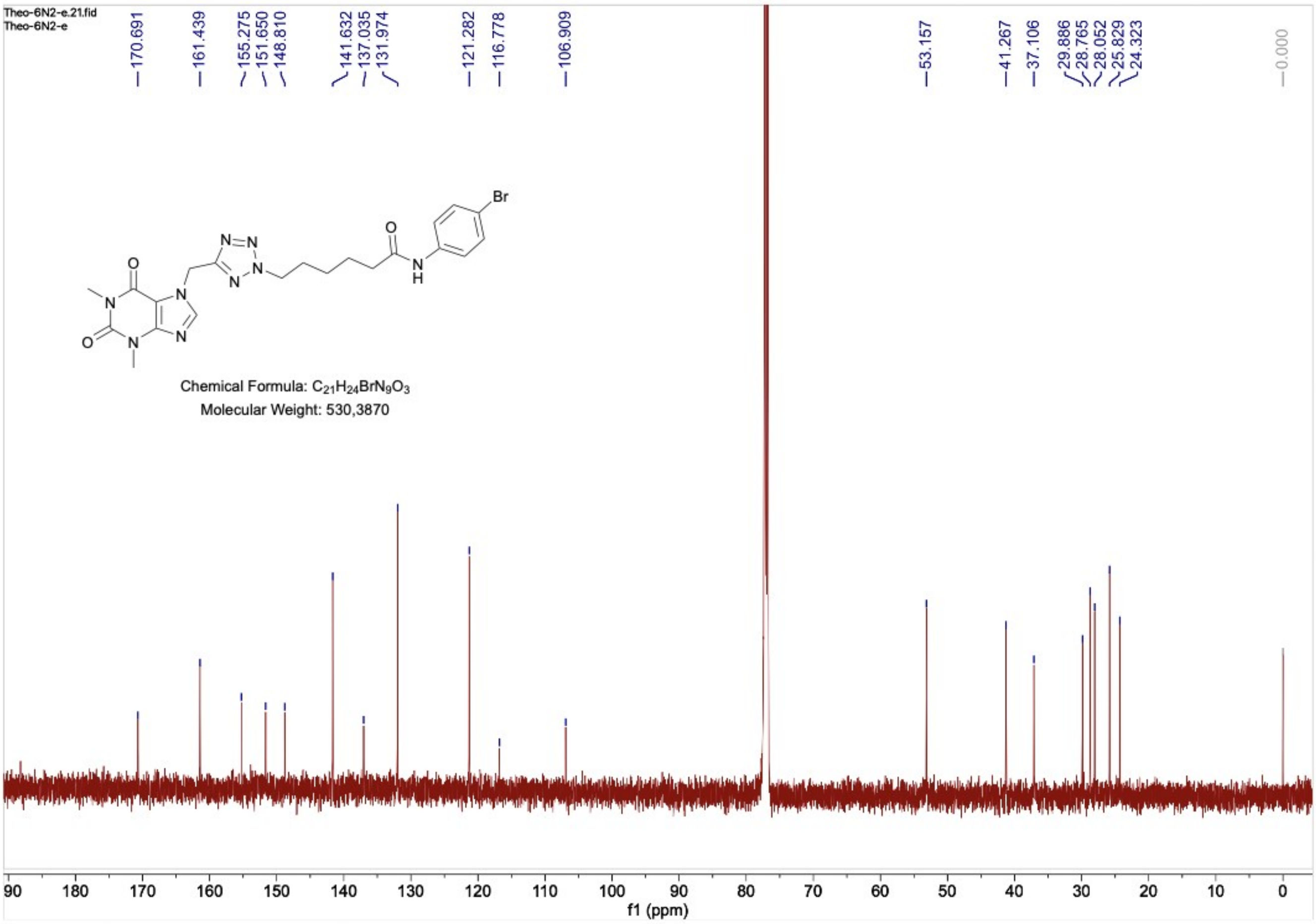

HR-MS of compound 15

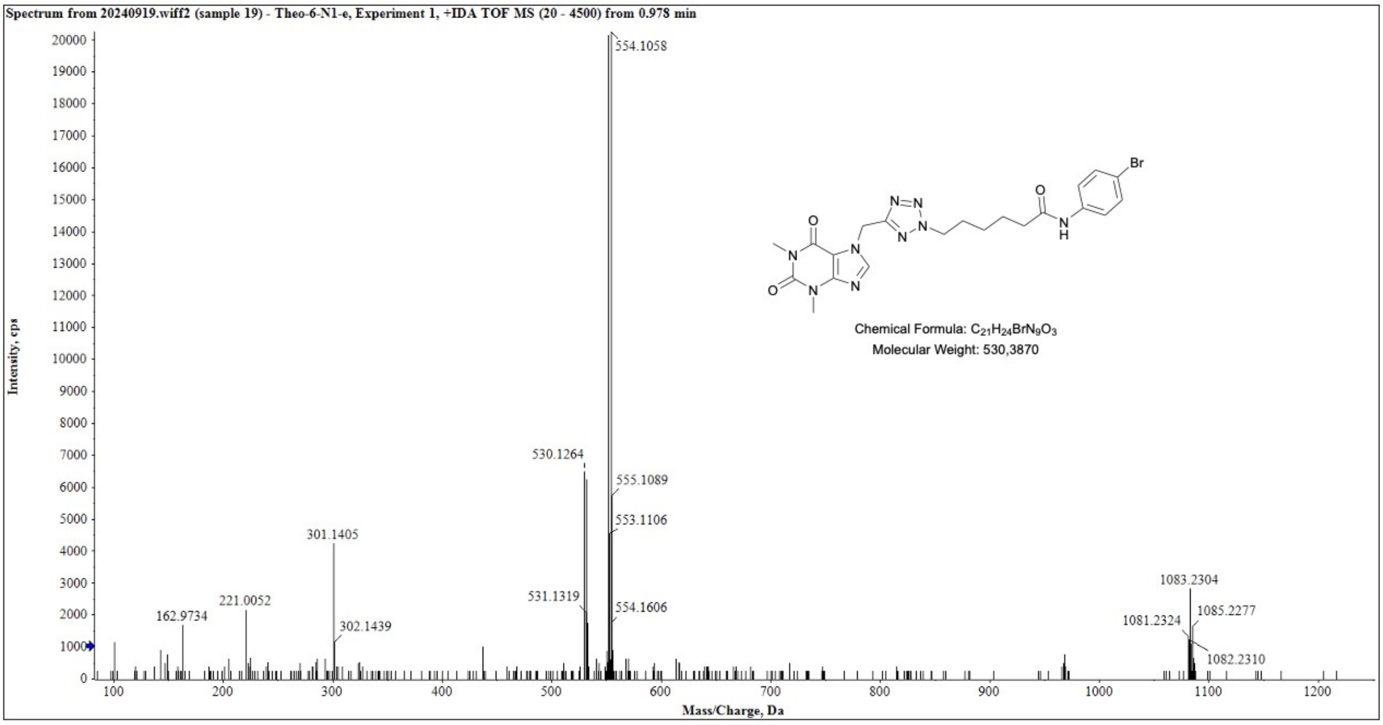

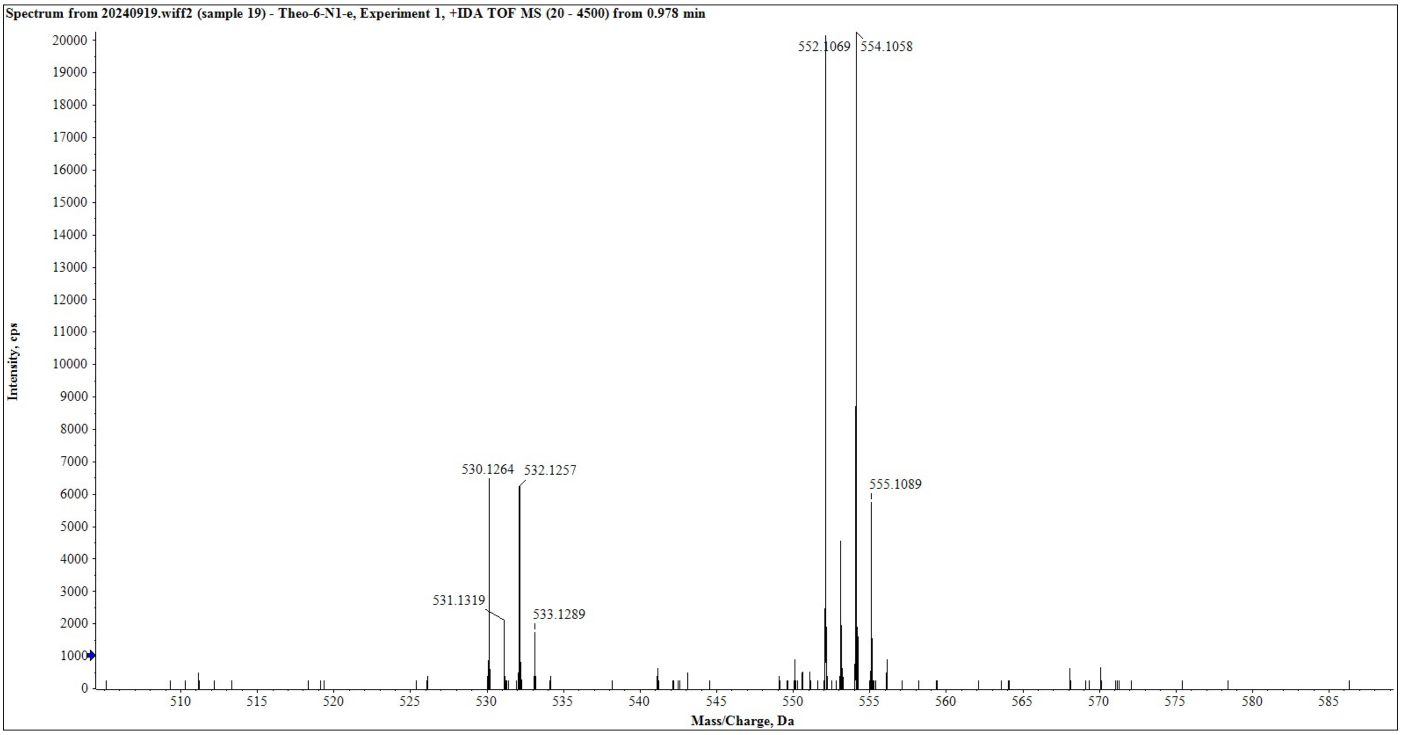

^{1}H-NMR of compound 16

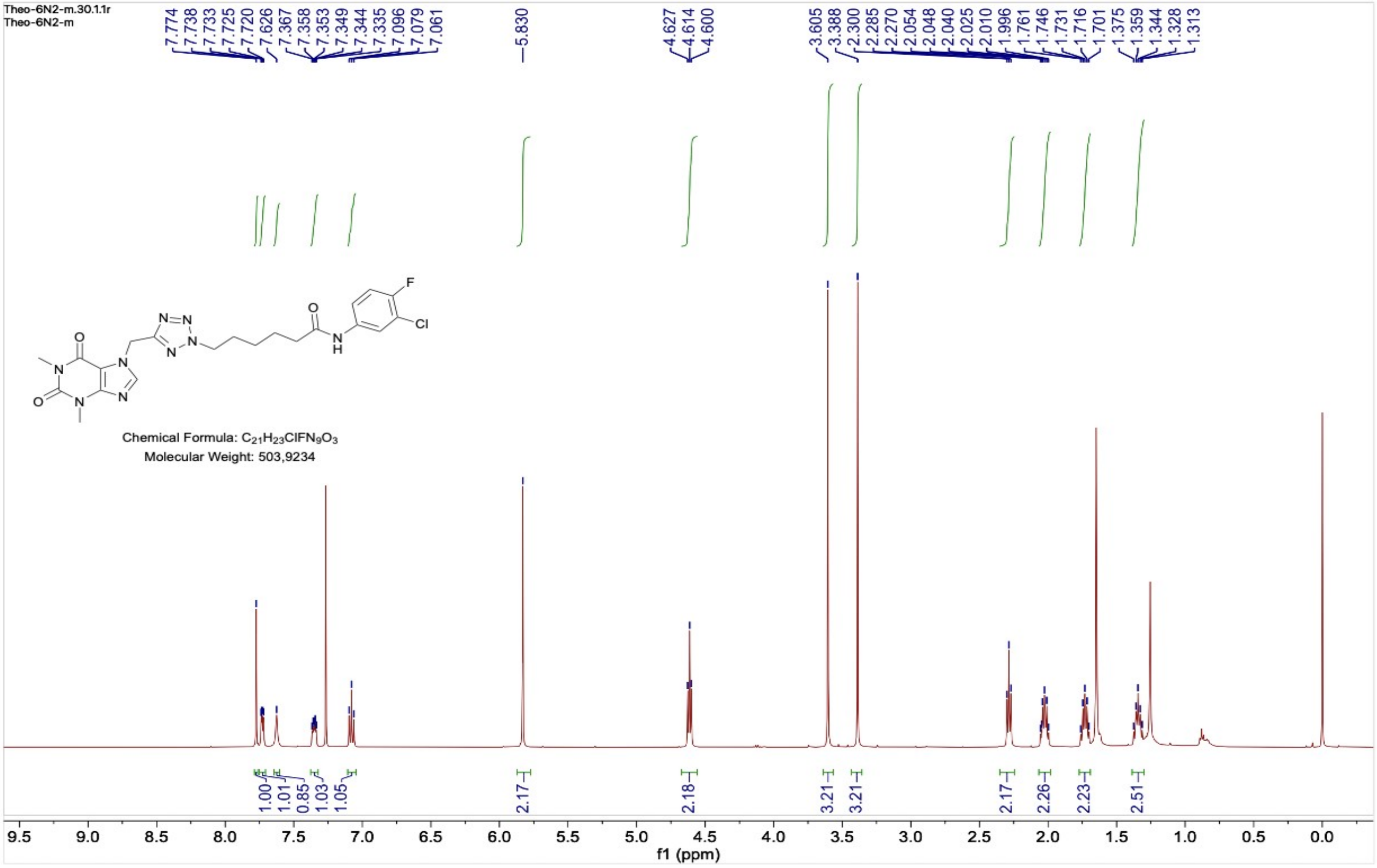

^{13}C-NMR of compound 16

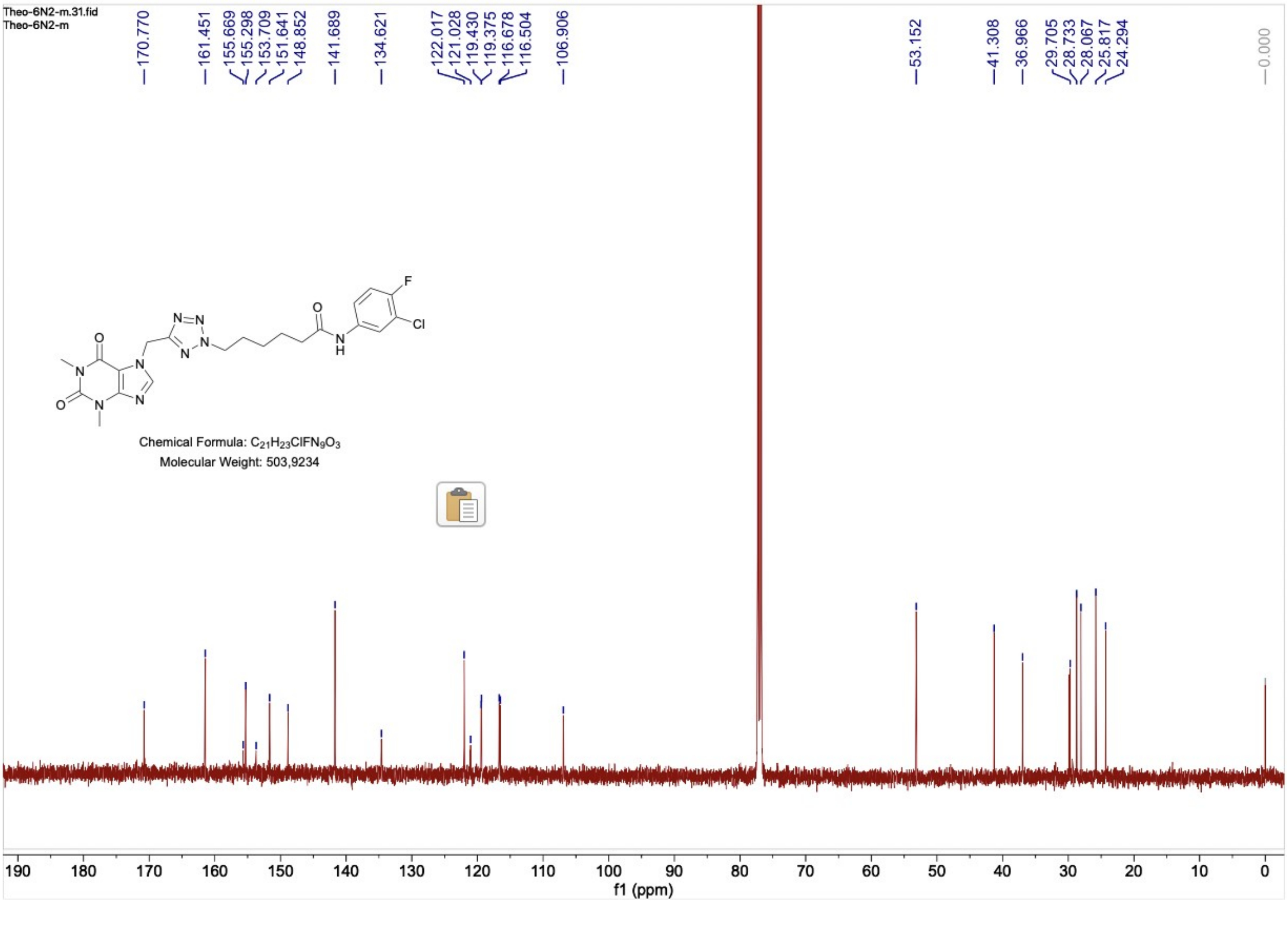

HR-MS of compound 16

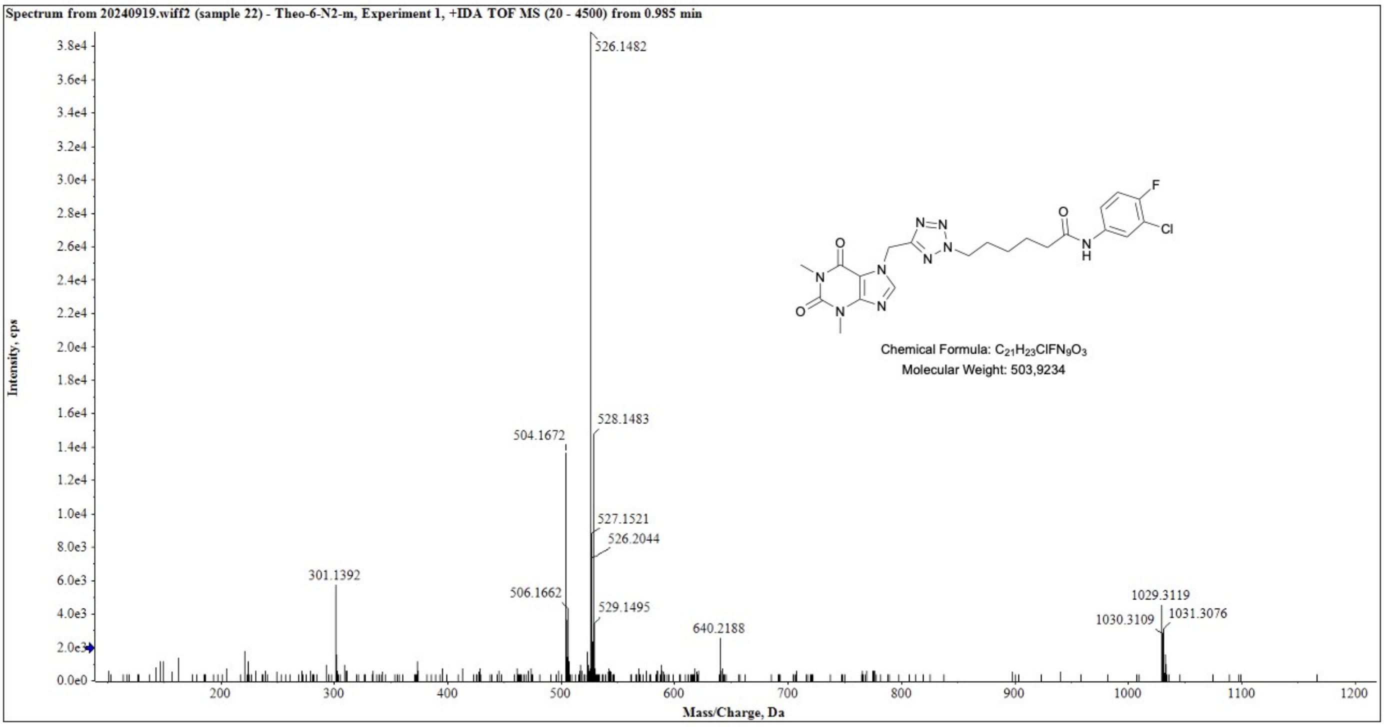

^{1}H-NMR of compound 17

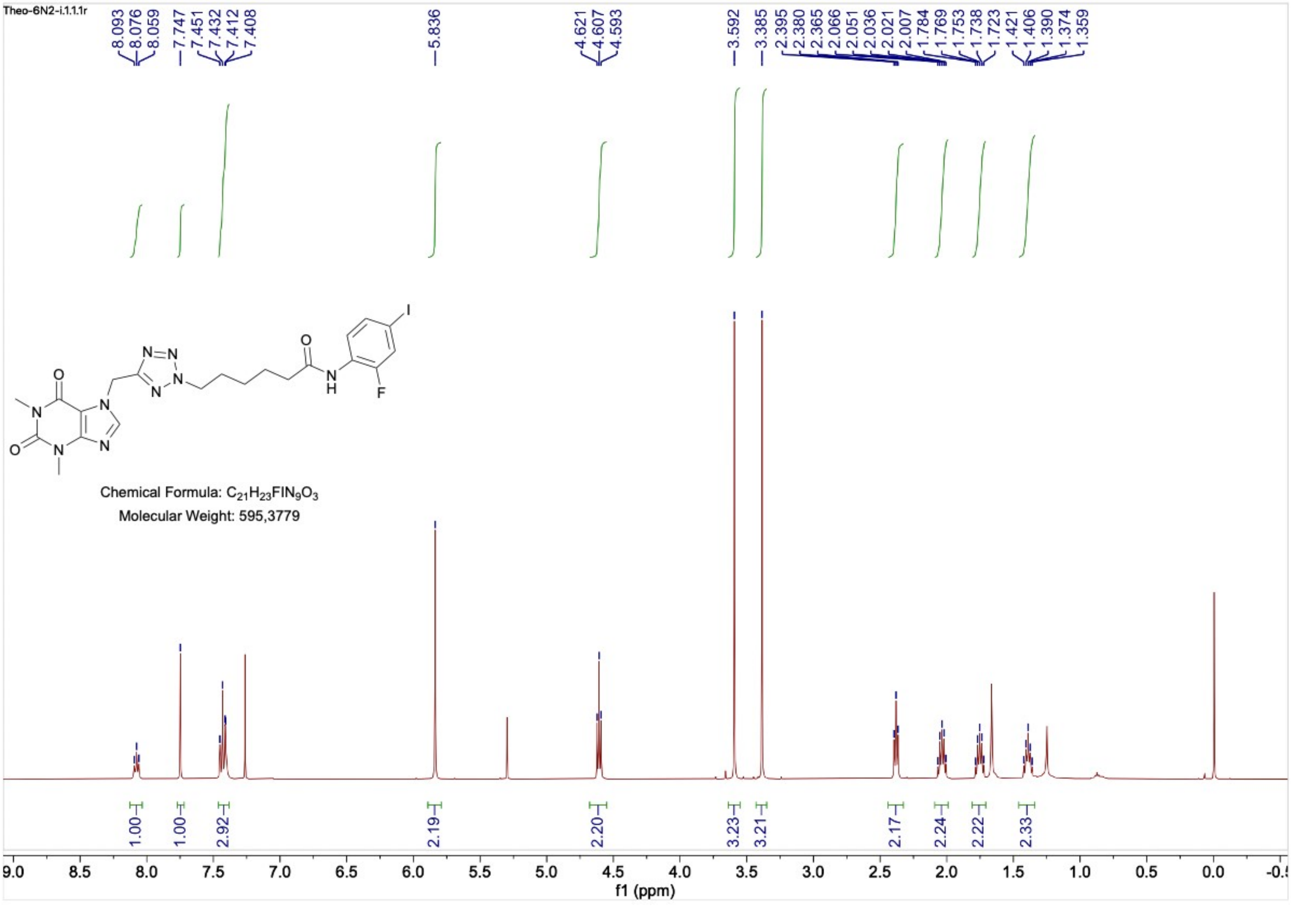

^{13}C-NMR of compound 17

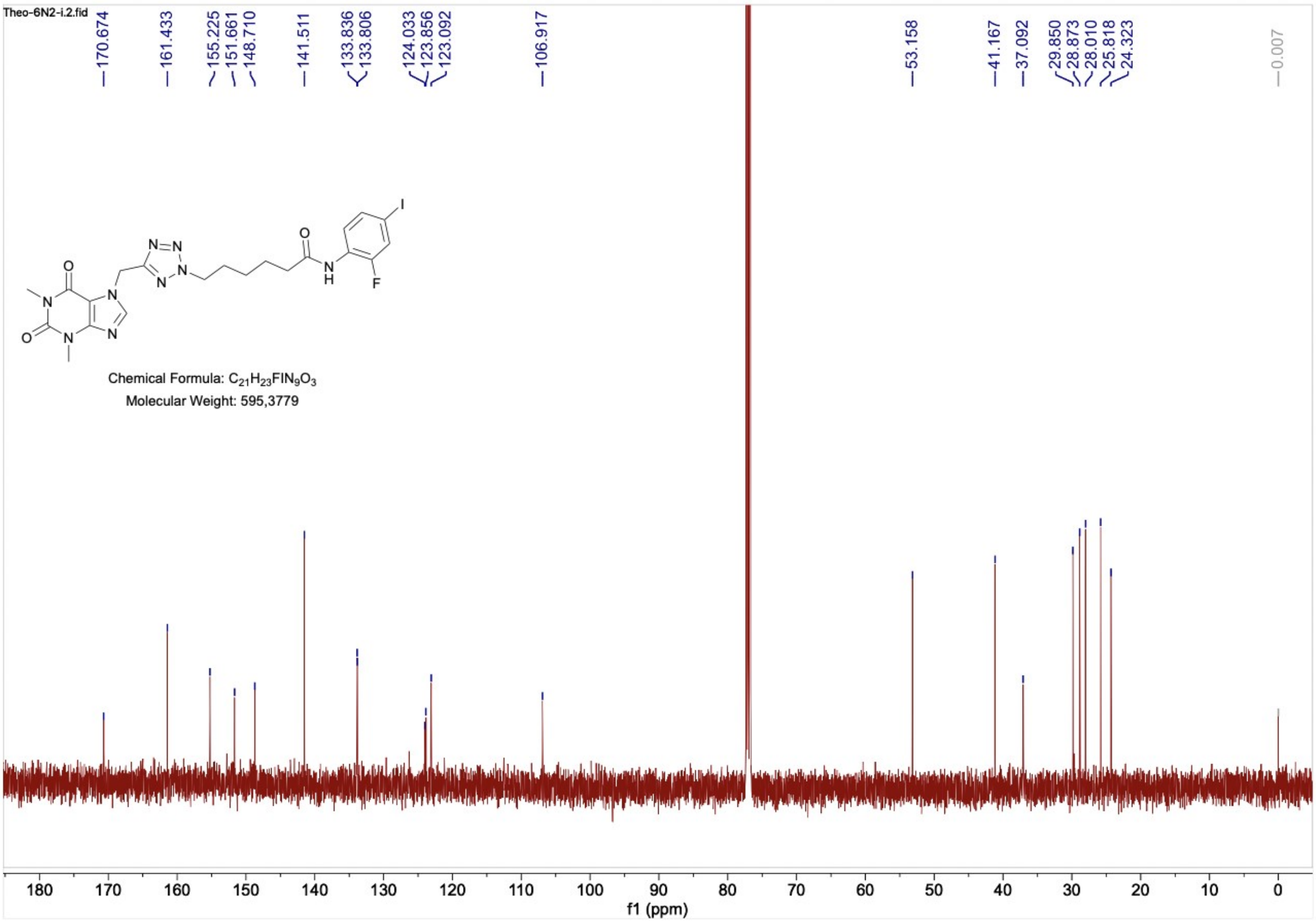

HR-MS of compound 17

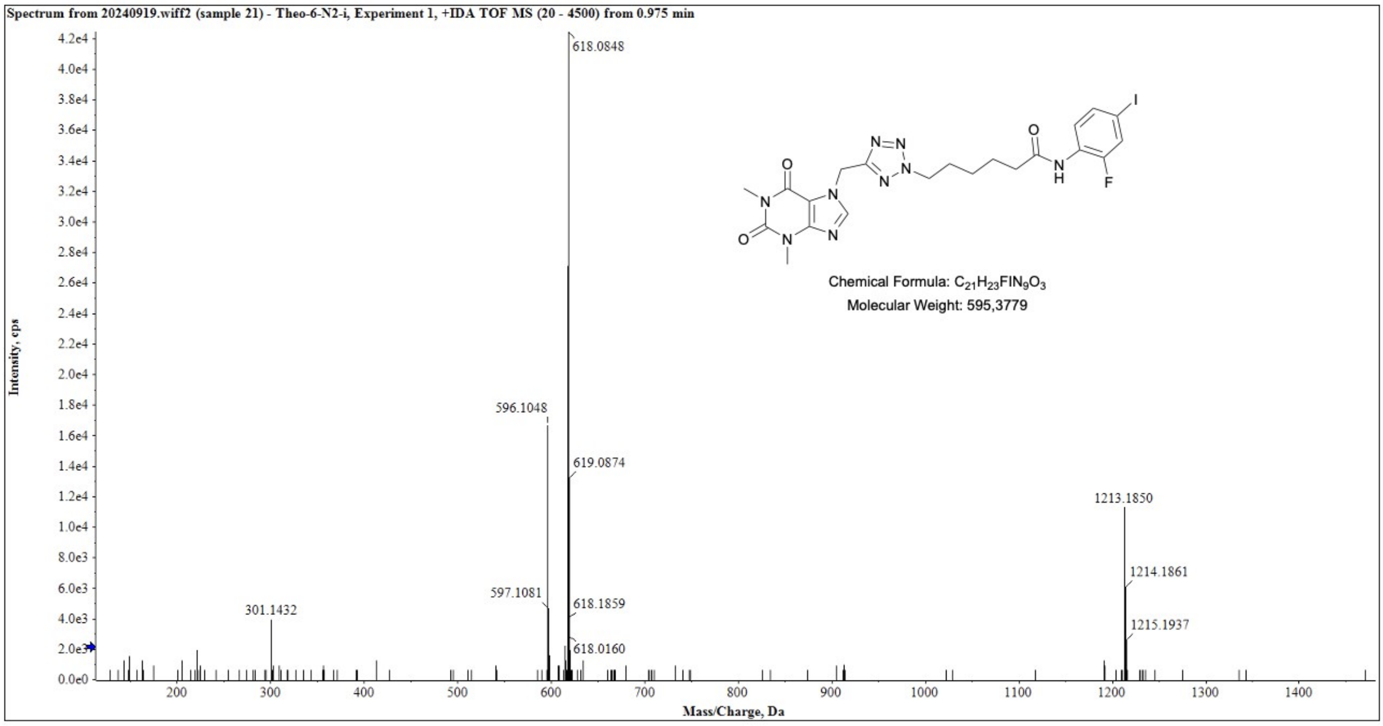

^{1}H-NMR of compound 18

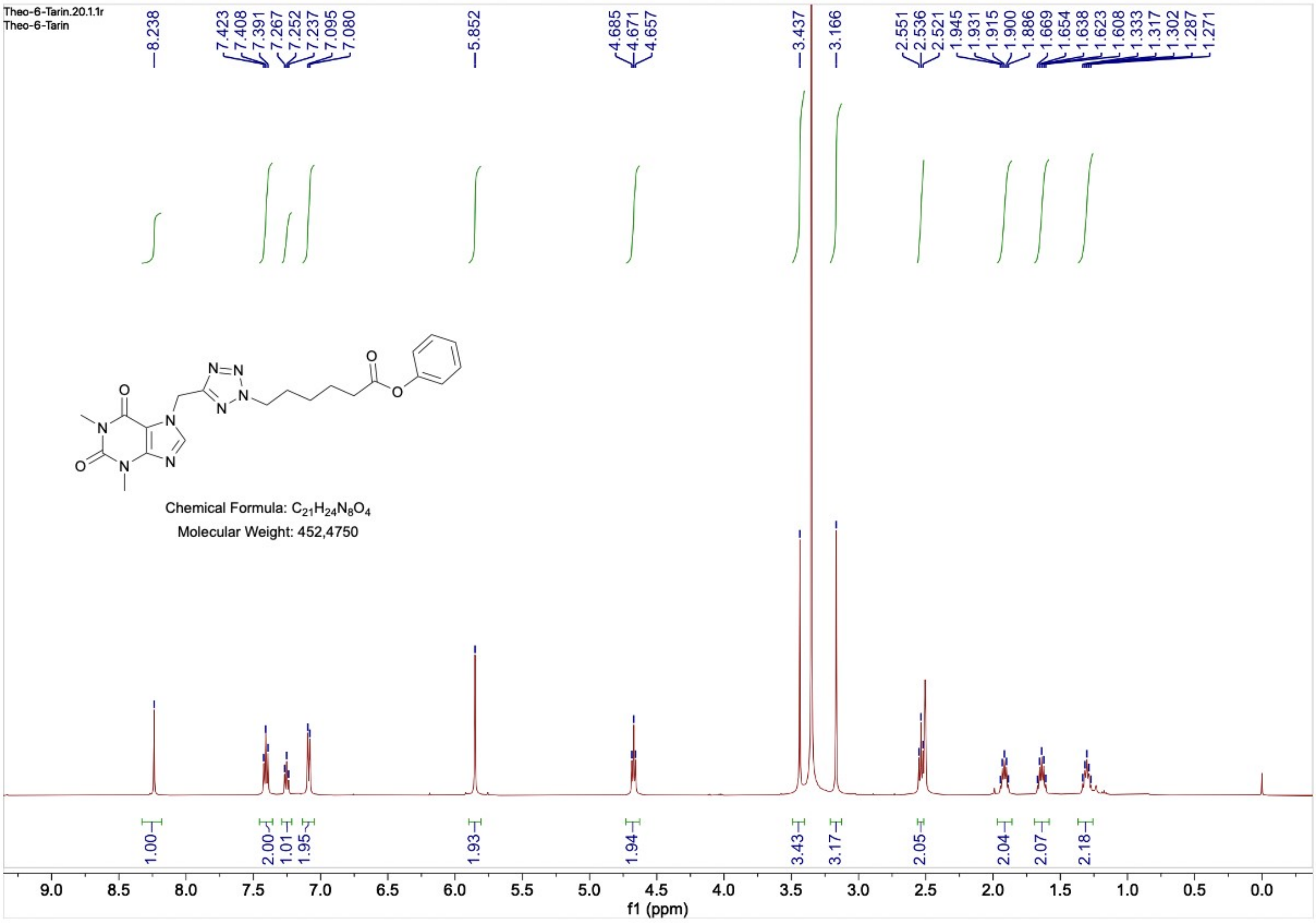

^{13}C-NMR of compound 18

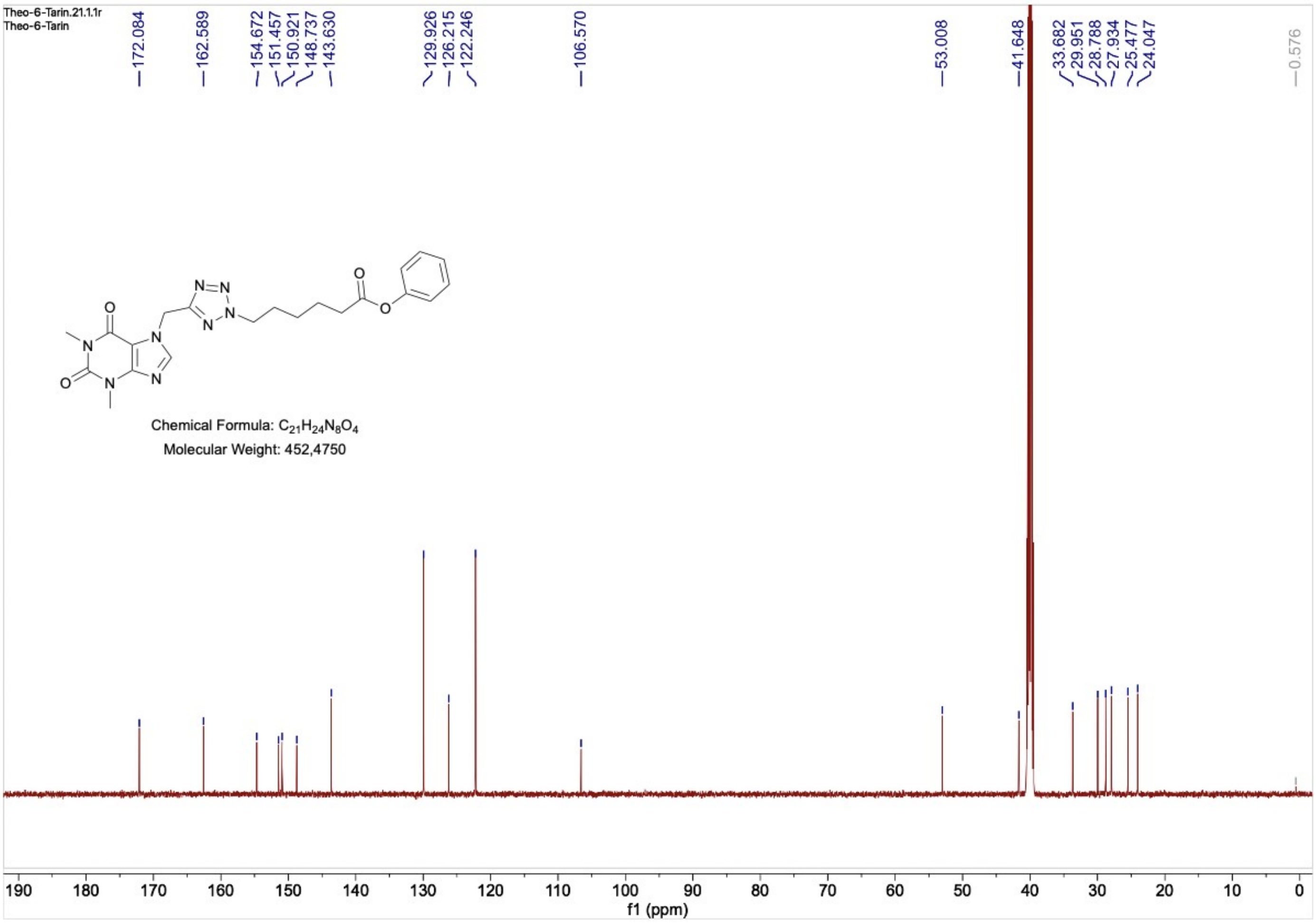

HR-MS of compound 18

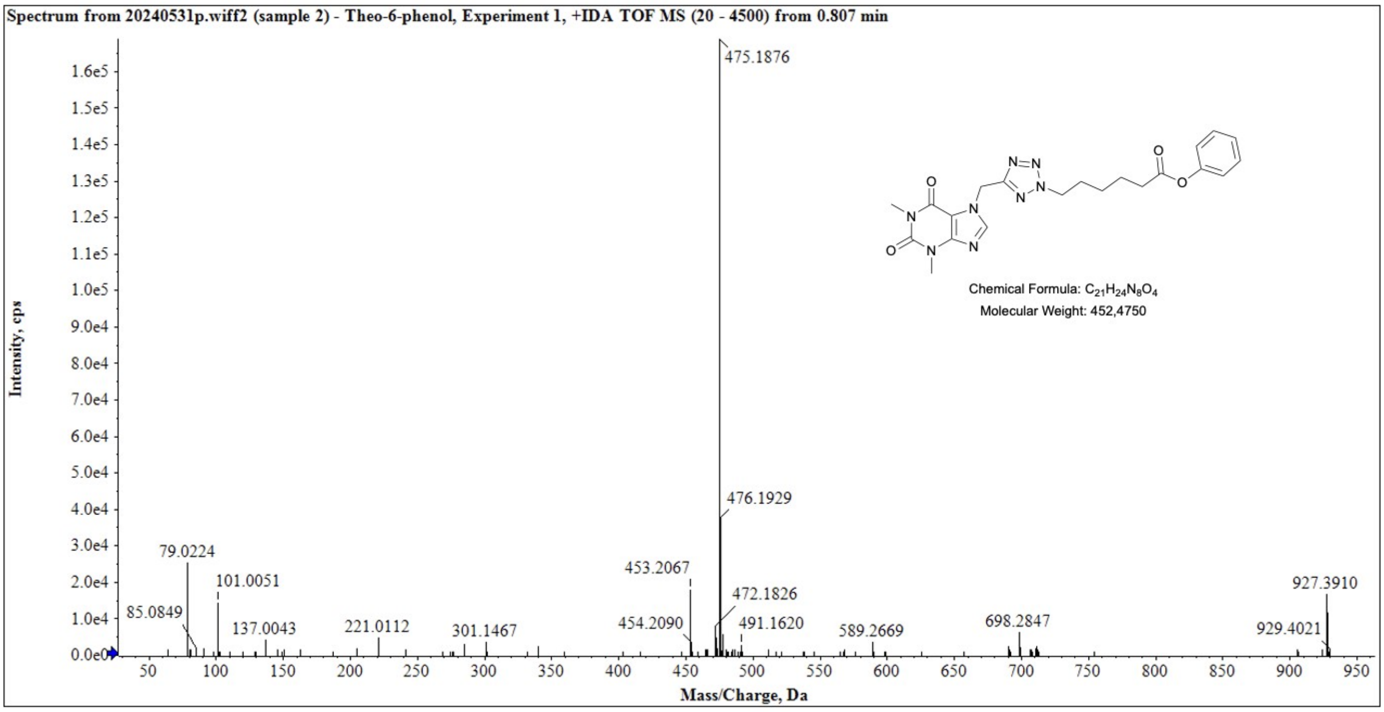

^{1}H-NMR of compound 19

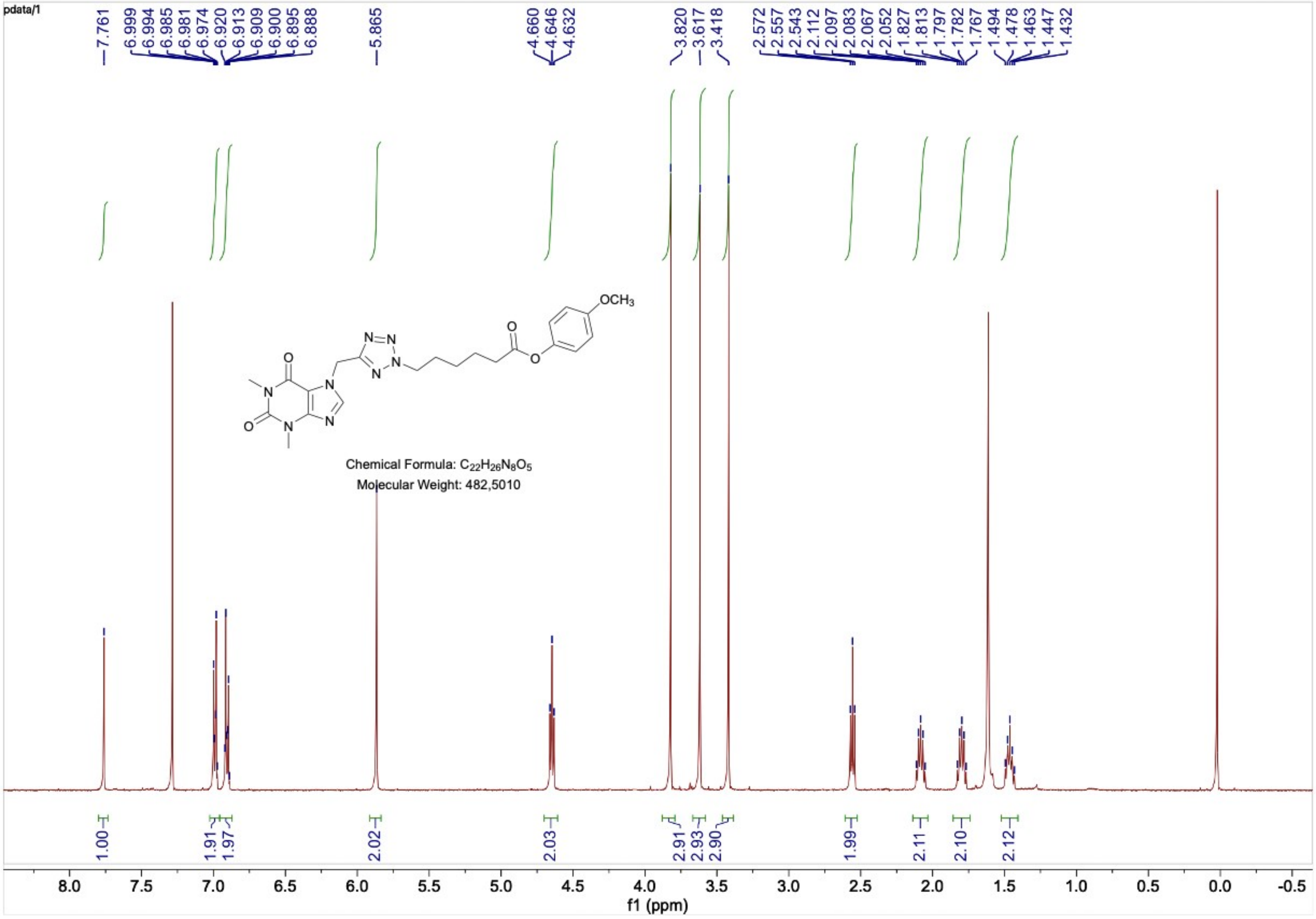

^{13}C-NMR of compound 19

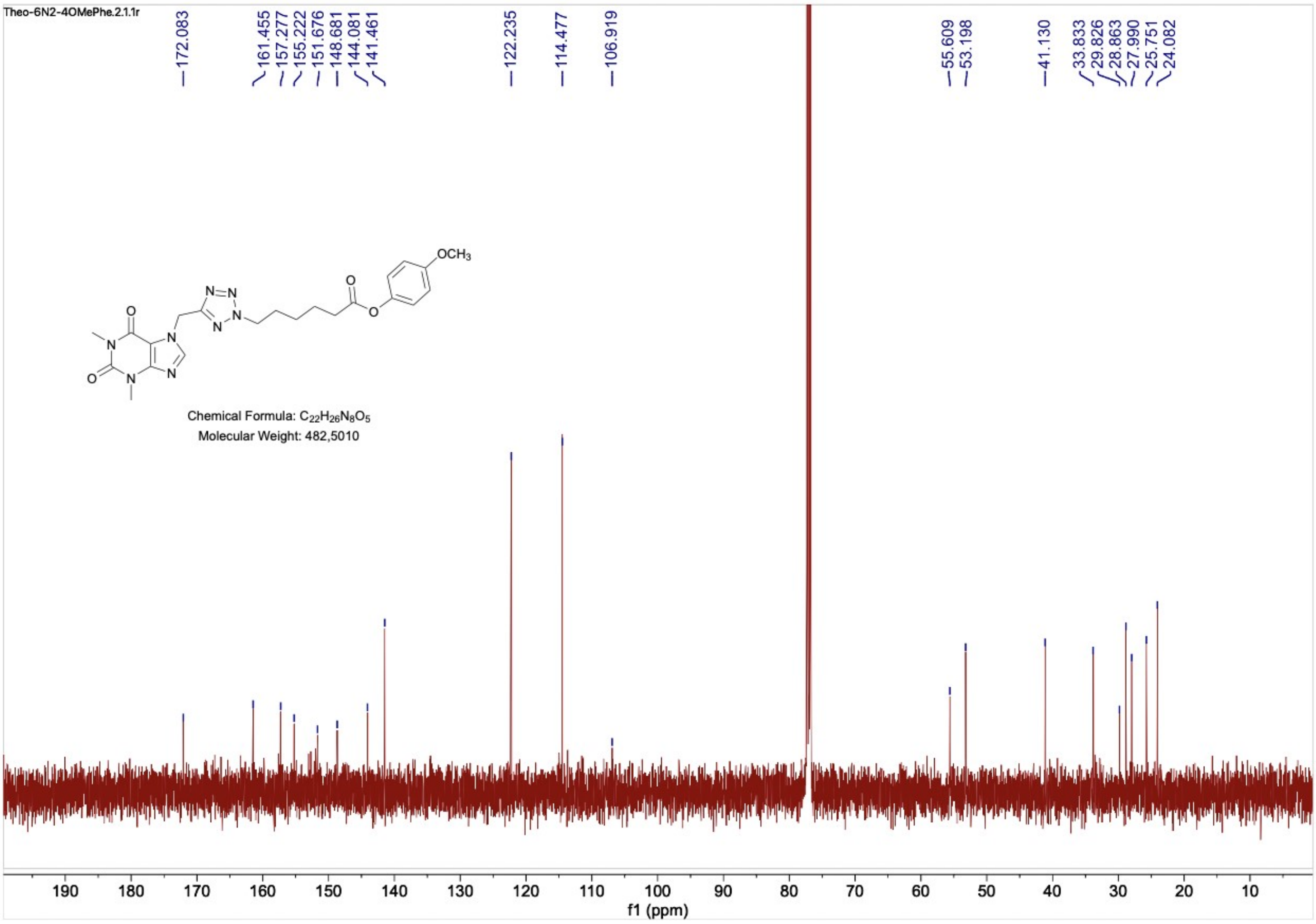

HR-MS of compound 19

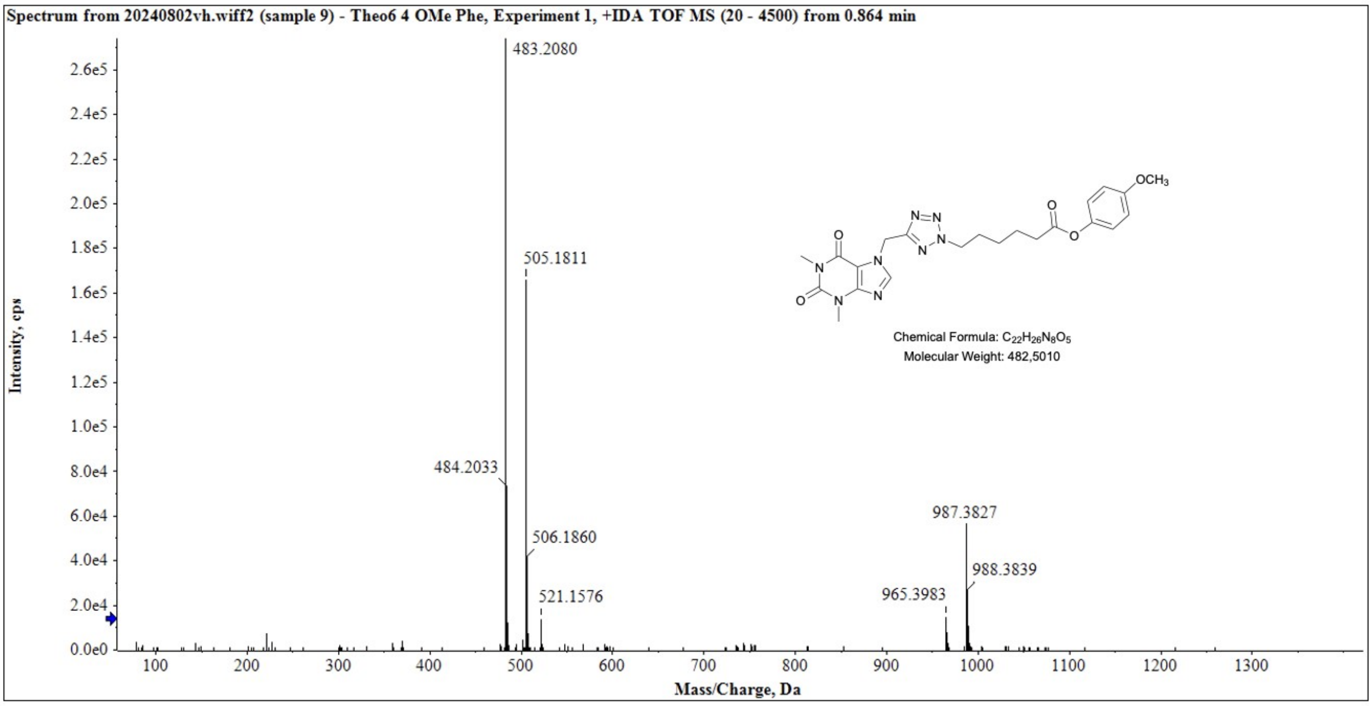

^{1}H-NMR of compound 20

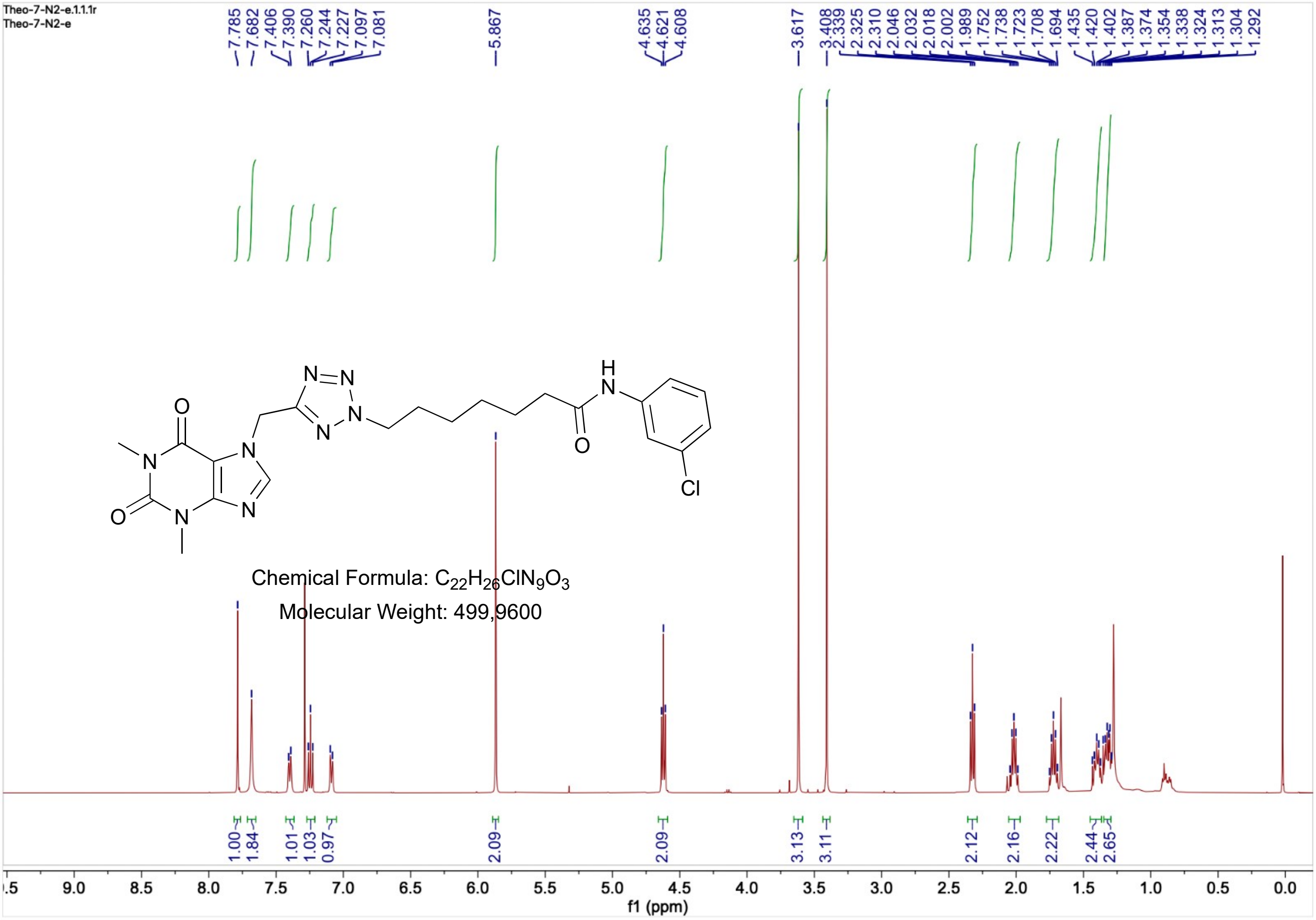

^{13}C-NMR of compound 20

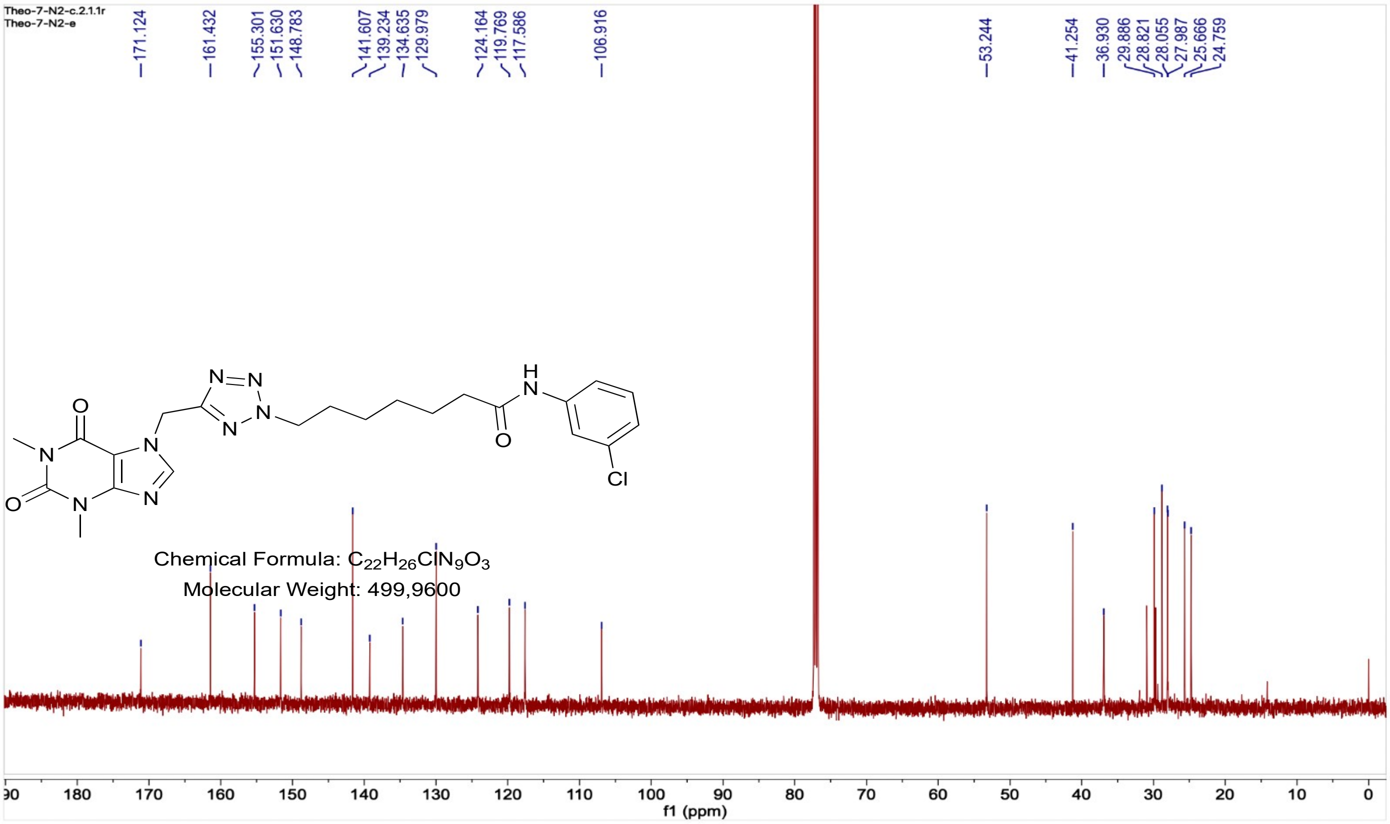

HR-MS of compound 20

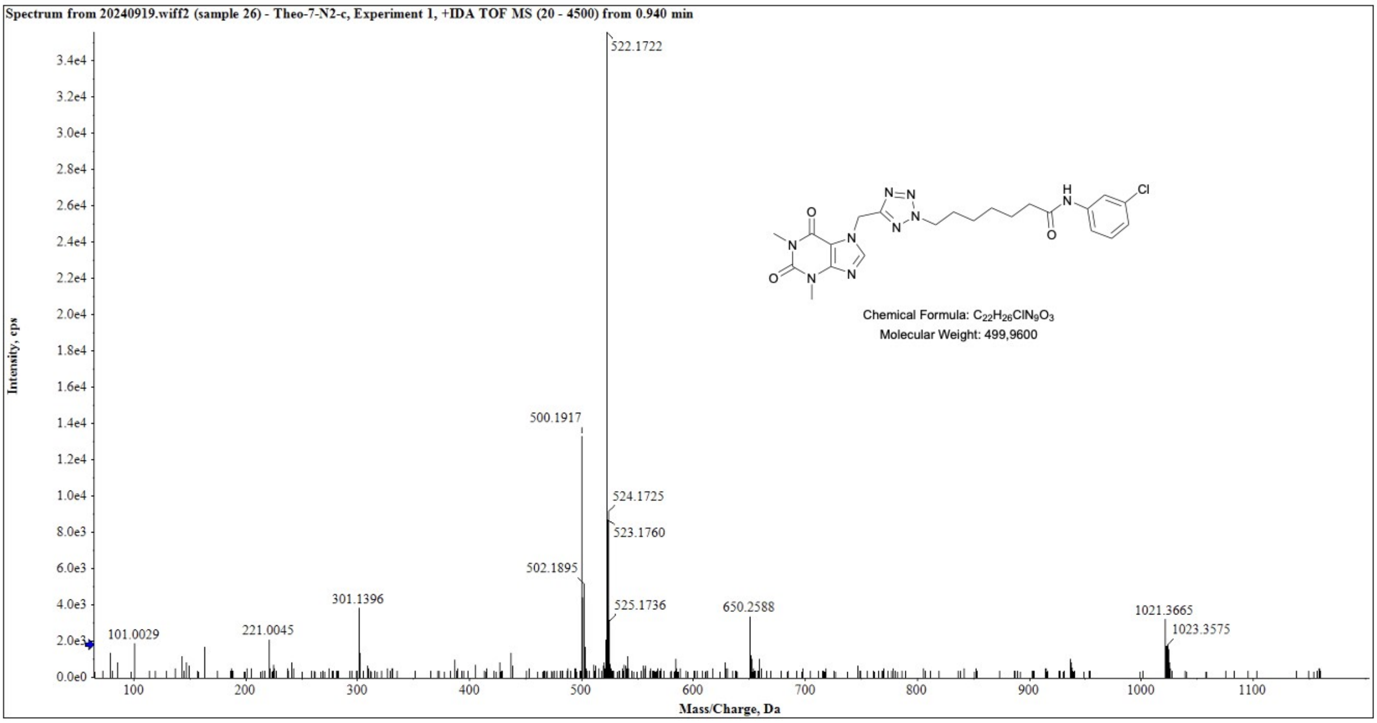

^{1}H-NMR of compound 21

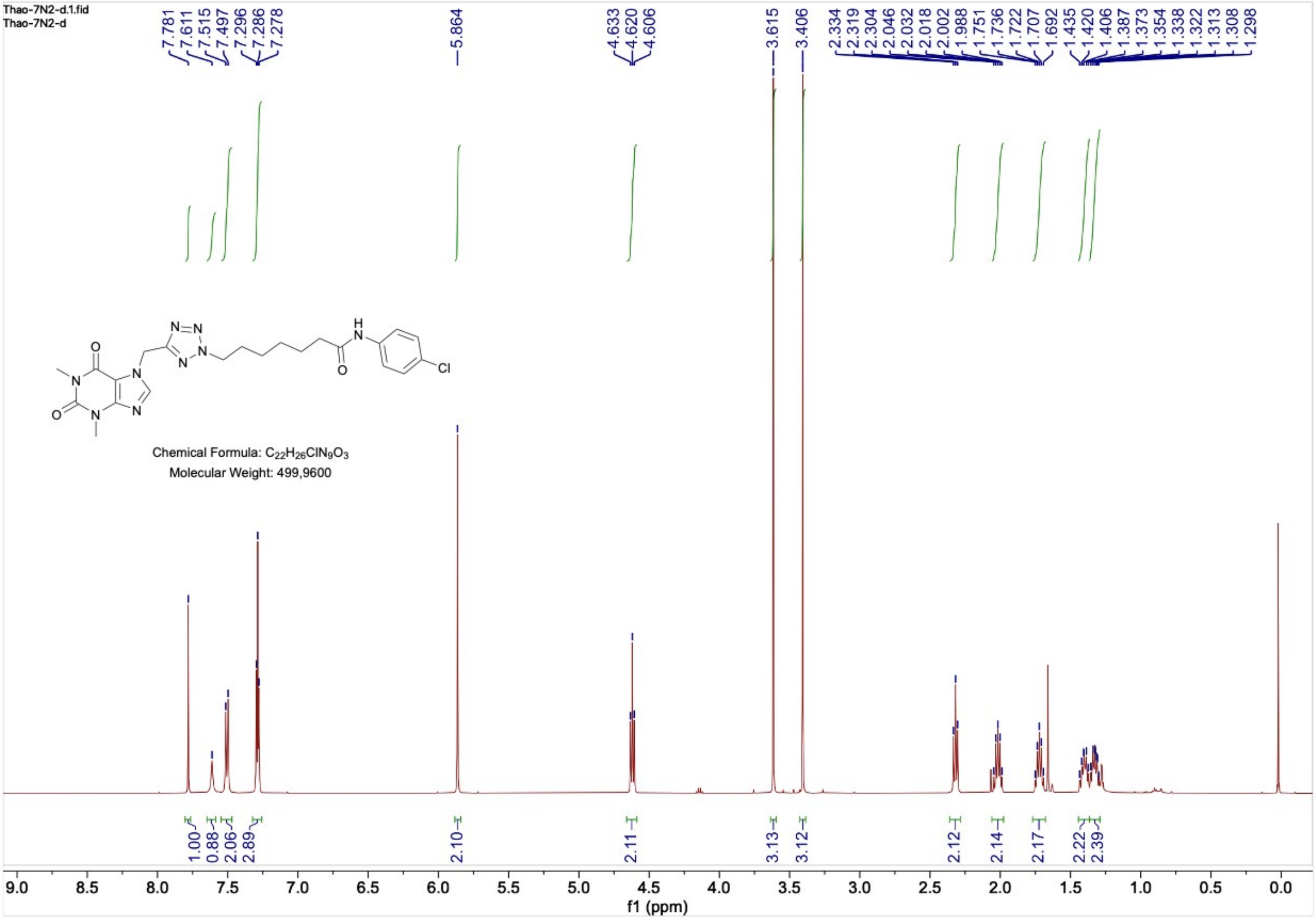

^{13}C-NMR of compound 21

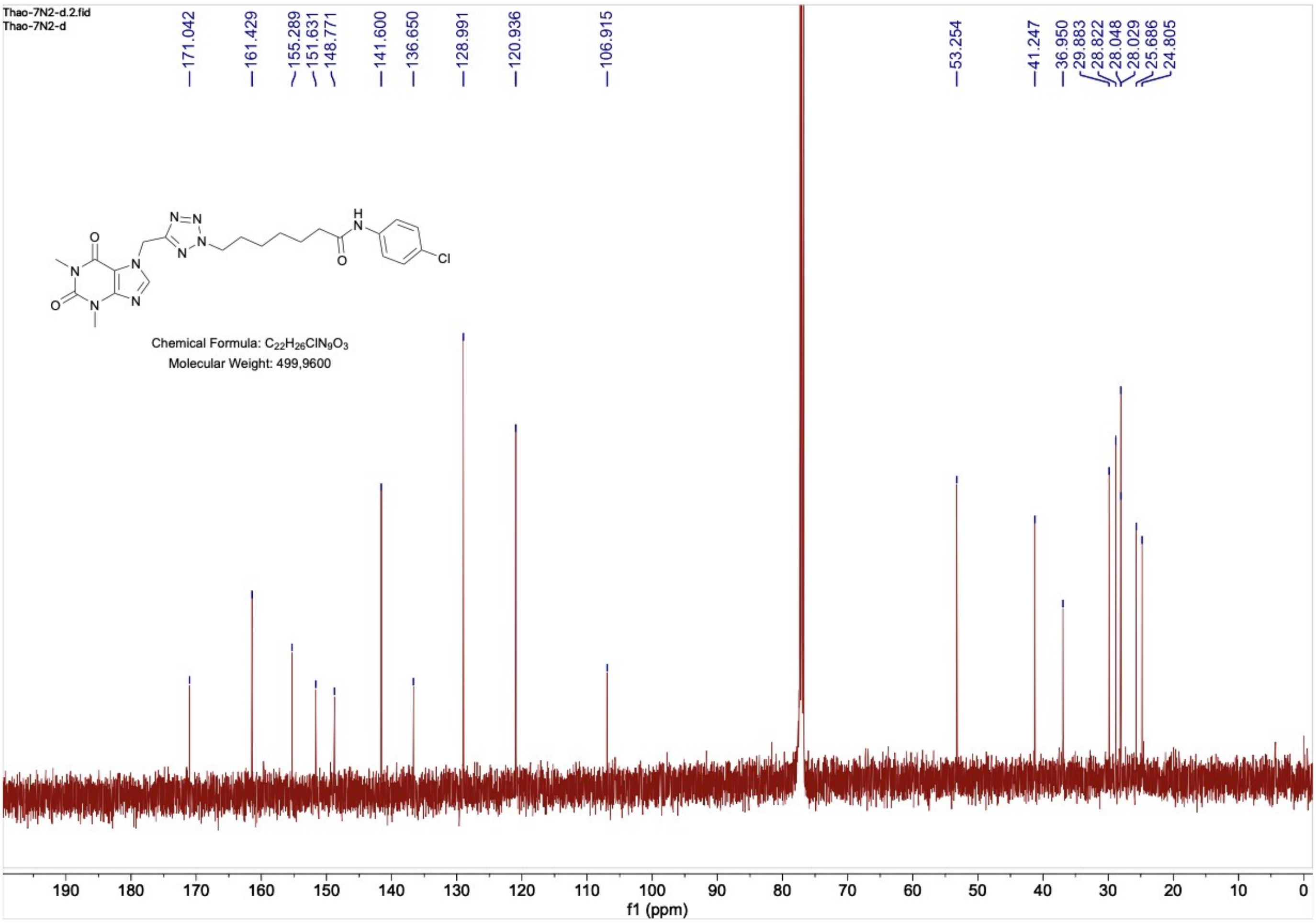

HR-MS of compound 21

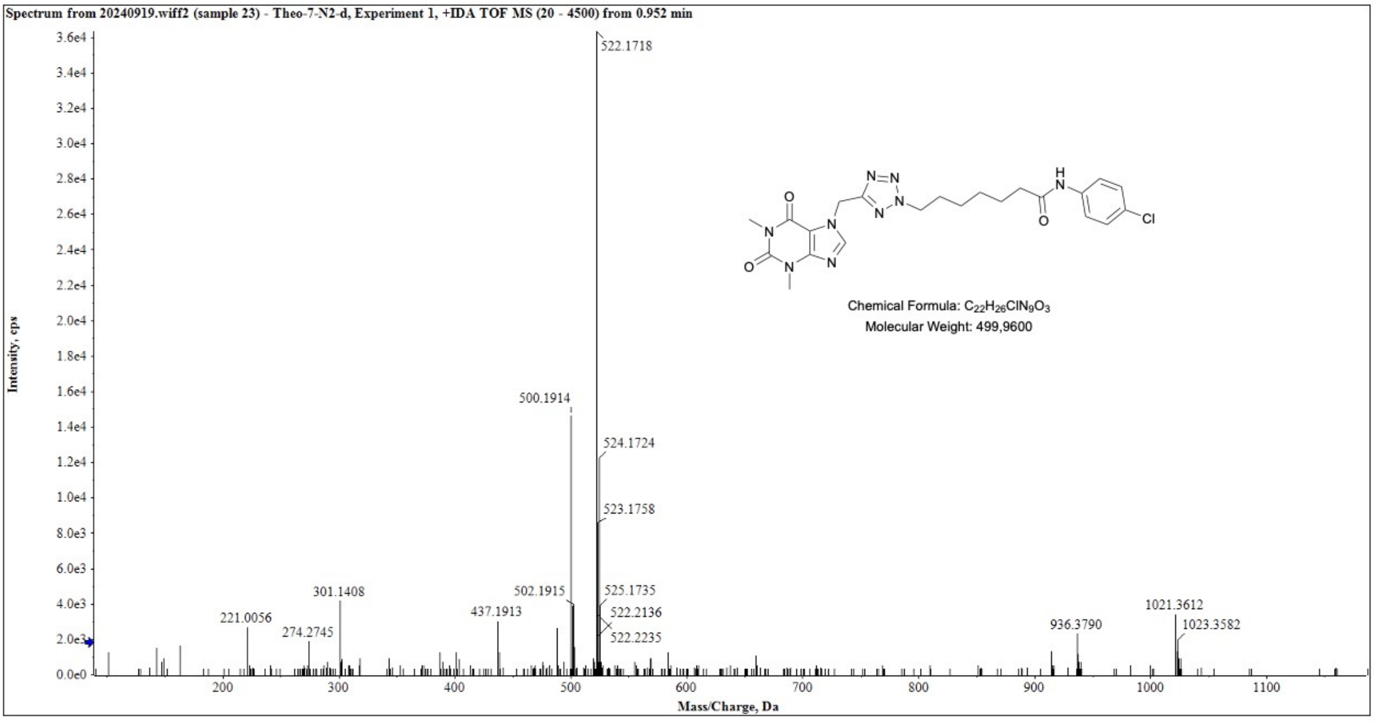

^{1}H-NMR of compound 22

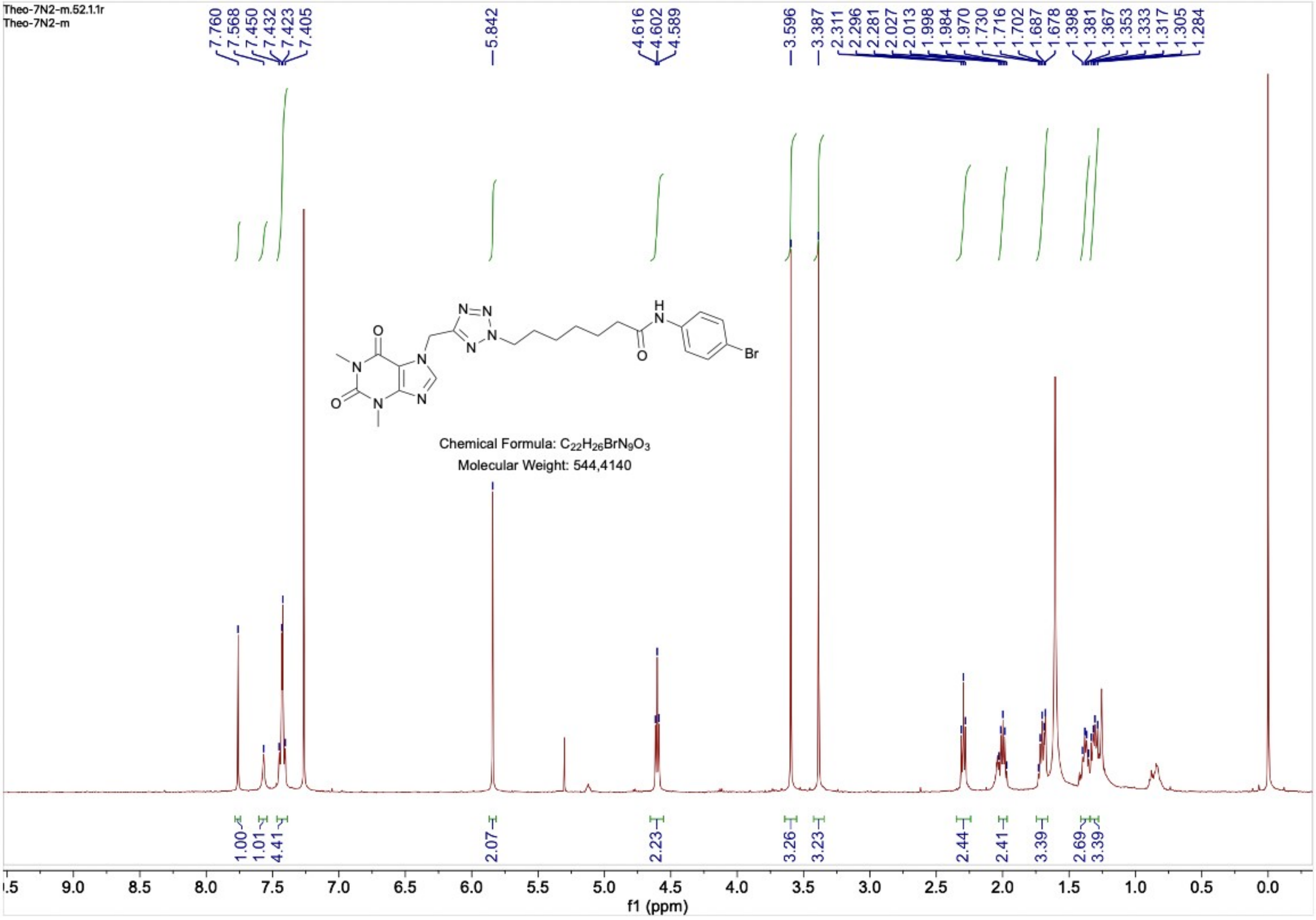

^{13}C-NMR of compound 22

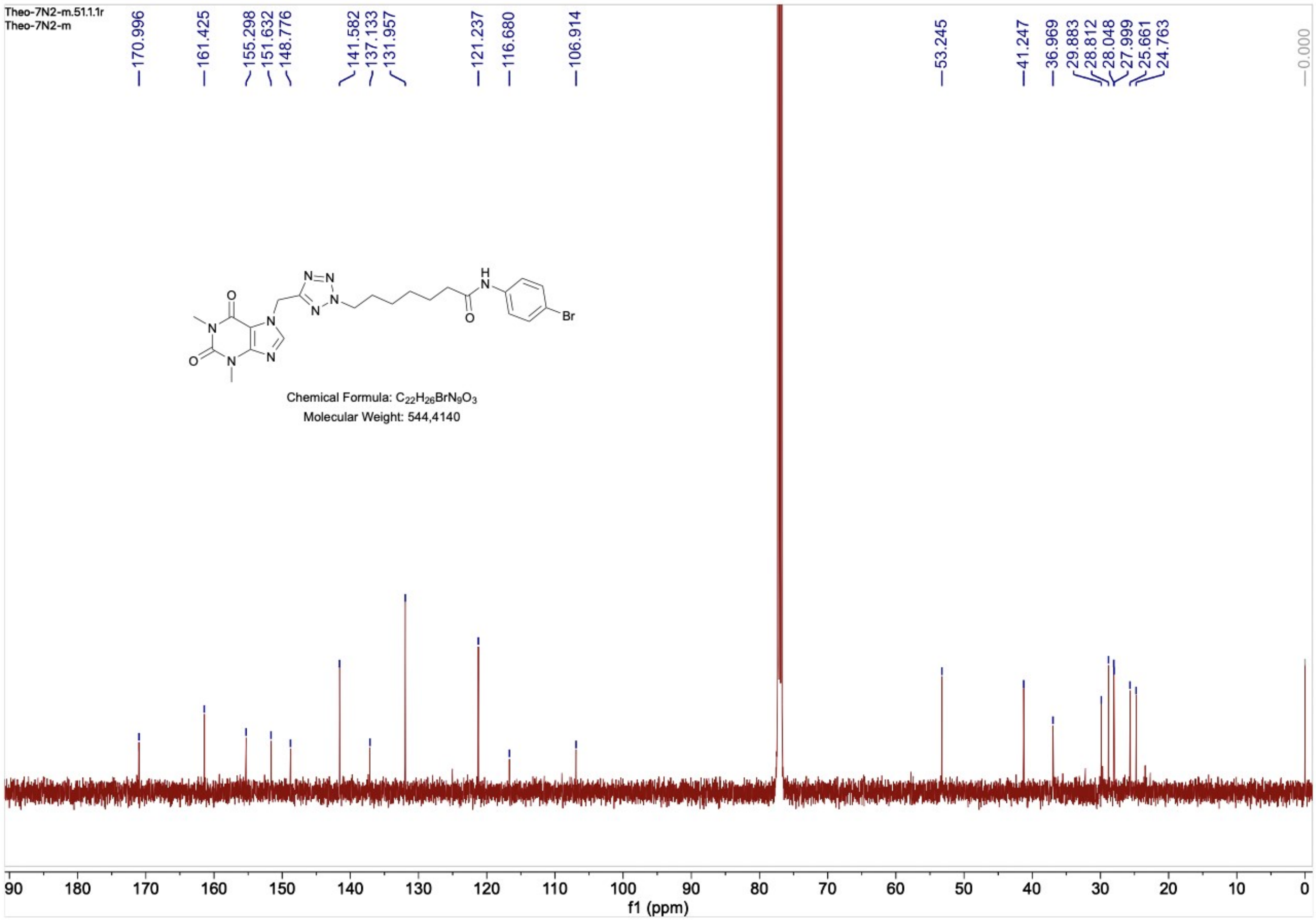

HR-MS of compound 22

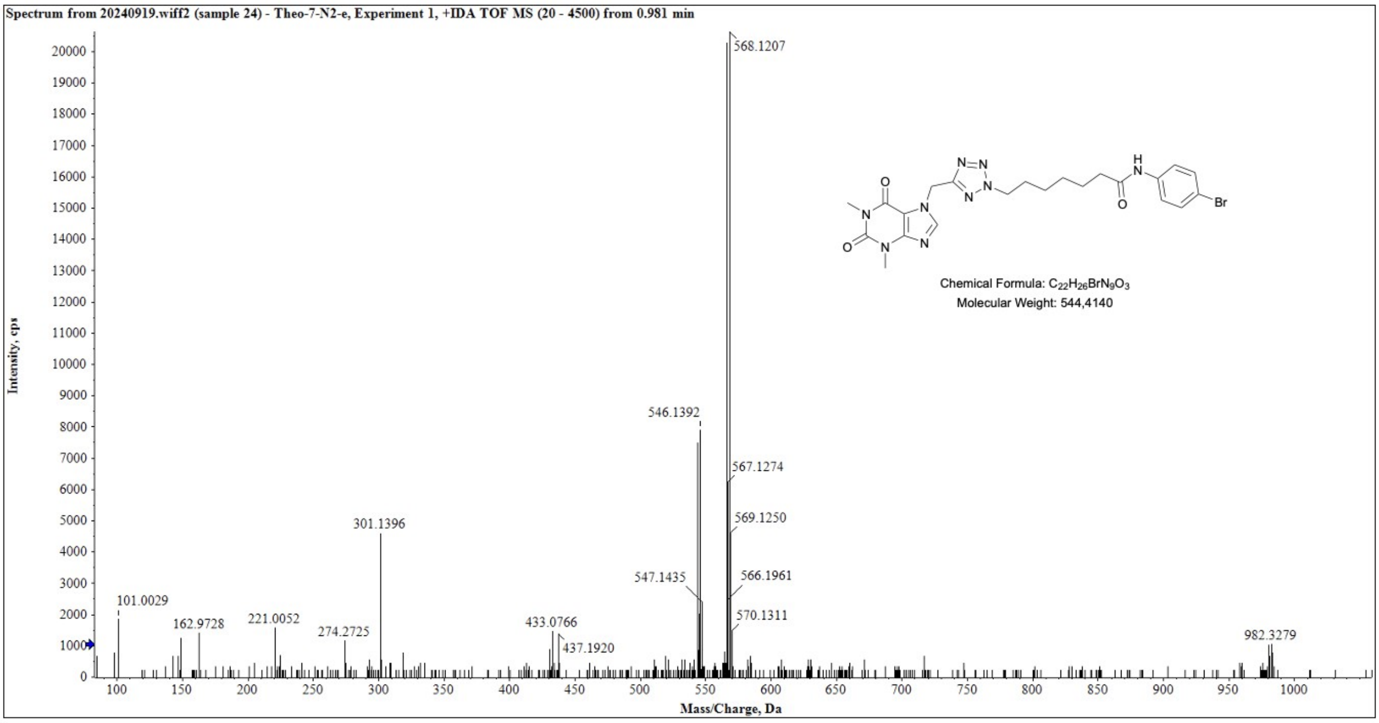

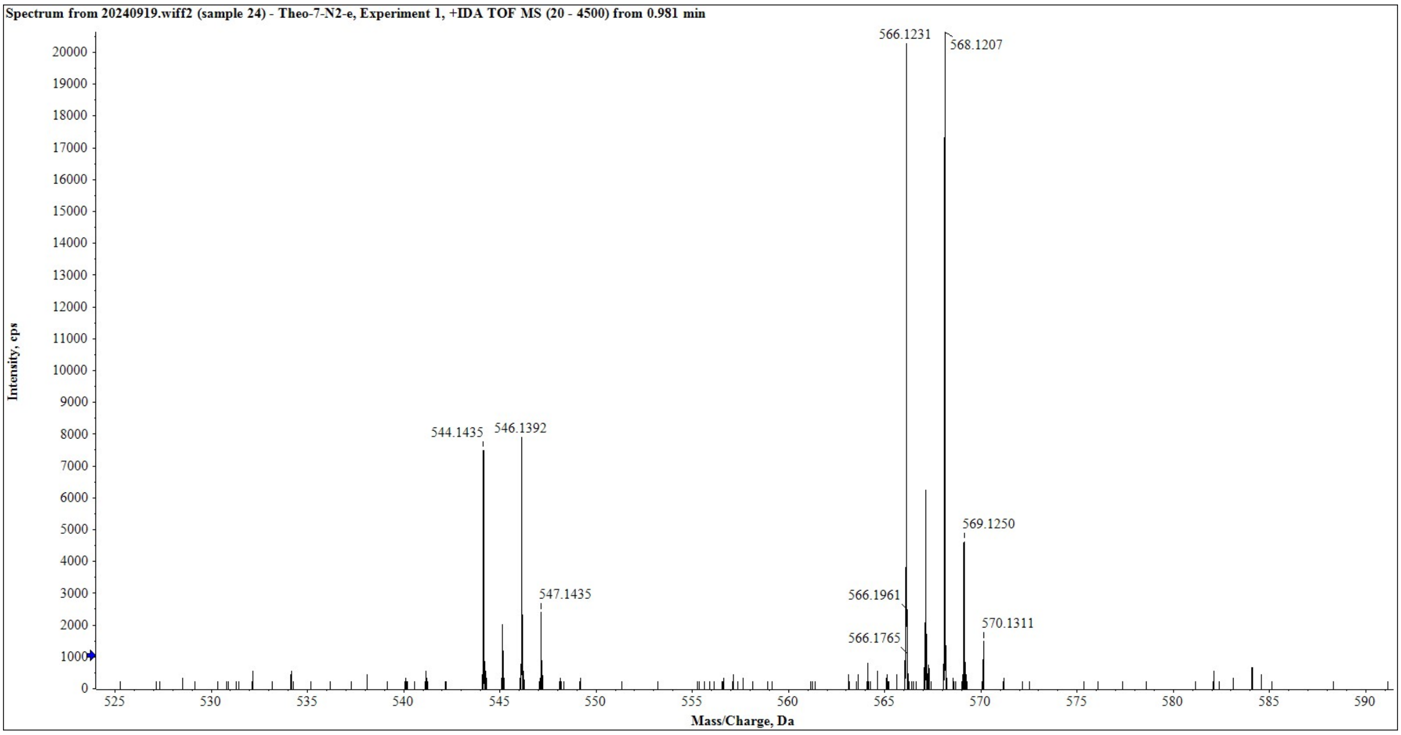

^{1}H-NMR of compound 23

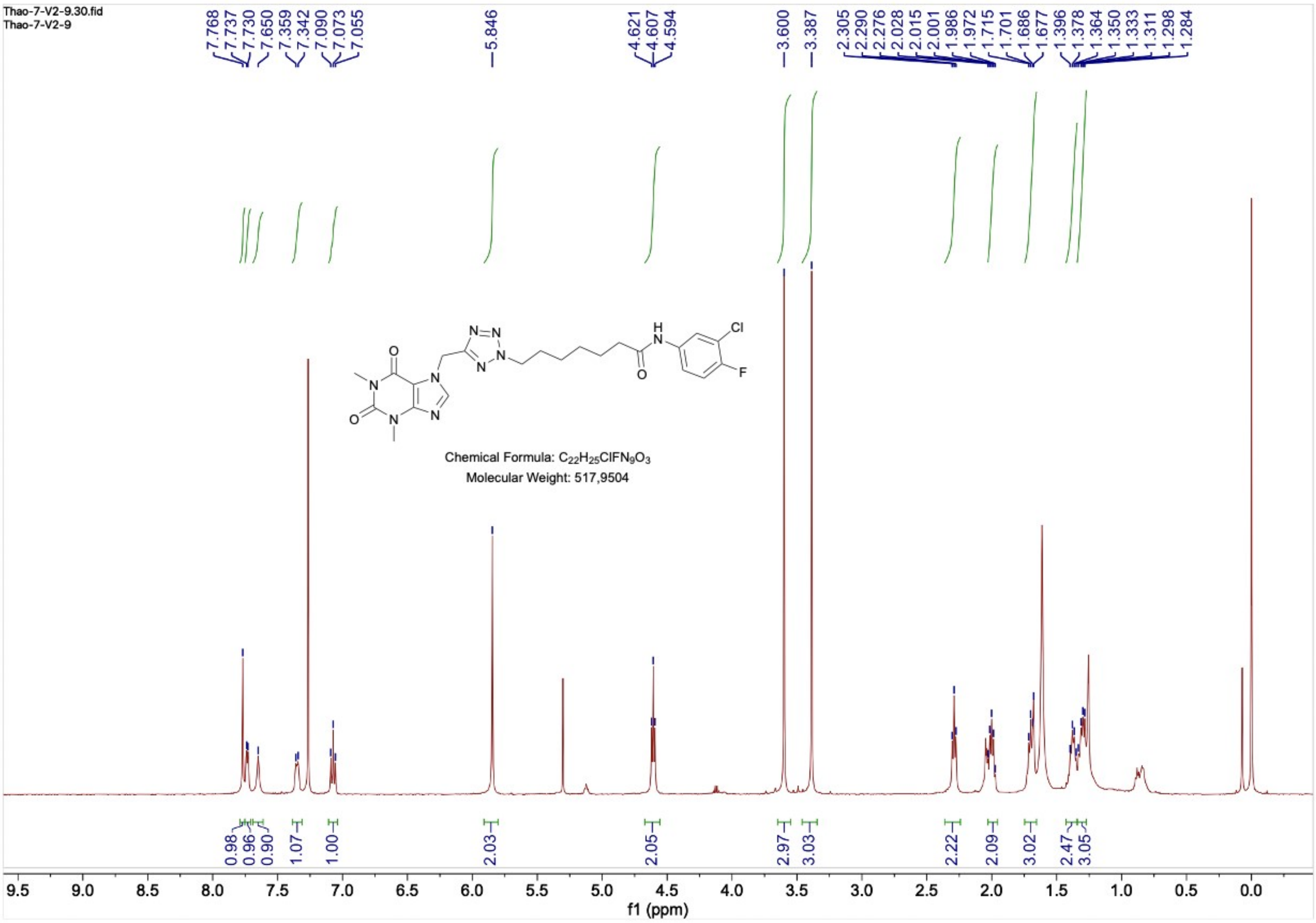

^{13}C-NMR of compound 23

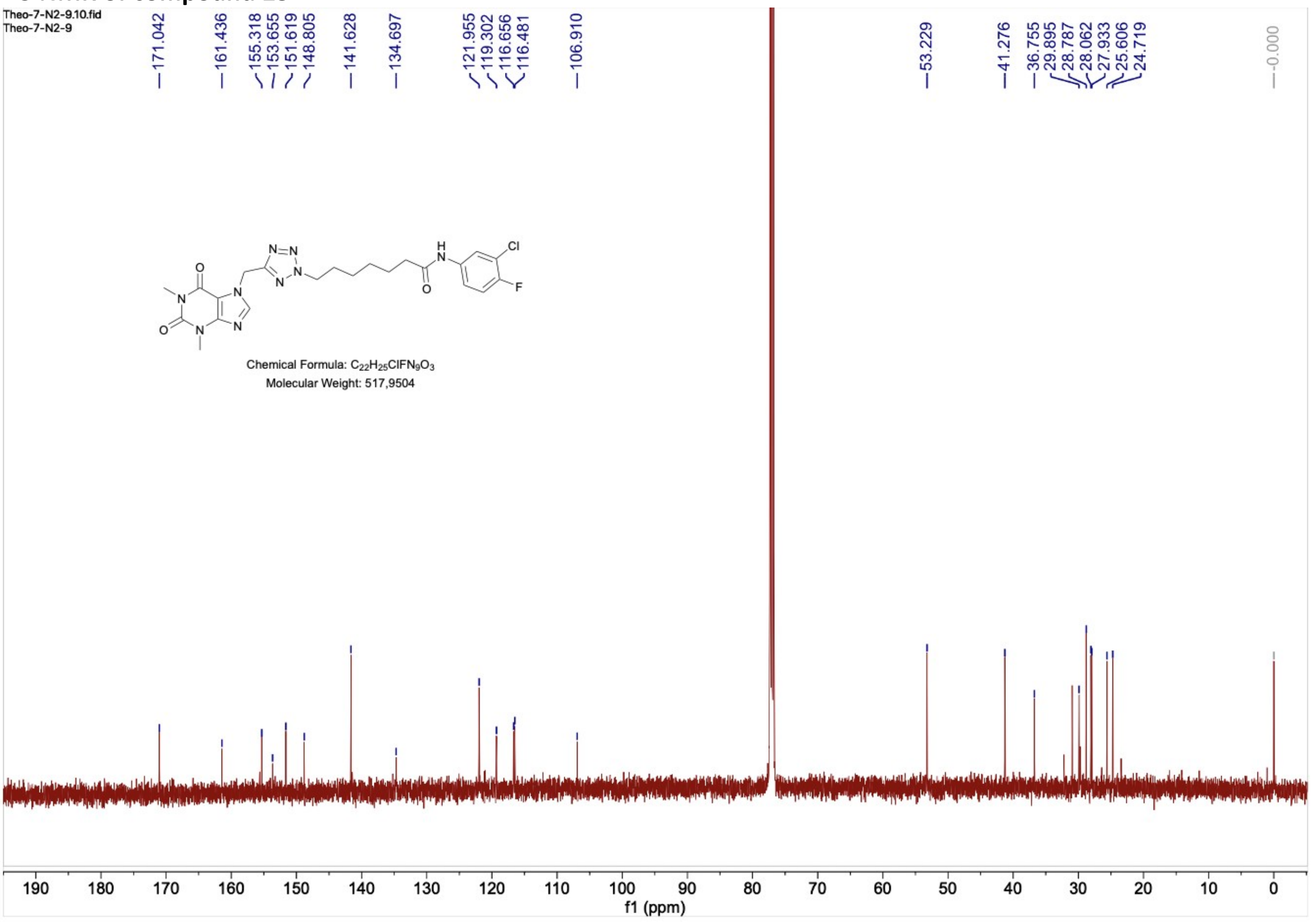

HR-MS of compound 23

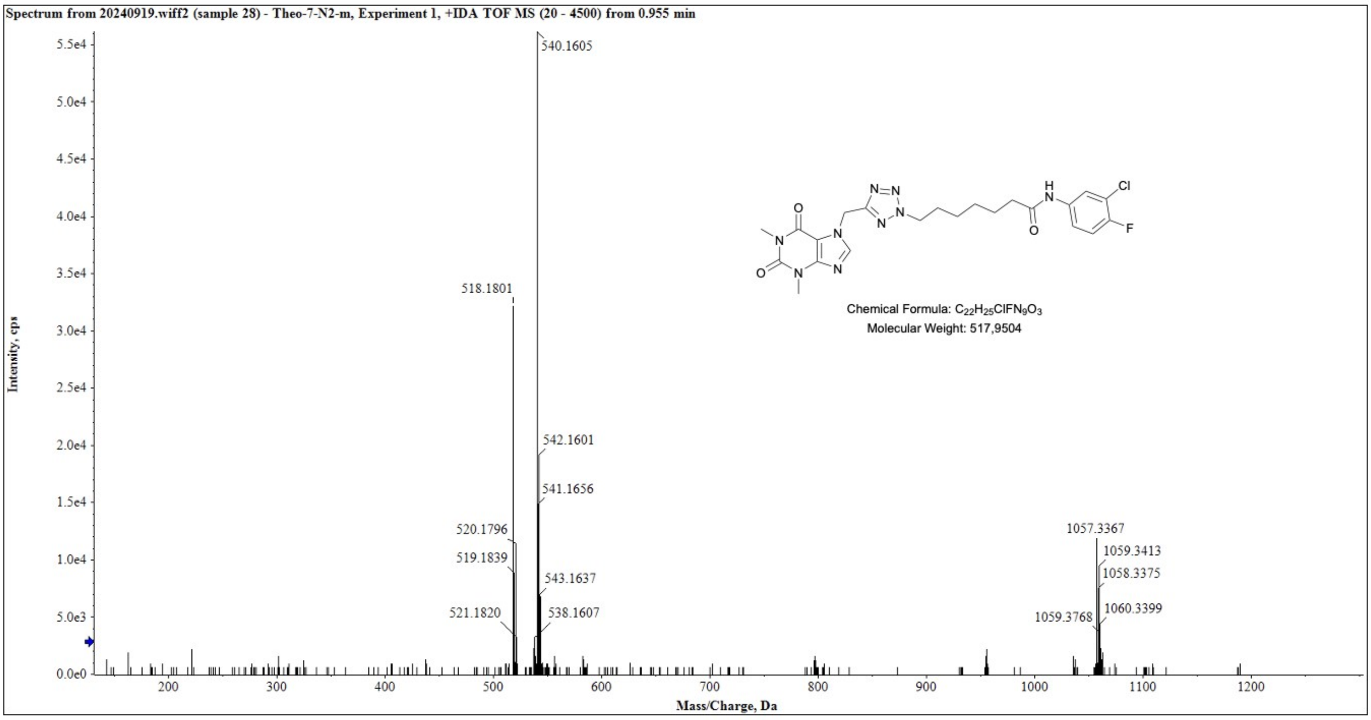

^{1}H-NMR of compound 24

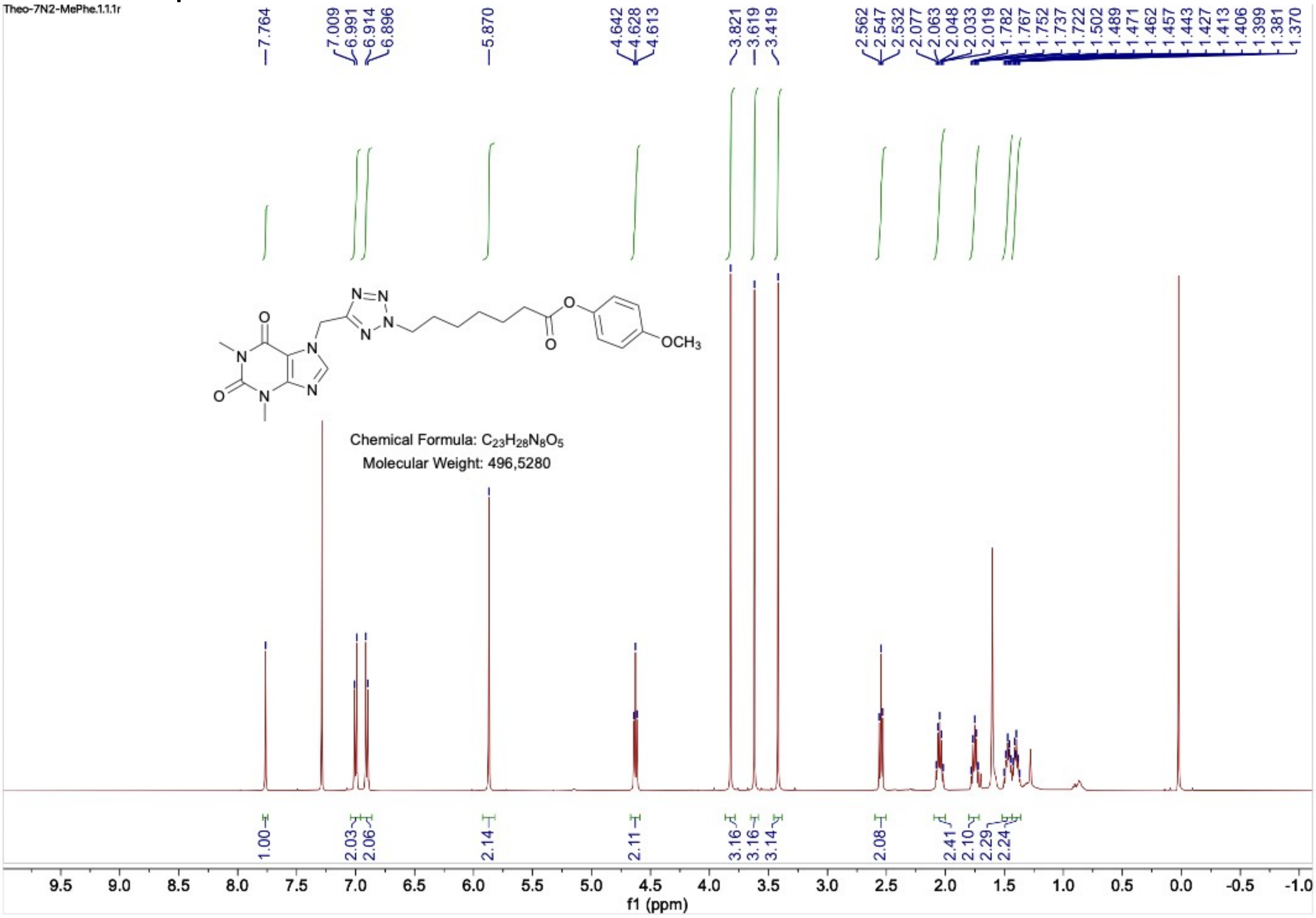

^{13}C-NMR of compound 24

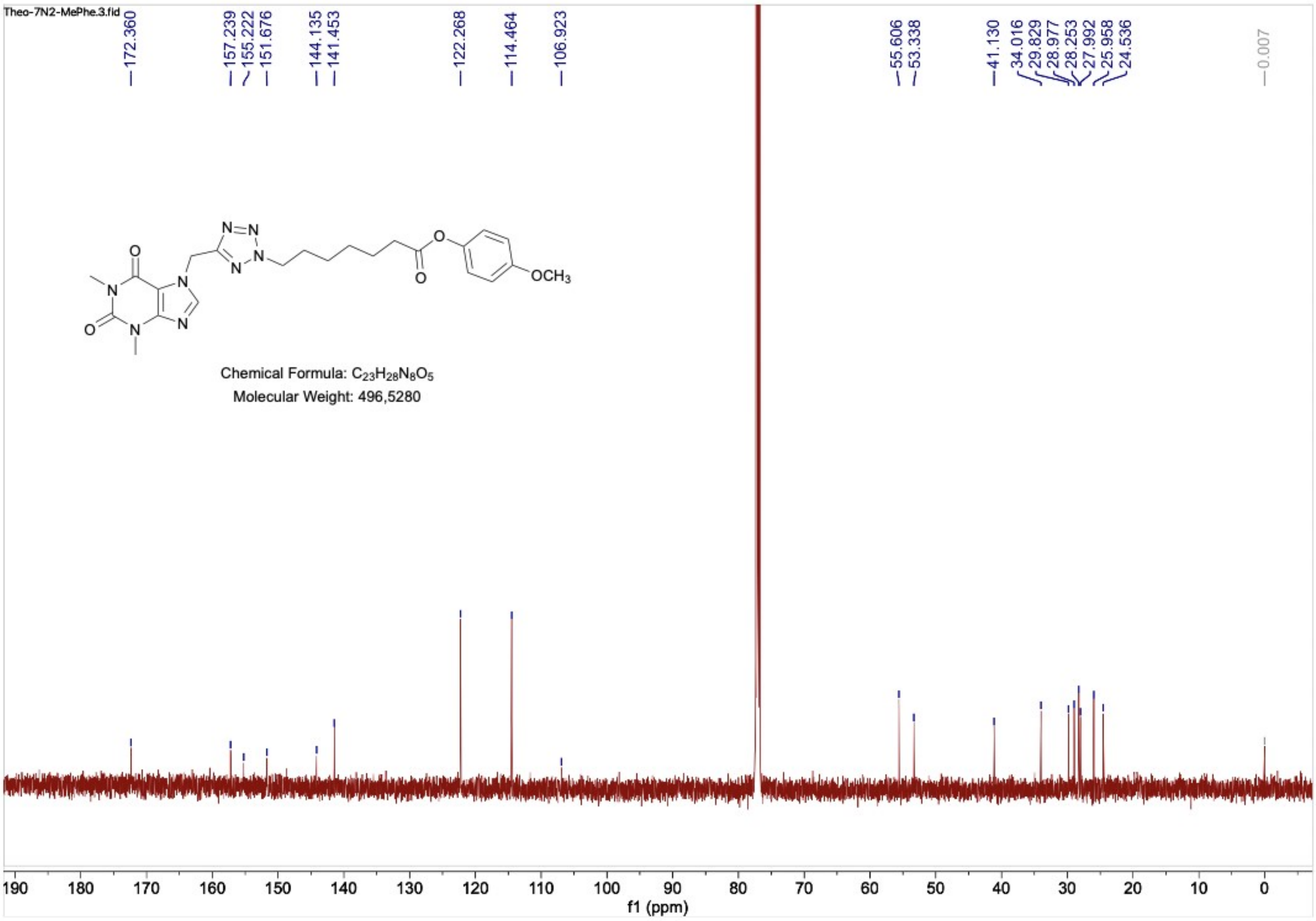

HR-MS of compound 24

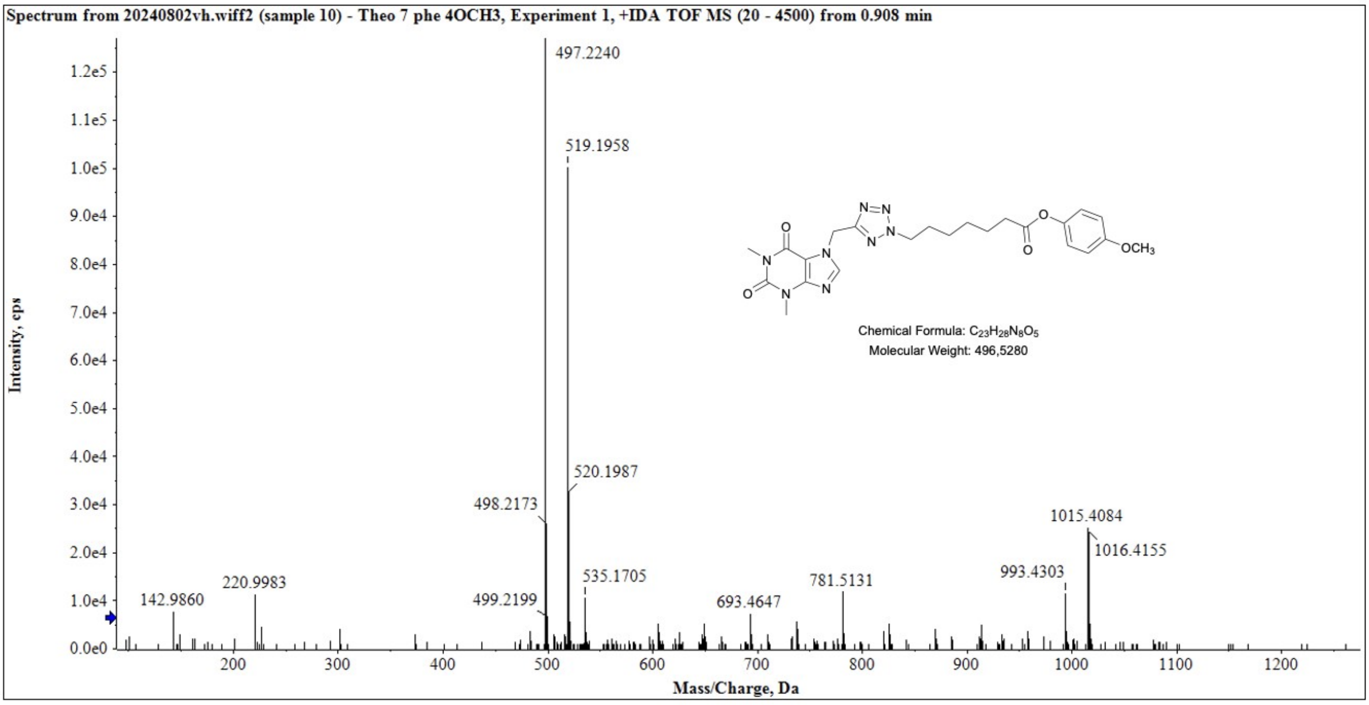

^{1}H-NMR of compound 25

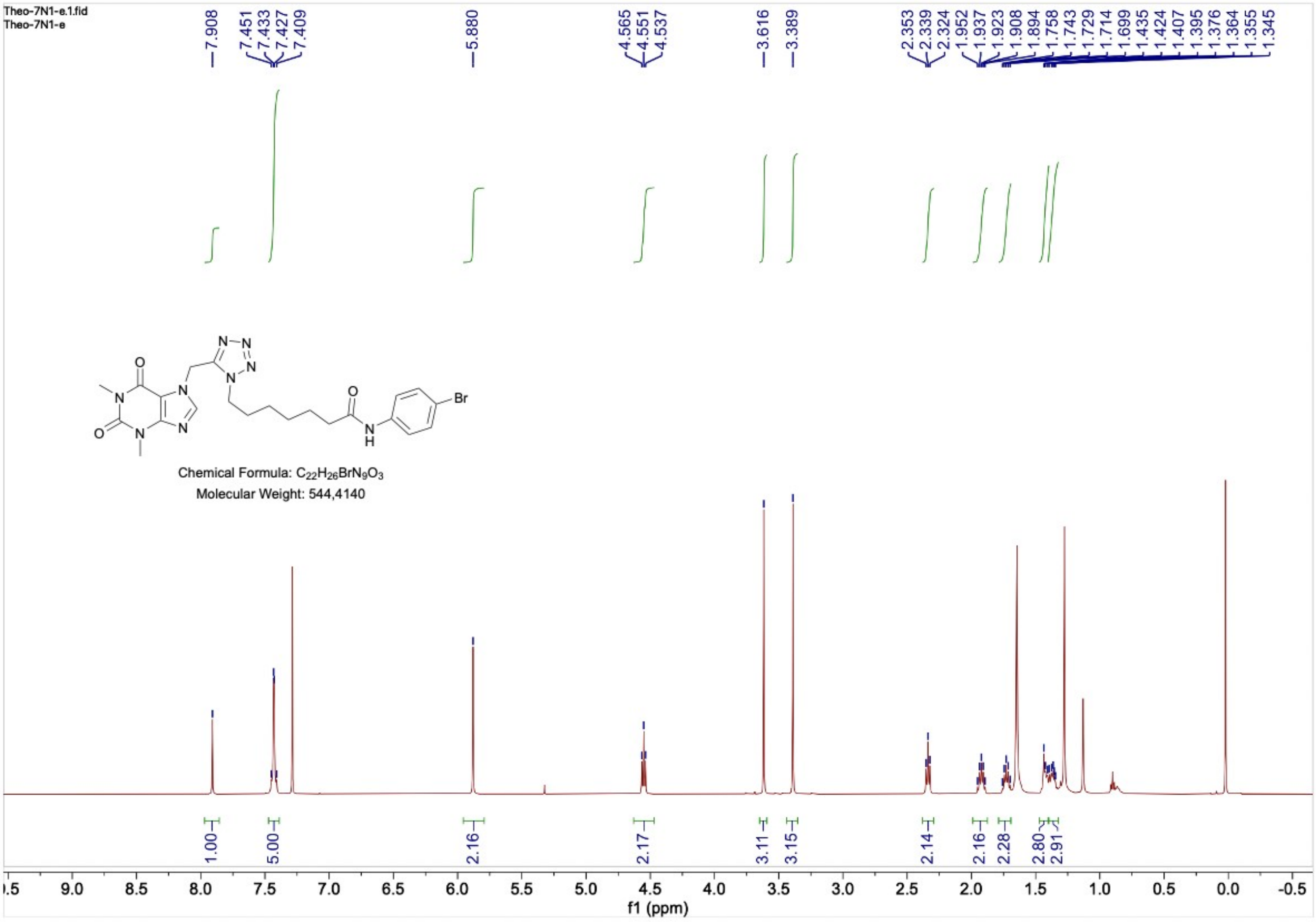

^{13}C-NMR of compound 25

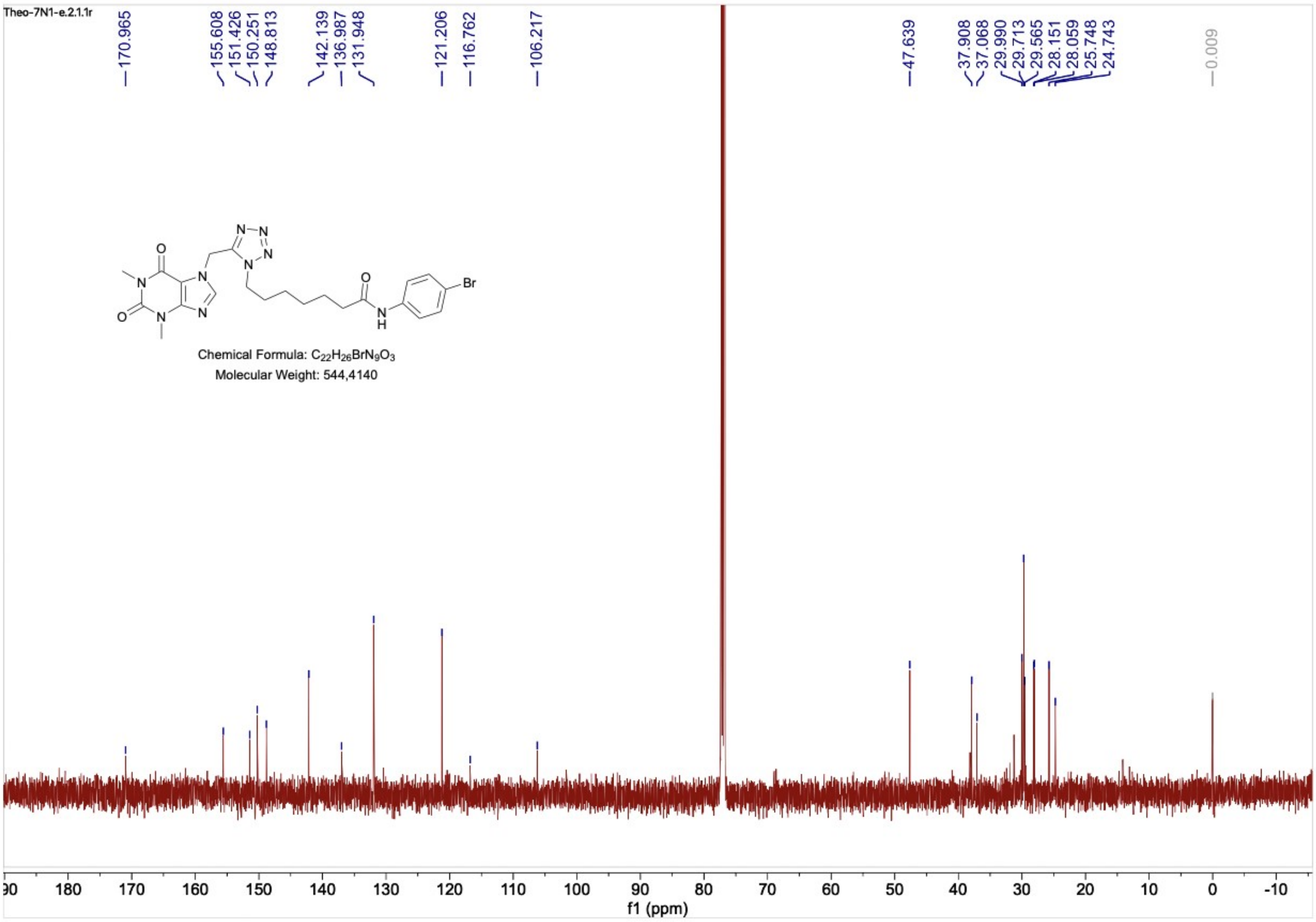

HR-MS of compound 25

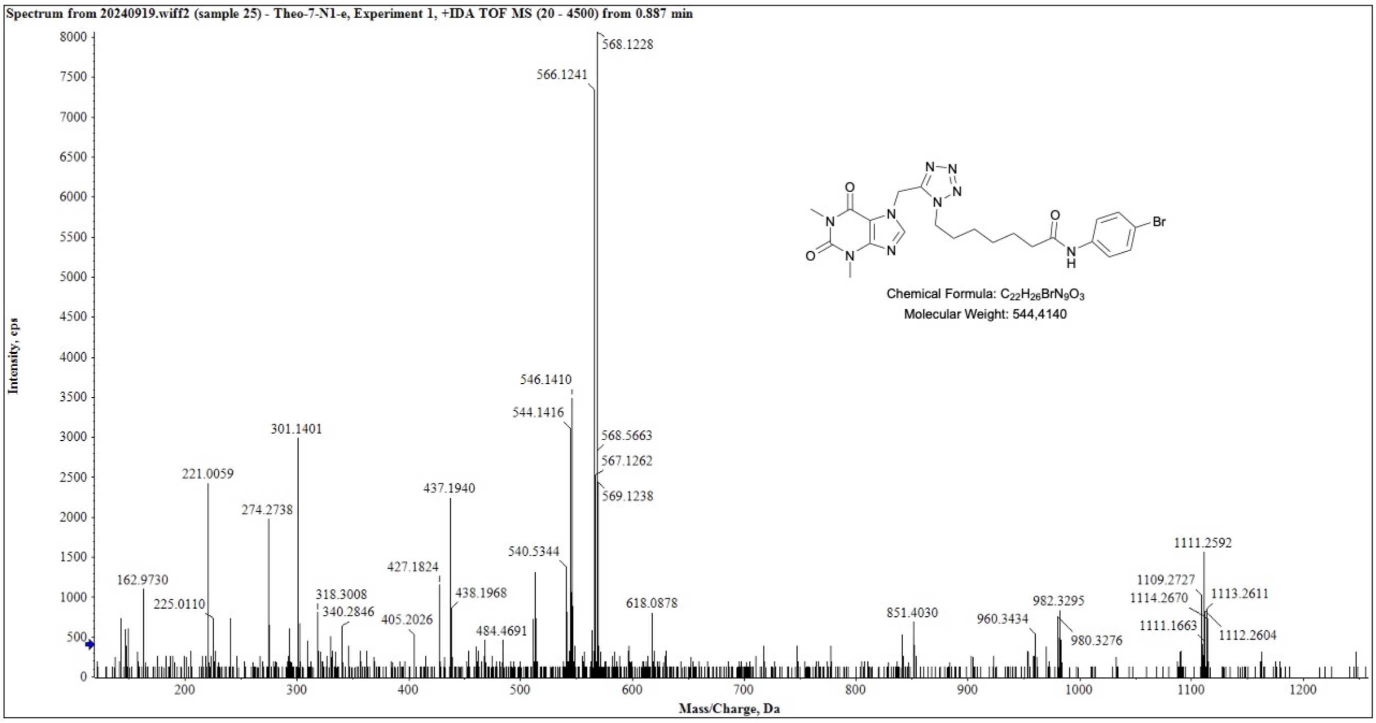

^{1}H-NMR of compound 26

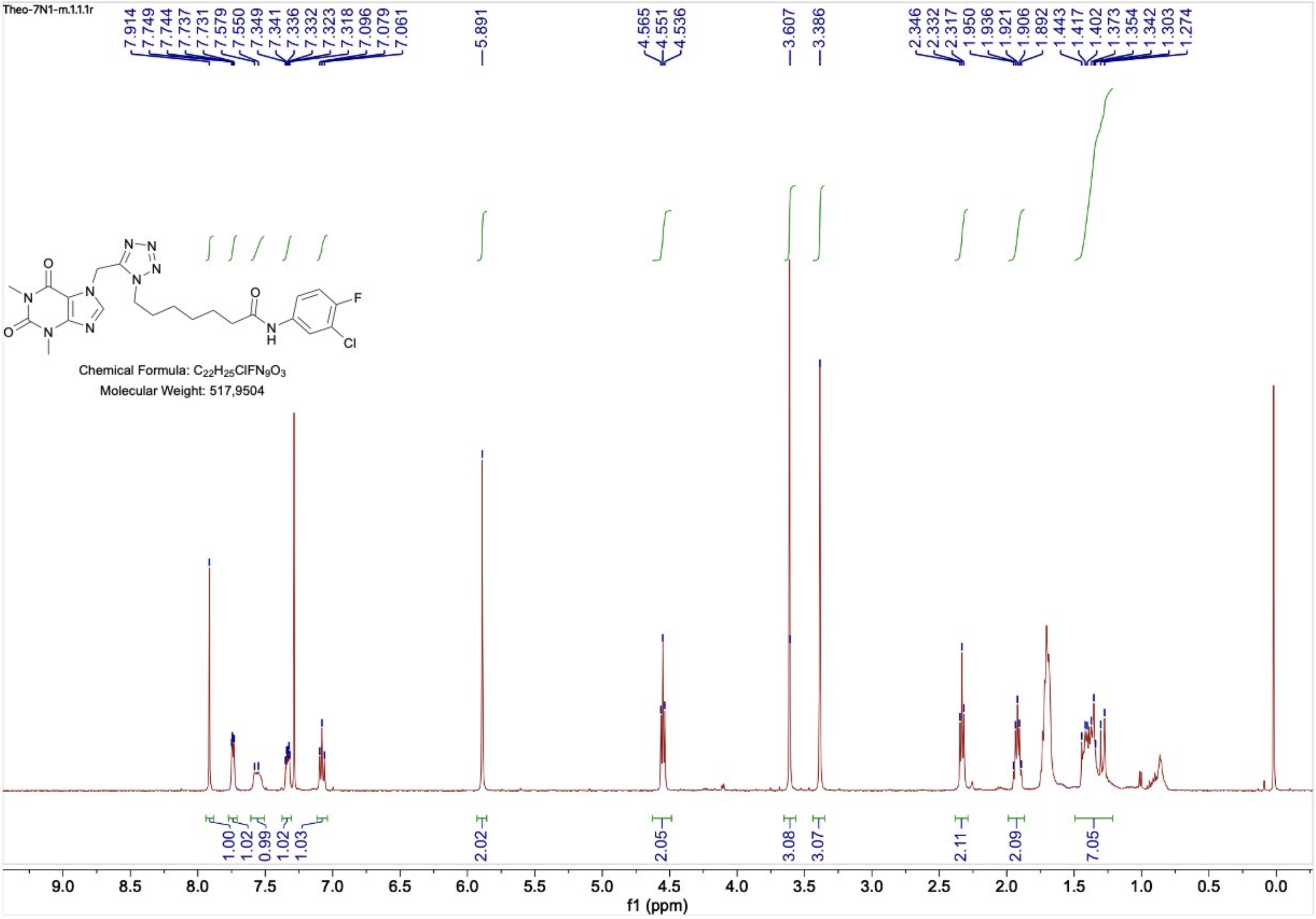

^{13}C-NMR of compound 26

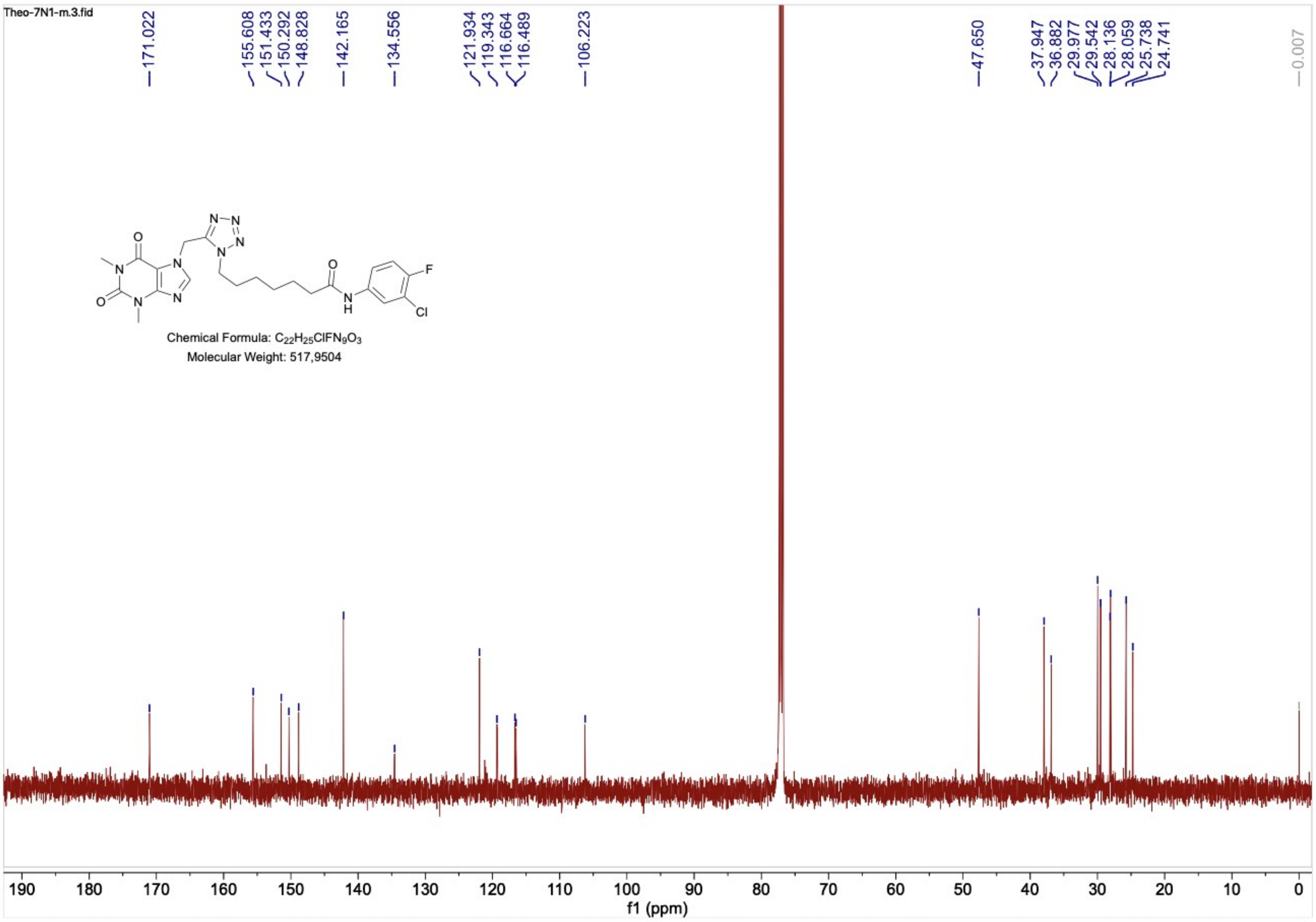

HR-MS of compound 26

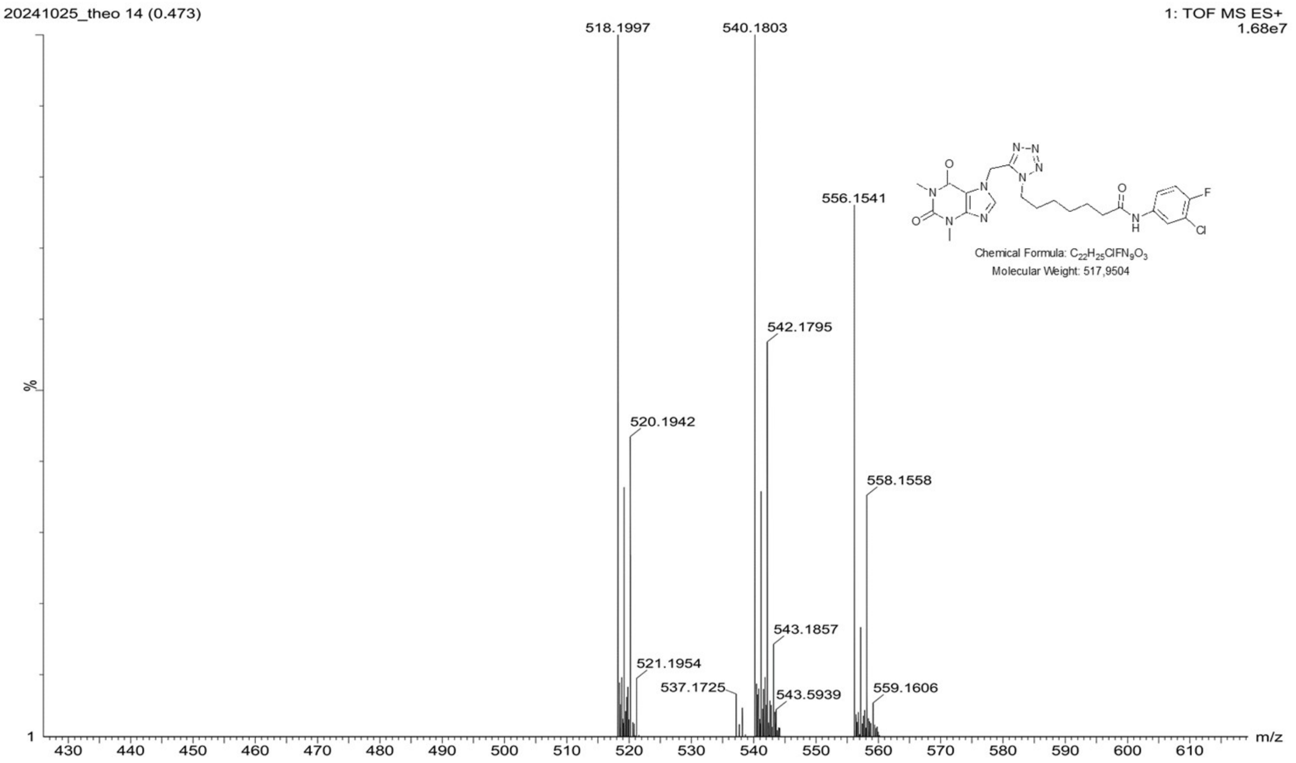

^{1}H-NMR of compound 27

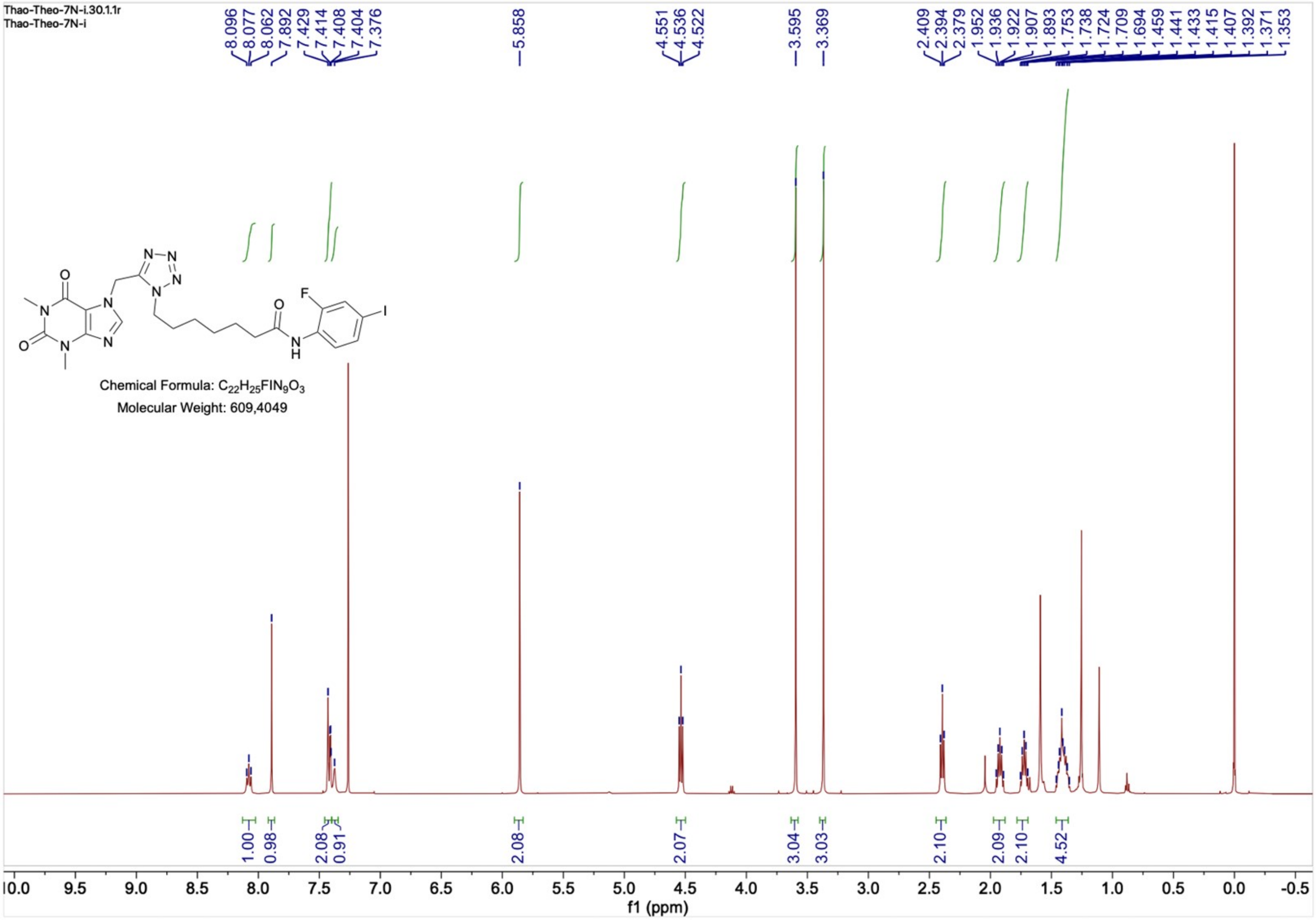

^{13}C-NMR of compound 27

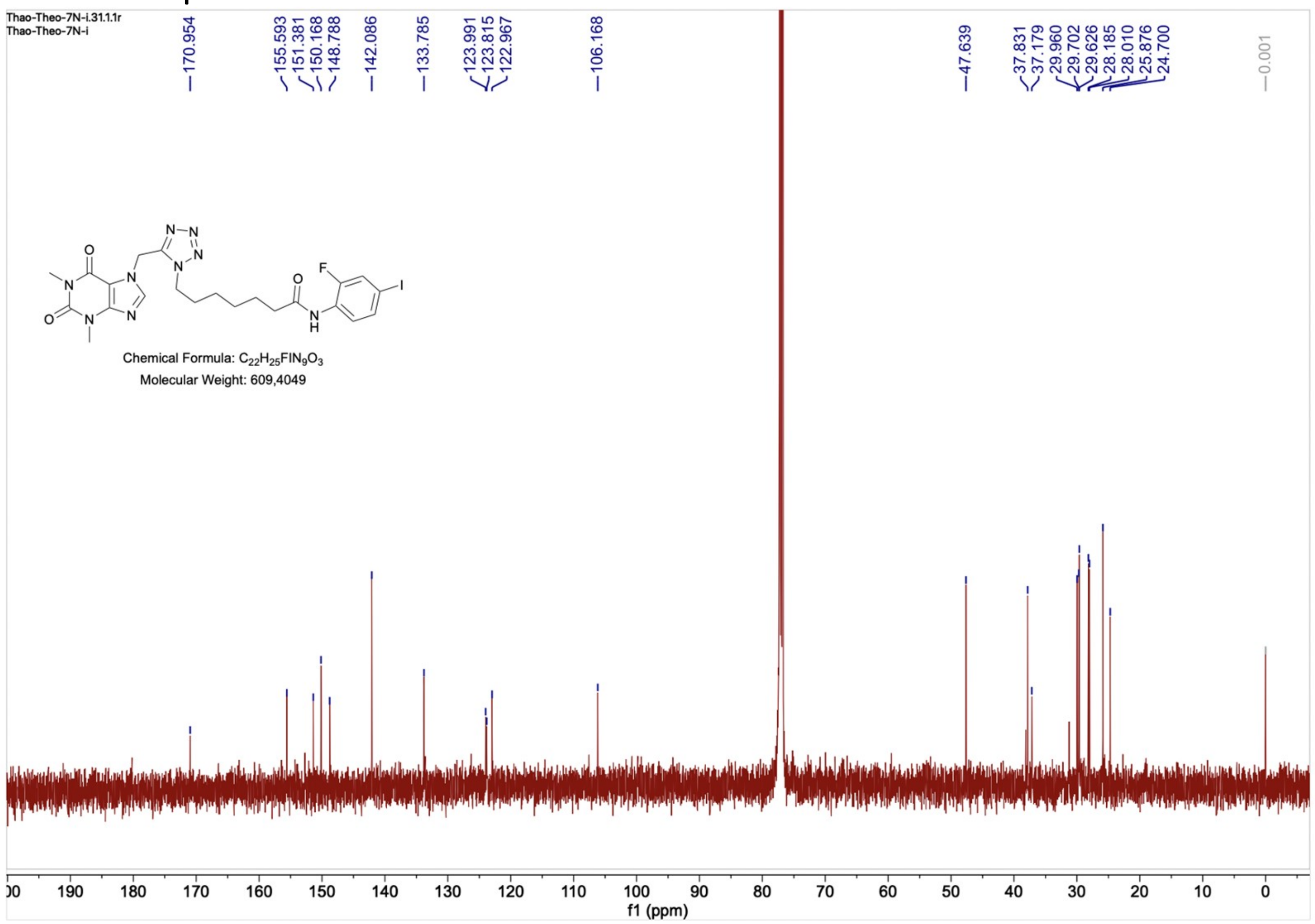

HR-MS of compound 27

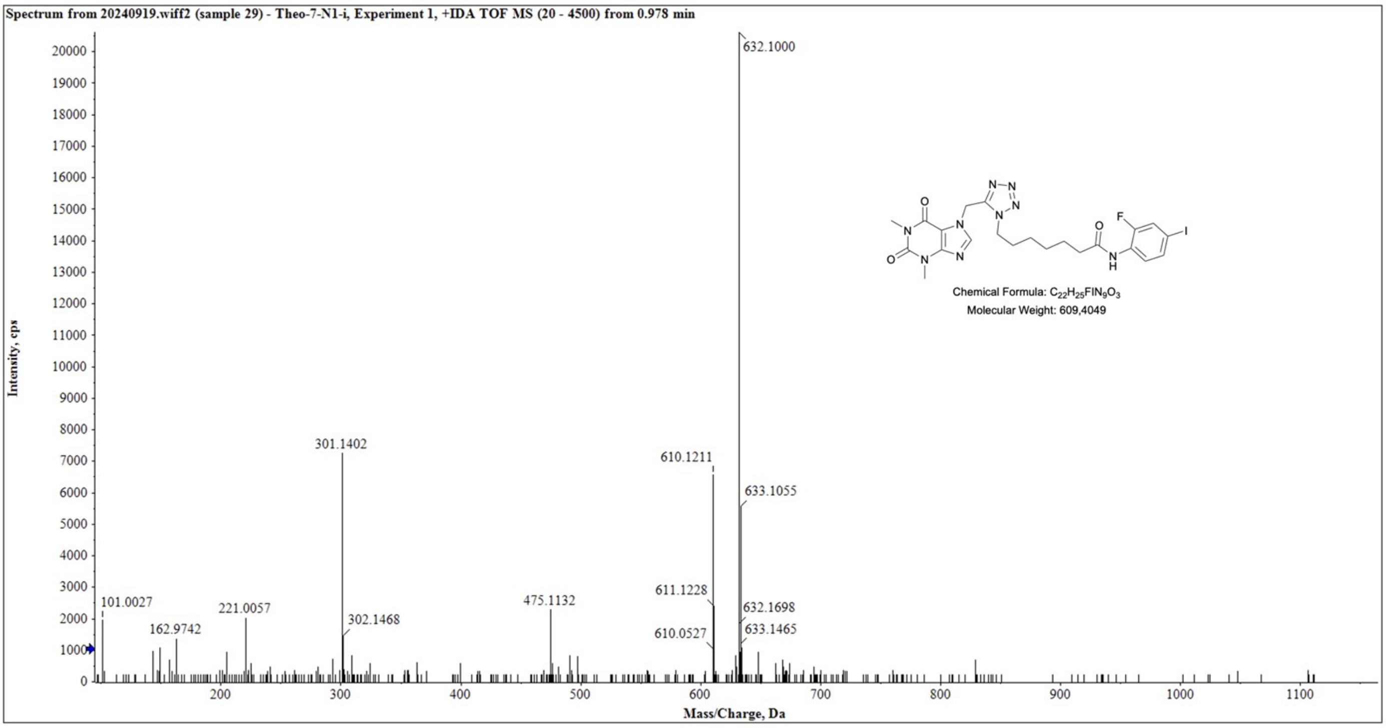

Table S2: 2D Chemical Structures and 3D diagrams representing the binding pose and protein-ligand interactions of six compounds **12, 15, 18, 22, 25, 26**

Nº.	Code	2D Structure	3D Structure
1	12	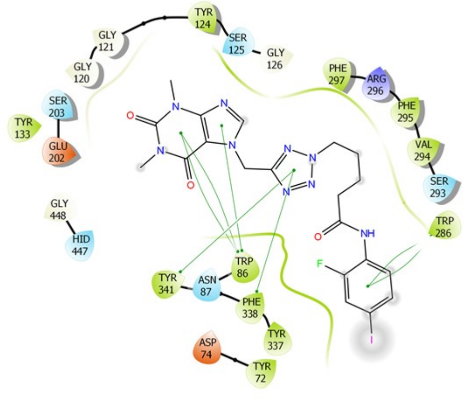	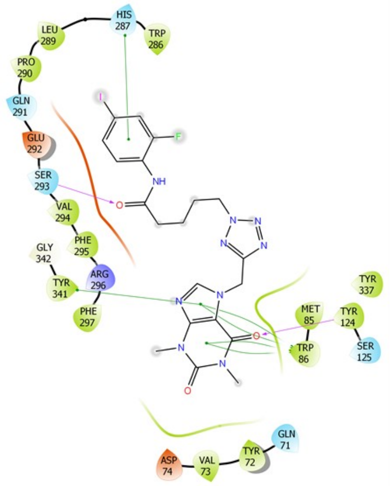
2	15	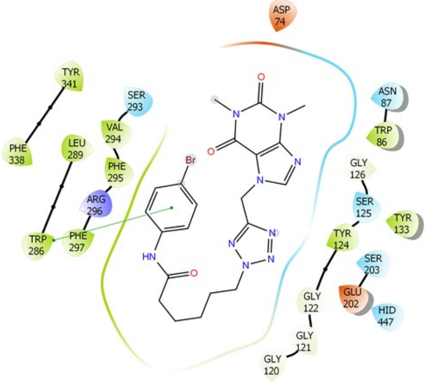	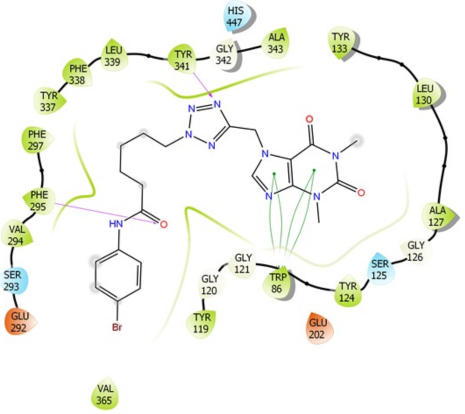
3	18	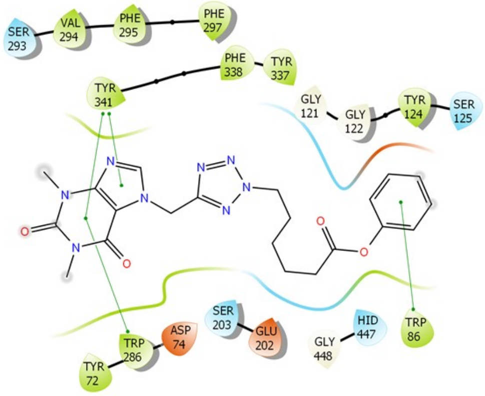	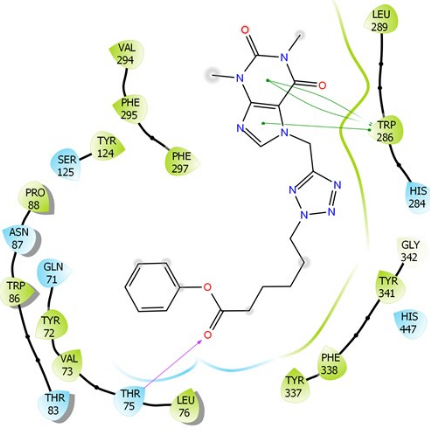

**4**	**22**	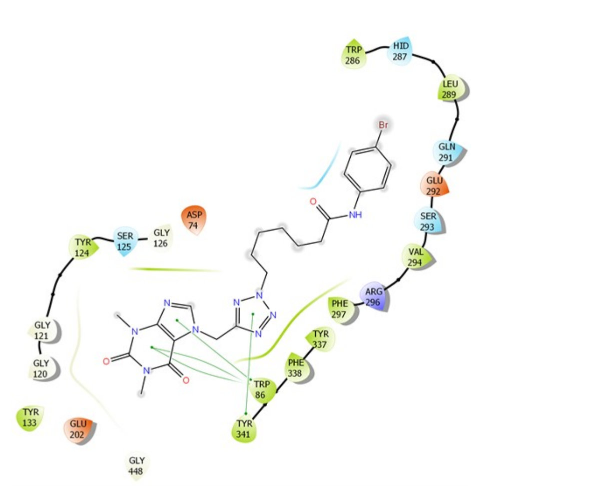TRP 286 HID 287 LEU 289 GLN 291 GLU 292 SER 293 VAL 294 ARG 296 PHE 297 TYR 337 PHE 338 TRP 86 TYR 341 ASP 74 GLY 126 SER 125 TYR 124 GLY 121 GLY 120 TYR 133 GLU 202 GLY 448 Br NH	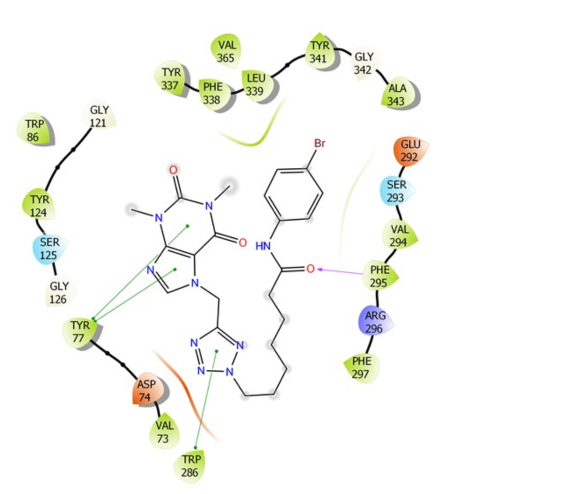VAL 365 TYR 341 GLY 342 ALA 343 TYR 337 PHE 338 LEU 339 TRP 86 GLY 121 GLU 292 SER 293 VAL 294 PHE 295 ARG 296 PHE 297 TYR 124 SER 125 GLY 126 TYR 77 ASP 74 VAL 73 TRP 286 Br HN
**5**	**25**	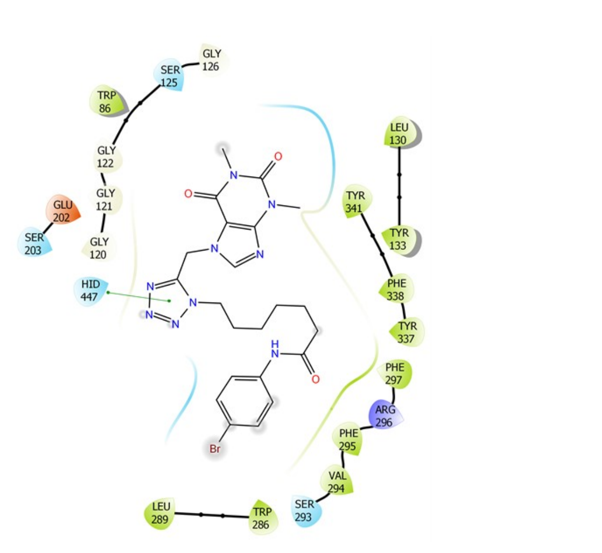GLY 126 SER 125 TRP 86 GLY 122 GLY 121 GLY 120 GLU 202 SER 203 HID 447 LEU 130 TYR 341 TYR 133 PHE 338 TYR 337 PHE 297 ARG 296 PHE 295 VAL 294 SER 293 LEU 289 TRP 286 Br	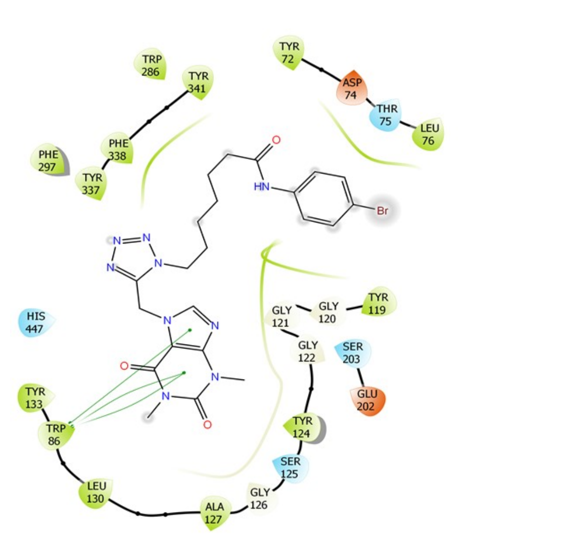TRP 286 TYR 341 TYR 72 ASP 74 THR 75 LEU 76 PHE 297 PHE 338 TYR 337 HN Br HIS 447 GLY 121 GLY 120 TYR 119 GLY 122 SER 203 GLU 202 TYR 133 TRP 86 TYR 124 SER 125 GLY 126 LEU 130 ALA 127
**6**	**26**	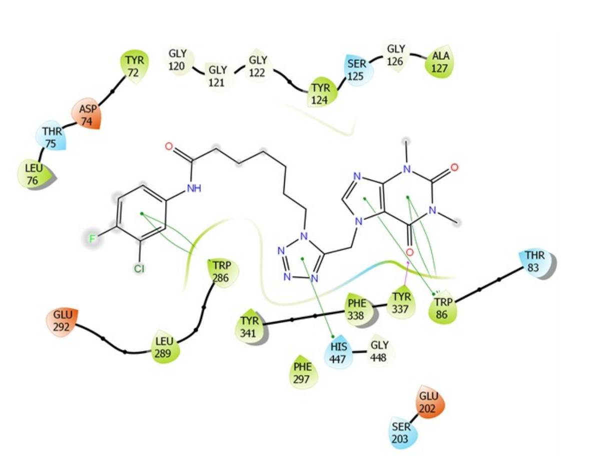TYR 72 GLY 120 GLY 121 GLY 122 TYR 124 SER 125 GLY 126 ALA 127 ASP 74 THR 75 LEU 76 NH F Cl TRP 286 THR 83 GLU 292 LEU 289 TYR 341 PHE 338 TYR 337 TRP 86 HIS 447 GLY 448 PHE 297 GLU 202 SER 203	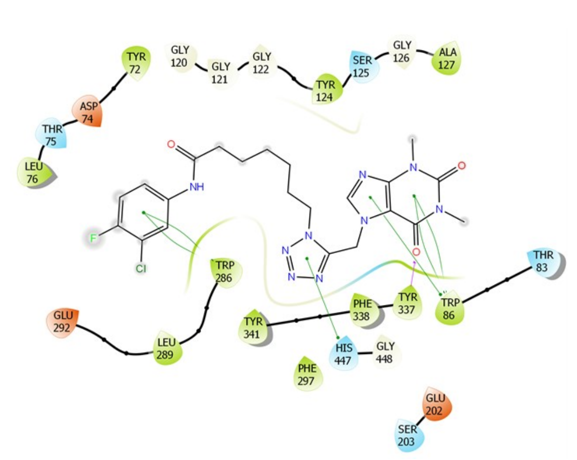TYR 72 GLY 120 GLY 121 GLY 122 TYR 124 SER 125 GLY 126 ALA 127 ASP 74 THR 75 LEU 76 NH F Cl TRP 286 THR 83 GLU 292 LEU 289 TYR 341 PHE 338 TYR 337 TRP 86 HIS 447 GLY 448 PHE 297 GLU 202 SER 203

Table S3: All-atom RMSD of available AChE complexes and inhibitor in solution systems

N⁰	Compound	Complex in Solution	Ligand in Solution
1	**12**	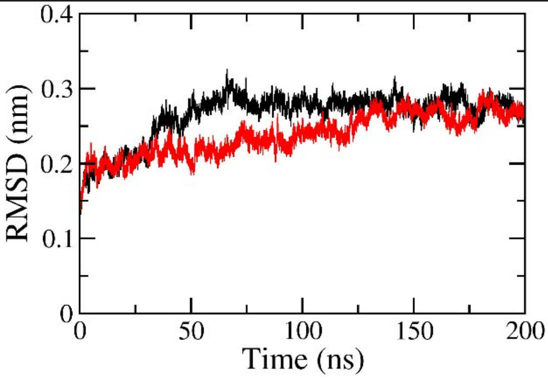	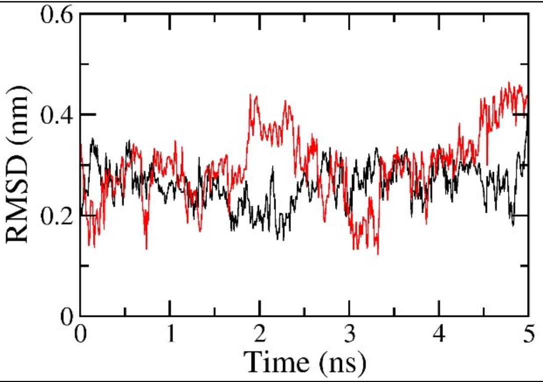
2	**15**	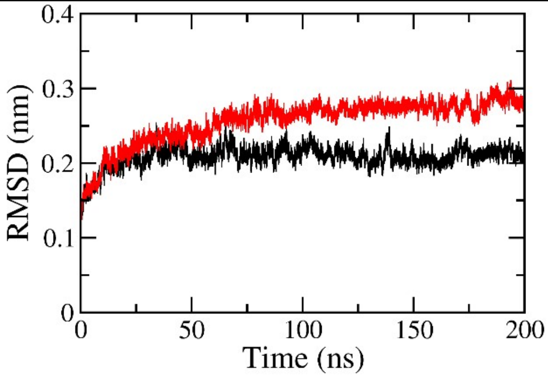	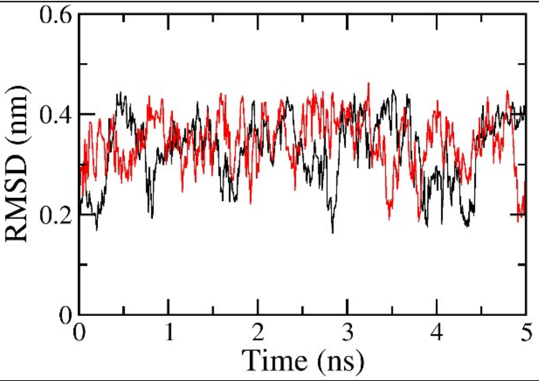
3	**18**	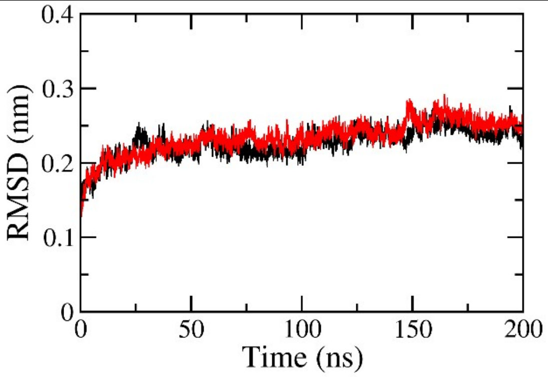	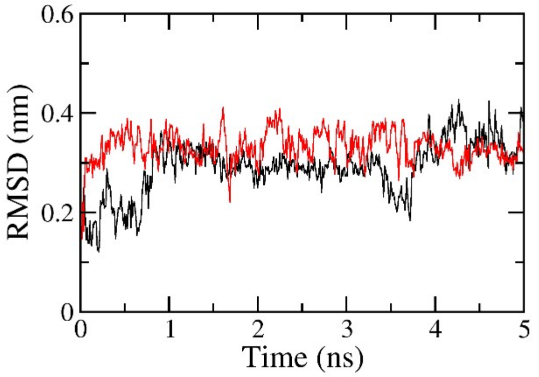
4	**22**	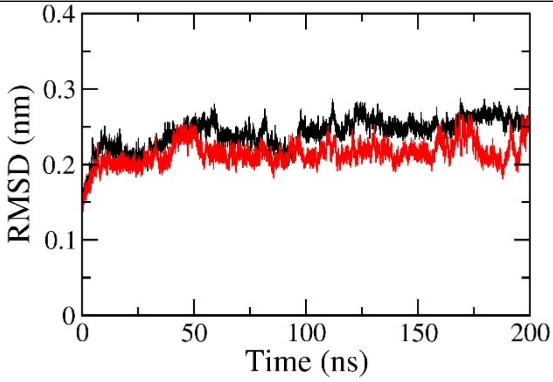	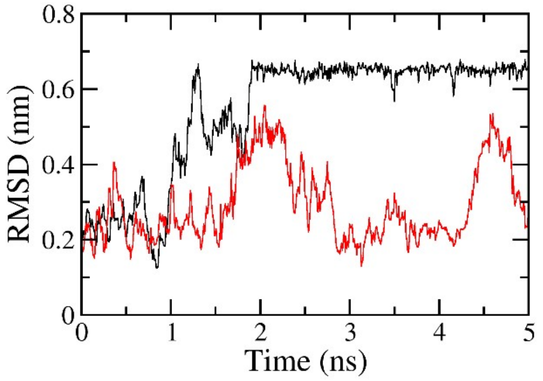
5	**25**	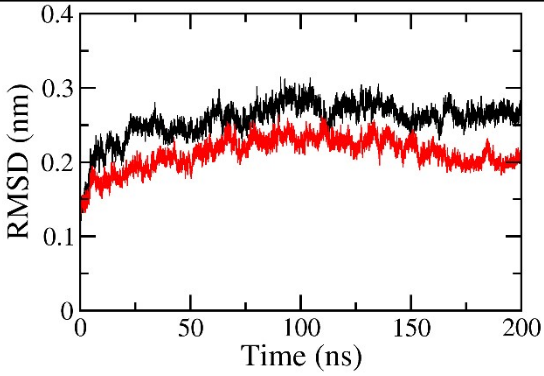	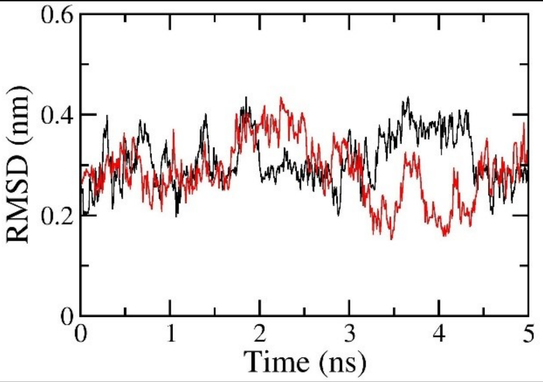

6	**26**	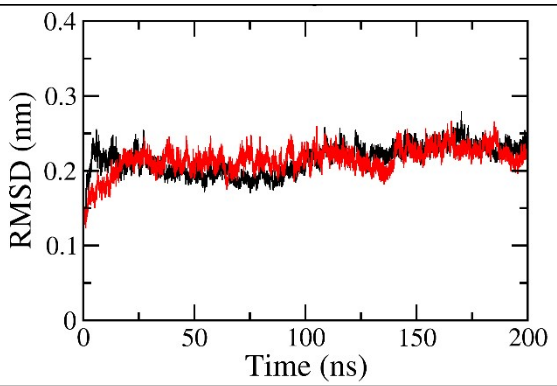RMSD (nm)<br>Time (ns)<br>0<br>0.1<br>0.2<br>0.3<br>0.4<br>50<br>100<br>150<br>200	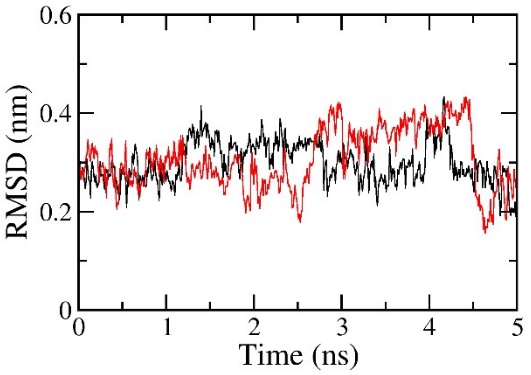RMSD (nm)<br>Time (ns)<br>0<br>0.2<br>0.4<br>0.6<br>1<br>2<br>3<br>4<br>5

Table S4: RMSF and Radius of Gyration (Rg) of all residues over 200 ns MD simulation

N⁰	Compound	RMSF	Gyration
1	**12**	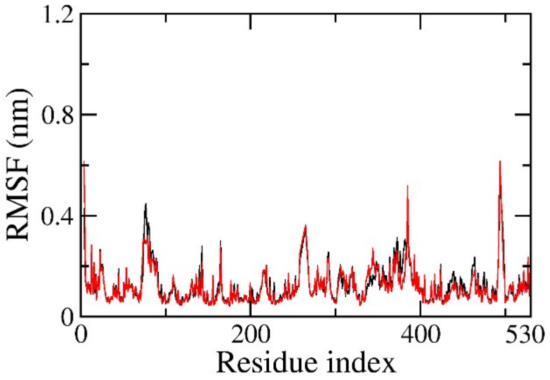	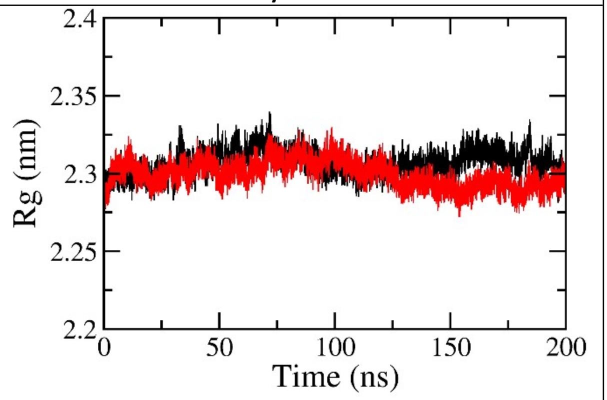
2	**15**	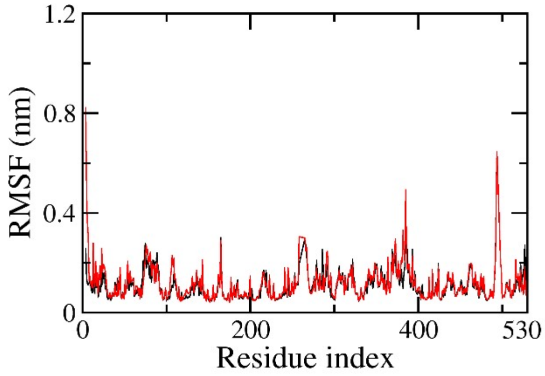	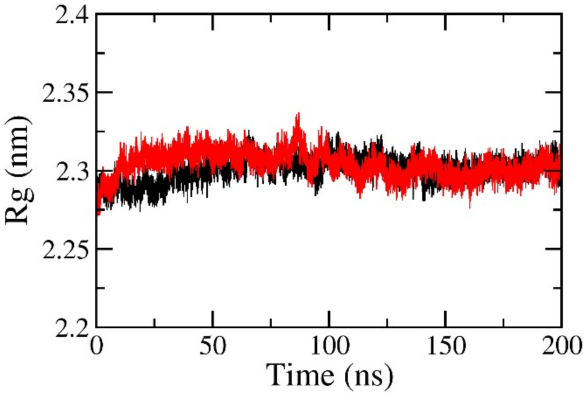
3	**18**	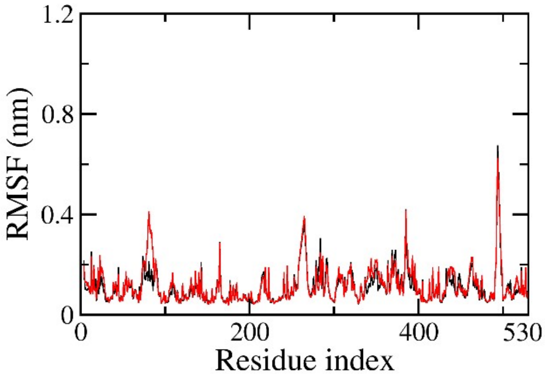	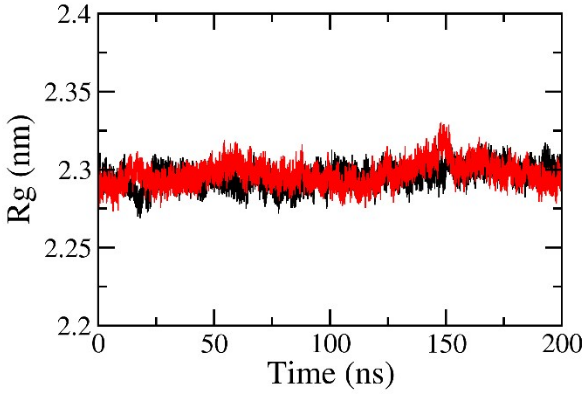
4	**22**	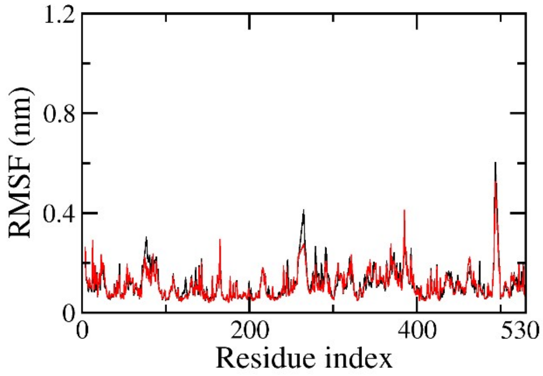	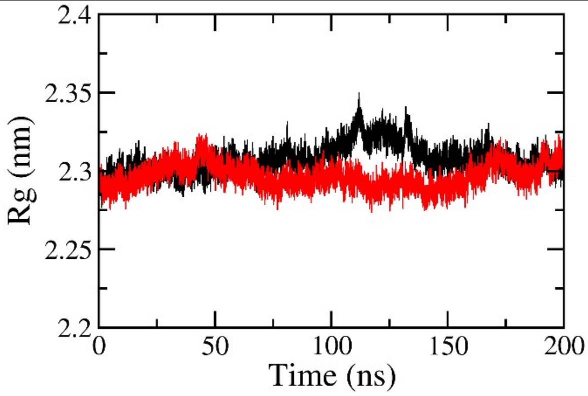

5	**25**	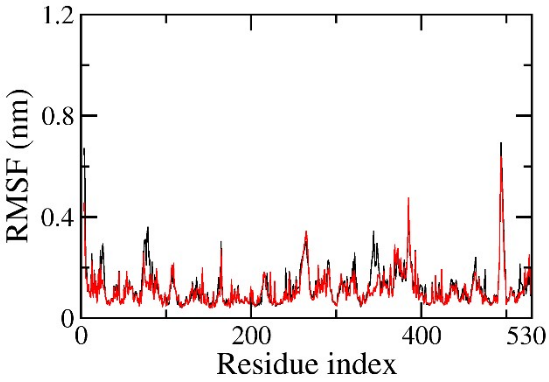	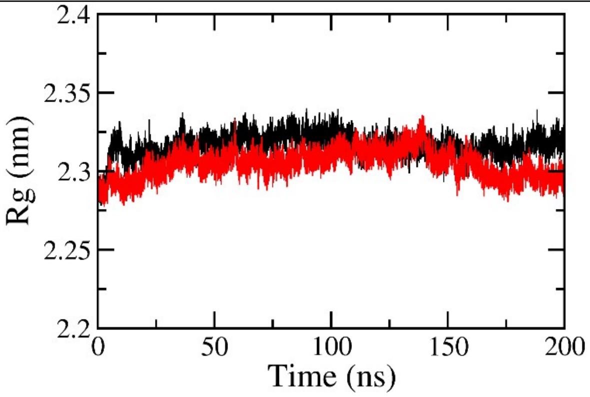
6	**26**	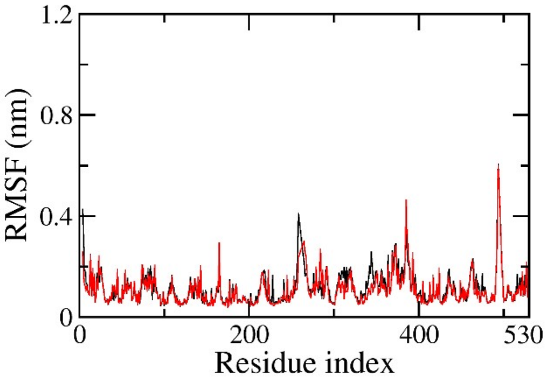	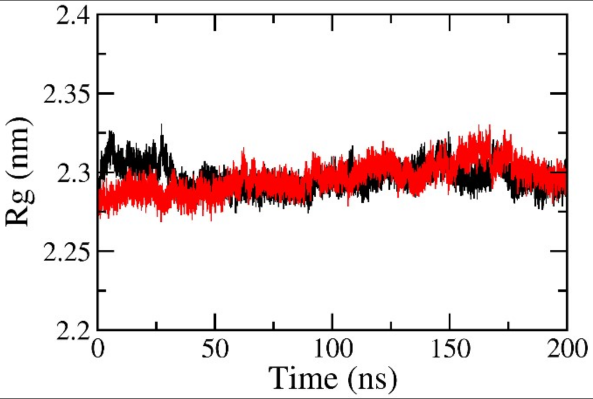

Table S5: Some ADMET values of compounds **12** and Galantamine caculated by PreADMET web-server

Compounds/Values	BBB	logBB	HIA (%)	Caco2	algae_at	Carcino_Mouse
**Compound 12**	0.107584	-0.968252	97.155198	20.7634	0.022619	negative
**Galantamine**	0.306811	-0.513129	96.172755	21.0629	0.342056	positive

BBB: Blood-Brain Barrier (BBB) penetration; logBB: log of BBB; HIA: Human Intestinal Absorption; algae_at: Acute algae toxicity
